# Choline Chloride/Urea – Promoted Iodocyclization of 3‐Alkynylthiophene‐2‐carboxamides: A Green Synthesis of 4‐Iodo‐7*H*‐thieno[2,3‐*c*]pyran‐7‐imines and Their Coupling Reactions in Deep Eutectic Solvents

**DOI:** 10.1002/chem.202500081

**Published:** 2025-04-16

**Authors:** Raffaella Mancuso, Patrizio Russo, Ida Ziccarelli, Melania Lettieri, Angela Altomare, Matteo Tiecco, Edoardo Mosconi, Tommaso Moretti, Bartolo Gabriele

**Affiliations:** ^1^ Laboratory of Industrial and Synthetic Organic Chemistry (LISOC), Department of Chemistry and Chemical Technologies University of Calabria Arcavacata di Rende Italy; ^2^ Institute of Crystallography National Research Council Bari Italy; ^3^ School of Pharmacy, ChIP Research Center University of Camerino Camerino Italy; ^4^ Computational Laboratory for Hybrid and Organic Photovoltaics Istituto CNR di Scienze e Tecnologie Chimiche (SCITEC‐CNR) c/o Department of Chemistry, Biology and Biotechnologies University of Perugia Perugia Italy; ^5^ Department of Chemistry Biology and Biotechnology University of Perugia Perugia Italy

**Keywords:** alkynes, amides, deep eutectic solvents, fused heterocycles, iodocyclization

## Abstract

A green approach to a previously unknown class of iodine‐containing heterocycles, namely 4‐iodo‐7*H*‐thieno[2,3‐*c*]pyran‐7‐imines, by iodocyclization of readily available 3‐alkynylthiophene‐2‐carboxamides in a deep eutectic solvent (DES) as both solvent and promoter, has been developed. We have found that, while the reaction with I_2_ and K_2_CO_3_ as the base in a classical volatile organic solvent proceeded to a very limited extent, the iodocyclization was successful when performed under neutral conditions in choline chloride/urea (1/2 molar ratio). Moreover, the DES could be efficiently recycled several times without a significant decrease in the product yield. The promoting effect of the DES has been studied in detail using computational methods. The possibility to further decorate the newly synthesized iodinated heterocycles in a DES solvent (choline chloride/glycerol, 1/2 molar ratio, for Sonogashira reaction, and betaine/glycerol, 1/2 molar ratio, for Suzuki‐Miyaura coupling) has been successfully assessed, and the solvent‐catalyst system was recycled several times without appreciable loss of activity. A computational study on the effect of the betaine/glycerol system in the Suzuki–Miyaura cross‐coupling has also been carried out, while the structures of representative products have been confirmed by X‐ray diffraction analysis.

## Graphical abstract

**New green routes to fused heterocycles**: Choline chloride (ChCl)/urea (1/2 mol/mol) deep eutectic solvent (DES) is used as both recyclable reaction medium and promoter for the base‐free iodocyclization of 3‐alkynylthiophene‐2‐carboxamides to 4‐iodo‐7*H*‐thieno[2,3‐c]pyran‐7‐imines. Recyclable DESs ChCl/Gly (1/2 mol/mol, Gly = glycerol) and Bet/Gly (1/2 mol/mol, Bet = betaine) are successfully used for subsequent Sonogashira and Suzuki‐Miyaura couplings, respectively.

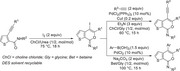

## 1 Introduction

The development of new synthetic approaches to high‐value‐added compounds using nonconventional solvents, less toxic, non‐volatile, non‐flammable, and more environmentally‐friendly than classical volatile organic compounds (VOCs), is one of the key principles of green chemistry.^[^ ^1^ ^]^ In this regard, deep eutectic solvents (DESs) have recently acquired increasing importance, owing to their versatility, availability, and the possibility to modulate their properties by the suitable combination of their components.^[^ ^2^ ^]^

**Scheme 1:**
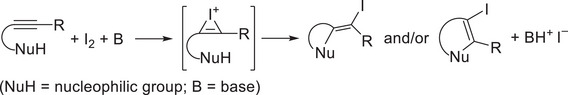
Iodocyclization of acetylenic derivatives bearing a suitable placed nucleophilic group.

On the other hand, iodocyclization of acetylenic substrates bearing a nucleophilic group in an appropriate position using I_2_ as the simplest iodine source is widely recognized as one of the most efficient, selective, and convenient approaches to iodine‐containing heterocyclic derivatives (Scheme 1),^[^ ^3^ ^]^ which can be further functionalized by palladium‐catalyzed cross‐coupling reactions. From the point of view of sustainability, however, this approach suffers from the need for the use of a sacrificial base necessary to buffer the hydrogen iodide that is formally released during the process. Moreover, iodocyclizations have been classically performed in VOCs as solvents, methylene chloride in particular.

**Scheme 2:**
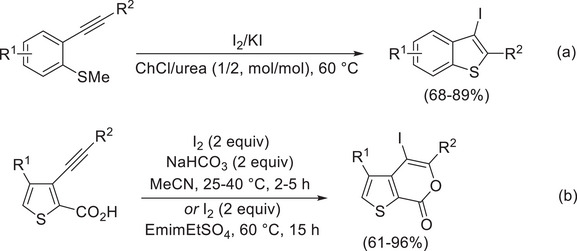
Iodocyclization of (a) 2‐methylthiophenylacetylenes to 3‐iodobenzothiophenes in ChCl/urea (1/2, mol/mol) as solvent^[^ ^4^ ^]^ and (b) 3‐alkynylthiophene‐2‐carboxylic acids to 4‐iodo‐7*H*‐thieno[2,3‐*c*]pyran‐7‐ones in MeCN or EmimEtSO_4_.^[^ ^6^ ^].^

We recently reported that iodocyclization of 2‐methylthiophenylacetylenes to 3‐iodobenzothiophenes can be successfully performed in a DES (ChCl/urea 1/2, mol/mol, in particular; ChCl = choline chloride) as reaction medium (Scheme 2a).^[^ ^4^ ^]^ While the process was already known in a classical VOC solvent,^[^ ^5^ ^]^ the use of the recyclable DES allowed us to perform it in a more sustainable manner.^[^ ^4^ ^]^ In another recent work, 3‐alkynylthiophene‐2‐carboxylic acids were iodocyclized either in a classical solvent such as MeCN (with I_2_ as the iodine source and NaHCO_3_ as the base) or in a basic ionic liquid (such as 1‐ethyl‐3‐methylimidazolium ethyl sulfate, EmimEtSO_4_) to give 4‐iodothienopyranone derivatives (Scheme 2b).^[^ ^6^ ^]^

**Scheme 3:**
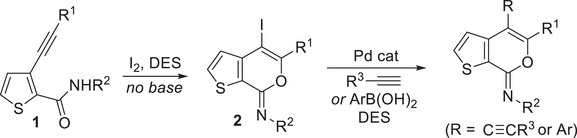
*This work*: Iodocyclization of 3‐alkynylthiophene‐2‐carboxamides **1** under neutral conditions to give (*Z*)‐4‐iodo‐7*H*‐thieno[2,3‐*c*]pyran‐7‐imines **2** using a DES as solvent and promoter and further functionalization of **2** by Sonogashira and Suzuki‐Miyaura cross‐couplings in a DES.

In this work, we have studied the possibility of performing the iodocyclization of readily available 3‐alkynylthiophene‐2‐carboxamides **1** under neutral conditions using a DES not only as a green and recyclable solvent but also as a promoter. Moreover, a process like this would allow the synthesis of a previously unknown class of iodine‐containing heterocyclic derivatives, namely (*Z*)‐4‐iodo‐7*H*‐thieno[2,3‐*c*]pyran‐7‐imines **2** ^[^ ^7^ ^]^ (Scheme 3), as it is known that, under neutral or slightly basic conditions, amides are nucleophilic at the oxygen atom rather than nitrogen.^[^ ^8^ ^]^ Finally, the possibility to further decorate the iodocyclization products **2** by Pd‐catalyzed Sonogashira and Suzuki–Miyaura cross‐couplings, still using DESs as recyclable reaction media, was another scope of the present study (Scheme 3).

## 2 Results and Discussion

### 2.1 Iodocyclization of 3‐Alkynylthiophene‐2‐Carboxamides **1** in ChCl‒Urea to Give (*Z*)‐4‐Iodo‐7*H*‐Thieno[2,3‐*c*]Pyran‐7‐Imines **2**

To begin with, we performed the iodocyclization of *N*‐butyl‐3‐(hex‐1‐yn‐1‐yl)thiophene‐2‐carboxamide **1a** under classical conditions,^[^ ^3^ ^]^ with I_2_ (2 equiv) in CH_2_Cl_2_ at 100°C (sealed tube) for 15 h in the presence of 2 equiv of K_2_CO_3_ as the base. Analysis of the reaction mixture evidenced a substrate conversion of 73% and the formation of (*Z*)‐*N*,5‐dibutyl‐4‐iodo‐7*H*‐thieno[2,3‐*c*]pyran‐7‐imine **2a** in 24% isolated yield (Table 1, Entry 1). Although the product was obtained in rather low yield, this result confirmed the possibility of synthesizing iodine‐containing 7*H*‐thieno[2,3‐*c*]pyran‐7‐imines **2** by an iodocyclization approach. The yield was even lower in another classical volatile organic solvent, such as acetonitrile (Table 1, Entry 2). Performing the reactions at 60°C led to inferior results (Table 1, Entries 3 and 4). In the absence of the base, practically no reaction took place (Table 2, Entries 5 and 6).

**Table 1:** Iodocyclization of *N*‐butyl‐3‐(hex‐1‐yn‐1‐yl)thiophene‐2‐carboxamide **1a** to (*Z*)‐*N*,5‐dibutyl‐4‐iodo‐7*H*‐thieno[2,3‐*c*]pyran‐7‐imine **2a** under different conditions.^[^ ^a^ ^]^

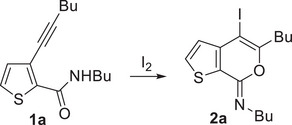						
Entry	I_2_ [equiv]	Solvent	*T* [°C]	time [h]	Conversion of **1a** [%]^[^ ^b^ ^]^	Yield of *2a* [%]^[^ ^[c]^, ^[d]^ ^]^
1^[^ ^e^ ^]^	2	CH_2_Cl_2_	100	15	73	24
2^[^ ^e^ ^]^	2	MeCN	100	15	74	17
3^[^ ^e^ ^]^	2	CH_2_Cl_2_	60	15	61	7
4^[^ ^e^ ^]^	2	MeCN	60	15	63	6
5	2	CH_2_Cl_2_	60	15	25	Traces
6	2	MeCN	60	15	18	Traces
7	2	ChCl/urea^[^ ^f^ ^]^	60	15	85	54
8	2	ChCl/urea^[^ ^f^ ^]^	60	18	100	67
9	1.5	ChCl/urea^[^ ^f^ ^]^	60	18	100	52
10	2	ChCl/Gly^[^ ^g^ ^]^	60	18	96	40
11	2	ChCl/EG^[^ ^h^ ^]^	60	18	97	45
12	2	ChCl/urea^[^ ^f^ ^]^	75	18	100	79
13	2	Bet/Gly^[^ ^i^ ^]^	75	18	30	23
14	2	Gly	75	18	100	15
15	2	Water	75	18	100	50

Abbreviations: Bet = betaine; ChCl = choline chloride; EG = ethylene glycol; Gly = glycerol.

^[a]^ Starting substrate concentration was 0.15 mmol of **1a** per mL of solvent.

^[b]^ Based on unreacted **1a**, after isolation from the reaction mixture.

^[c]^ Isolated yield, based on starting **1a**.

^[d]^ The formation of heavy compounds (chromatographically immobile materials) accounted for the difference between **2a** yields and substrate conversion.

^[e]^ The reaction was carried out also in the presence of 2 equiv of K_2_CO_3_.

^[f]^ ChCl/Urea molar ratio = 1/2.

^[g]^ ChCl/Gly molar ratio = 1/2.

^[h]^ ChCl/EG molar ratio = 1/2.

^[i]^ Bet/Gly molar ratio = 1/2.

**Table 2:** Iodocyclization of 3‐alkynylthiophene‐2‐carboxamides **1** under neutral conditions with ChCl/Urea (1/2 molar ratio; ChCl = choline chloride) as solvent and promoter to give (*Z*)‐4‐iodo‐7*H*‐thieno[2,3‐*c*]pyran‐7‐imines **2**.^[a]^

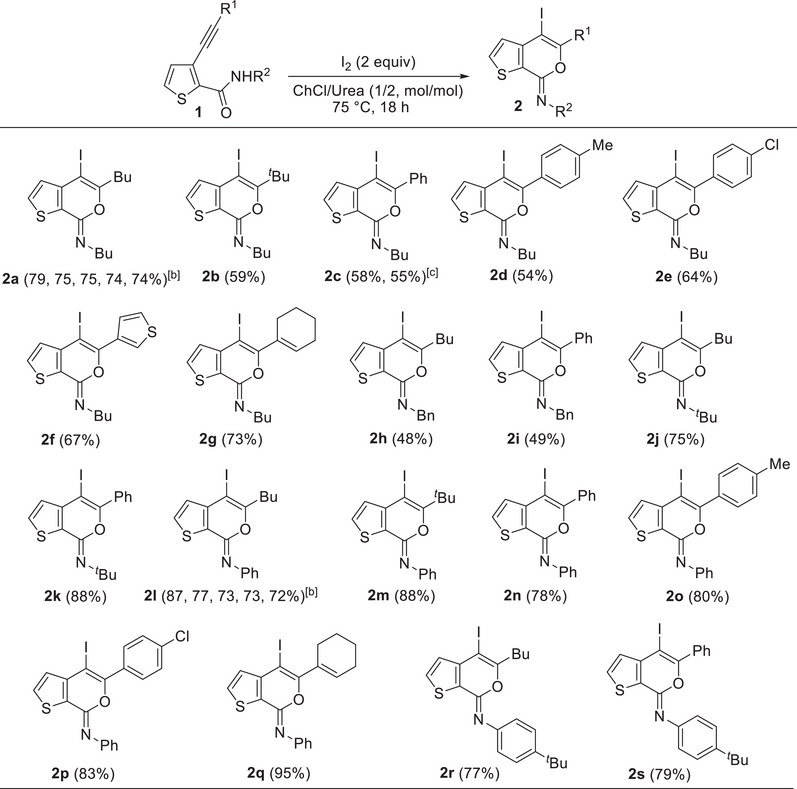

^[a]^ All reactions were carried out at 75°C for 18 h in ChCl/Urea (1/2 molar ratio) as the solvent, with 2 equiv of I_2_ and a starting substrate concentration of 0.15 mmol of **1** per mL of solvent. Yields of products **2** are isolated and based on starting **1**.

^[b]^ The first number refers to the isolated yield of **2** obtained from the parent experiment, the subsequent numbers to the yields obtained after solvent recycles and product extraction with Et_2_O (see the Experimental Section for details).

^[c]^ The first number refers to the isolated yield of **2c** obtained after product extraction with Et_2_O, the second number to the isolated yield of **2c** obtained after product extraction with 2‐methyltetrahydrofuran.

Very interestingly, however, when the reaction was carried out at 60°C in ChCl/urea (1/2 molar ratio) as the solvent in the absence of an external base, **2a** was isolated in 54% yield at 85% substrate conversion (Table 1, Entry 7). After 18 h, **1a** conversion was quantitative, and the yield of **2a** reached 67% (Table 1, Entry 8). This result clearly showed that the DES solvent was able to promote the iodocyclization process, under neutral conditions without any need for a sacrificial base. The results obtained were less satisfactory when a lower amount of I_2_ was employed (Table 1, Entry 9), or when the urea component of the DES was replaced by glycerol (Gly; Table 1, Entry 10) or ethylene glycol (EG; Table 1, Entry 11). On the other hand, a significant improvement in the product yield (79%) was observed by conducting the reaction in ChCl/urea at 75°C instead of 60°C (Table 1, Entry 12). We also performed the process under the optimized conditions of Entry 12 (Table 1) in another DES, such as Bet/Gly (1/2 molar ratio, Bet = betaine) as well as other environmentally friendly solvents, like pure glycerol or even water. The results obtained, shown in Table 1, Entries 13–15, were, however, less satisfactory with respect to those obtained with ChCl/Urea, the yields of **2a** being 23% in Bet/Gly (Table 1, Entry 13), 15% in Gly (Table 1, Entry 14), and 50% in water (Table 1, Entry 15).

We next proceeded to assess the recyclability of our ChCl/urea (1/2 molar ratio) DES as both solvent and promoter. Thus, after extraction of the product with diethyl ether (see the Experimental Section for details), fresh **1a** and I_2_ were added to the DES residue, and the mixture was allowed to react again at 75°C for 18 h. As can be seen from the results shown in Table 2, Entry 1, a 75% isolated yield of **2a** was achieved, which remained practically unchanged after further 3 recycles.

**Figure 1:**
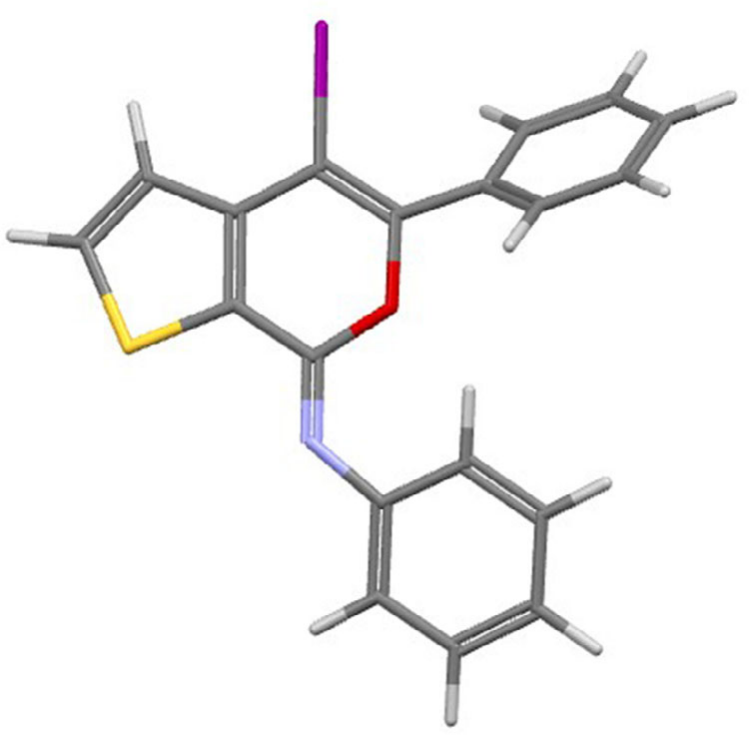
X‐ray molecular structure of (*Z*)‐4‐iodo‐*N*,5‐diphenyl‐7*H*‐thieno[2,3‐*c*]pyran‐7‐imine **2n**.

Under the conditions optimized for **1a**, other *N*‐alkyl‐substituted substrates bearing different groups on the triple bond **1b‐k** were smoothly converted into the corresponding iodothienopyranimines **2b‐k** in yields ranging from 48% to 88% (Table 2). We have also assessed the possibility of using more environmentally friendly 2‐methyltetrahydrofuran (2‐MeTHF) rather than diethyl ether as the solvent for product extraction, with comparable results (for example, using 2‐MeTHF, product **2c** was obtained in 55% isolated yield, similar to 58% observed with Et_2_O, see Table 2). Yields were lower for products bearing a butyl or a benzyl group on nitrogen with respect to *N*‐*tert*‐butyl‐substituted ones. This probably reflects a lower tendency to undergo degradation when the imino moiety was substituted with a sterically hindered group.^[^ ^9^ ^]^ Higher stability of the imino group when substituted with an aromatic ring^[^ ^10^ ^]^ also accounted for the high yields (77%–95%) observed with substrates **1l‐s**, as seen in Table 2. The structure of a representative product, namely (*Z*)‐4‐iodo‐*N*,5‐diphenyl‐7*H*‐thieno[2,3‐*c*]pyran‐7‐imine **2n**, was confirmed by XRD analysis (Figure 1; see the Supporting Information for full XRD data). The DES recyclability was assessed again in the case of the reaction of 3‐(hex‐1‐yn‐1‐yl)‐*N*‐phenylthiophene‐2‐carboxamide **1l**. As can be seen from the results shown in Table 2, the product yields decreased to 77% after the first recycle, and remained as high as 72% even after the 4th recycle.

### 2.2 Computational Study on the Promoting Effect of ChCl‒Urea in the Iodocyclization of 3‐Alkynylthiophene‐2‐Carboxamides 1 to (*Z*)‐4‐Iodo‐7*H*‐Thieno[2,3‐*c*]Pyran‐7‐Imines **2**

To gain insight into the effect of the DES on the iodocyclization mechanism, we performed a series of theoretical simulations based on density functional theory (DFT). We initially analyzed the reagents in their interacting complex structure (**R**), products (**P**), transition states (**TS**), and possible intermediates (**I**) in different environments: in vacuum (VAC), in implicit solvent (CPCM),^[^ ^11^ ^]^ in explicit classical solvent (CH_2_Cl_2_), and with DES (ChCl/Urea 1/2). Computational details are reported in Supporting Information. In this way, from the overall picture, we were able to identify the specific role of the DES in determining the important reaction activity experimentally found in Table 1. Regarding the reaction mechanism, we analyzed the possibility of a concerted mechanism, where we have the simultaneous attack of the I_2_ and of the carboxamide oxygen to the triple bond group providing the cyclic transition state (**TS**), and a stepwise mechanism. In the latter, we have the initial formation of the intermediate (**I**) species from the I_2_ attack to the triple bond, and the subsequent cyclization form deriving from the oxygen attack to the carbon atoms of **I**. By looking at the energetics in the solvent reported in Table 3, we can easily notice that the preferred mechanism in all cases is the concerted one, since the intermediate **I** presents a higher energy with respect to the reactants and it is even not stable for the DES case where is not possible to find a minimum energy structure.

**Table 3:** Energetics of the iodocyclization of 3‐alkynylthiophene‐2‐carboxamides **1** to iodothienopyranimines **2** in kcal/mol.

	Δ*G*_solv_ (kcal/mol)			
Environment	DES + CPCM	CH_2_Cl_2_ + CPCM	CPCM	VAC
R	0.0	0.0	0.0	0.0
**TS** (concerted)	10.0	14.9	15.91	14.26
P	−44.5	−39.2	−40.58	−13.95
**I** (stepwise)	No min.	11.41	5.98	12.57

**Figure 2:**
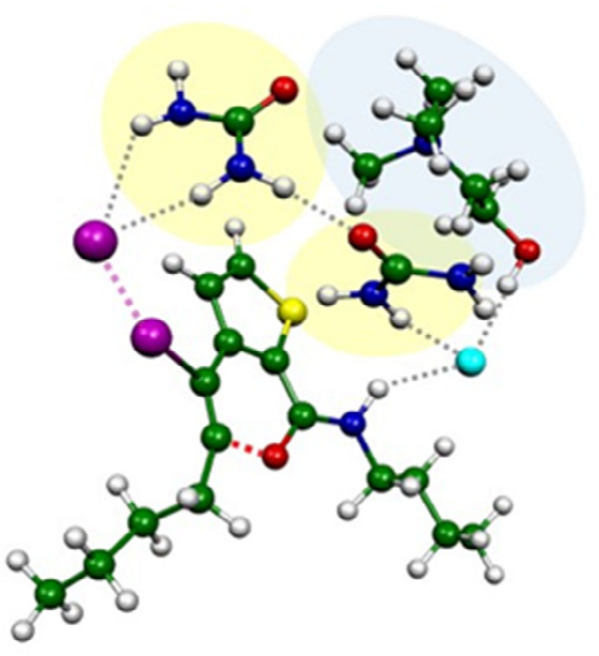
TS for the iodocyclization of 3‐alkynylthiophene‐2‐carboxamides **1** to iodothienopyranimines **2** (Color legend: C = green; O = red; N = blue; Cl = cyan; I = magenta; S = yellow; H = white).

More interestingly, regarding the concerted reaction mechanism path, the DES provides the lowest activation energy in line with the high activity found in Table 1. This is due to the synergic role of the ChCl and urea in stabilizing the TS through specific interactions that a classical solvent such as CH_2_Cl_2_ cannot provide. In particular, as we can see in Figure 2, the synergic role of the hydrogen bond induces the stabilization of the I^−^ leaving group by the urea molecules that are also interacting with the Cl^−^ coming from ChCl. Moreover, the Cl^−^ contributes also in helping the deprotonation process by the hydrogen bond interaction. To further confirm this aspect, we calculated the deprotonation process to all possible base centers in the model finding that the preferred protonation site is the urea oxygen.

### 2.3 Sonogashira and Suzuki–Miyaura Cross‐Coupling Reactions of 4‐Iodo‐7*H*‐Thieno[2,3‐*c*]Pyran‐7‐Imines **2** in DESs

ChCl/Gly (1/2 molar ratio) turned out to be an excellent solvent for performing the Sonogashira cross‐coupling of 4‐iodo‐7*H*‐thieno[2,3‐*c*]pyran‐7‐imines **2** to give 4‐aryl‐7*H*‐thieno[2,3‐*c*]pyran‐7‐imines **4**. Thus, with *N*‐butyl‐3‐(hex‐1‐yn‐1‐yl)thiophene‐2‐carboxamide **1a** and phenylacetylene **3a** as coupling partners and Et_3_N as the base, in the presence of PdCl_2_(PPh_3_)_2_ as the catalytic precursor and CuI as cocatalyst (**2l**:Pd:CuI:Et_3_N molar ratio = 1:0.1:0.2:3), at 60°C for 15 h, (*Z*)‐*N*,5‐dibutyl‐4‐(phenylethynyl)‐7*H*‐thieno[2,3‐c]pyran‐7‐imine **4aa** was obtained in 71% isolated yield (Table 4). The reaction medium, still containing the catalyst dissolved in it, could then be conveniently recycled after product extraction, by adding fresh **2a** and **3a** and repeating the coupling procedure, without a significant decrease in **4aa** yield even after the 4th recycle (Table 4). Other **2**/**3** combinations also led to fair to good results, as shown in Table 4. The DES‐catalyst recyclability was assessed again in the reaction between (*Z*)‐4‐iodo‐*N*,5‐diphenyl‐7*H*‐thieno[2,3‐*c*]pyran‐7‐imine **2n** and phenylacetylene **3a** to give (*Z*)‐*N*,5‐diphenyl‐4‐(phenylethynyl)‐7*H*‐thieno[2,3‐*c*]pyran‐7‐imine **4na** (Table 4).

**Table 4:** Sonogashira coupling of (*Z*)‐4‐iodo‐7*H*‐thieno[2,3‐*c*]pyran‐7‐imines **2** and terminal alkynes **3** in ChCl/Gly (1/2 molar ratio; ChCl = choline chloride, Gly = glycerol) as the solvent to give 4‐alkynyl‐7*H*‐thieno[2,3‐c]pyran‐7‐imines **4**.^[a]^

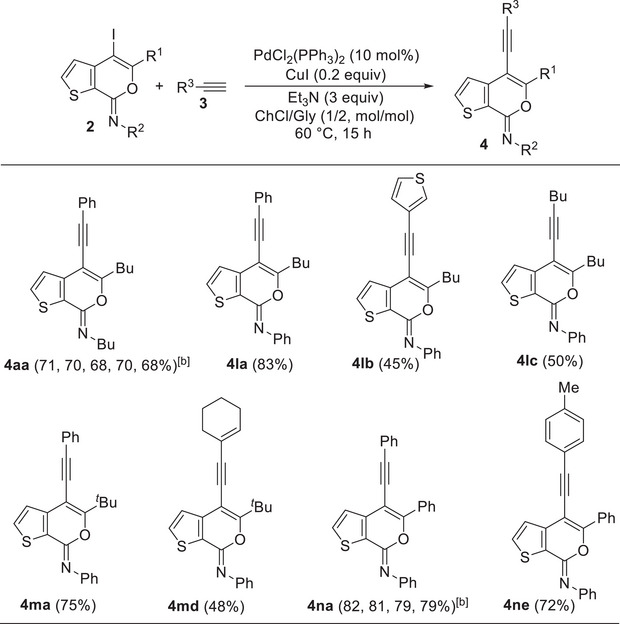

^[a]^ All reactions were carried out at 60°C for 15 h in ChCl/Gly (1/2 molar ratio) as the solvent, with 2 equiv of **3** and 3 equiv of Et_3_N, in the presence of 10 mol% of PdCl_2_(PPh_3_)_2_, 20 mol% of CuI, and with a starting substrate concentration of 0.18 mmol of **2** per mL of solvent. Yields of products **4** are isolated and based on starting **2**.

^[b]^ The first number refers to the isolated yield of **4** obtained from the parent experiment, the subsequent numbers to the yields obtained after solvent recycles and product extraction with Et_2_O (see the Experimental Section for details).

**Scheme 4:**
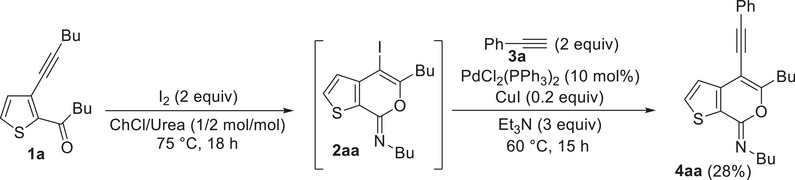
One‐pot iodocyclization‐Sonogashira cross‐coupling protocol leading to **4aa**.

We also assessed the possibility of developing a one‐pot protocol by allowing to react crude product **2a**, obtained by iodocyclization of **1a** under the optimized conditions (see Table 2), with phenylacetylene **3a** without its isolation and purification from the iodocyclization reaction mixture. However, as shown in Scheme 4, the isolated yield of **4aa** was significantly lower (28%) with respect to the overall yield (56%) obtained by performing the two reactions separately (iodocyclization, **2a** yield = 79%, see Table 2, and then Sonogashira coupling, **4aa** yield = 71% based on purified **2a**, see Table 4).

**Figure 3:**
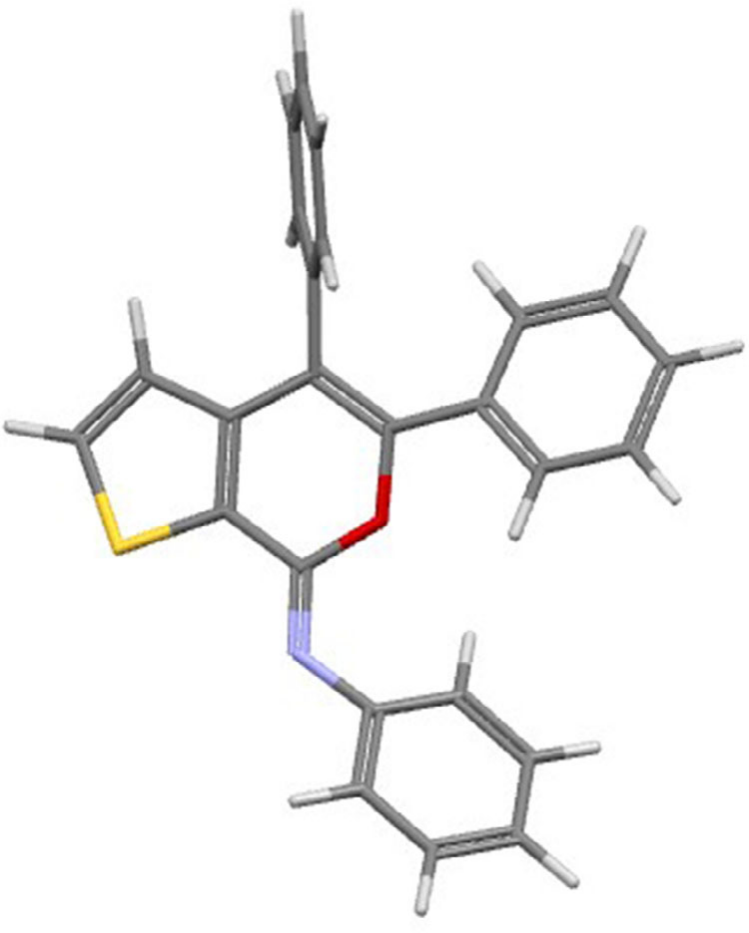
X‐ray molecular structure of (*Z*)‐*N*,4,5‐triphenyl‐7*H*‐thieno[2,3‐*c*]pyran‐7‐imine **6na**.

Interestingly, the Suzuki–Miyaura coupling of (*Z*)‐5‐butyl‐4‐iodo‐*N*‐phenyl‐7*H*‐thieno[2,3‐*c*]pyran‐7‐imine **2l** with phenylboronic acid **5a**, carried out using PdCl_2_ as the catalytic precursor and Na_2_CO_3_ as the base (**2l**:Pd:Na_2_CO_3_:**5a** molar ratio = 1:0.1:2:1.5) at 100°C for 15 h, did not work when performed in ChCl/Gly, ChCl/EG, or ChCl/Urea as the solvent (Table 5, Entries 1–3). This is despite the fact that several studies in the literature have reported the successful use of ChCl‐based DESs for this kind of transformation.^[^ ^12^ ^]^ However, the reaction could be performed in another DES with a different HBA as the reaction medium, that is, Bet/Gly (1/2 molar ratio), with the formation of the desired coupling product (*Z*)‐5‐butyl‐*N*,4‐diphenyl‐7*H*‐thieno[2,3‐*c*]pyran‐7‐imine **6la** in 68% isolated yield (Table 5, Entry 4). With *p*‐tolylboronic acid **5b**, the product yield was 64% (Table 5, Entry 5). Fair yields of the corresponding coupling products **6na** and **6nc** (71% and 60%, respectively) were also obtained from (*Z*)‐4‐iodo‐*N*,5‐diphenyl‐7*H*‐thieno[2,3‐*c]*pyran‐7‐imine **2n** with phenylboronic acid **5a** and with furan‐3‐ylboronic acid **5c** (Table 5, Entries 6 and 7, respectively). The recyclability of the DES‐catalyst system was further confirmed in the experiments leading to **6na** (Table 5, Entry 6), and the structure of this product was confirmed by XRD analysis (Figure 3).

**Table 5:** Suzuki–Miyaura coupling of (*Z*)‐4‐iodo‐7*H*‐thieno[2,3‐*c*]pyran‐7‐imines **2** and arylboronic acids **5** in DESs to give 4‐aryl‐7*H*‐thieno[2,3‐*c*]pyran‐7‐imines **6**.^[a]^

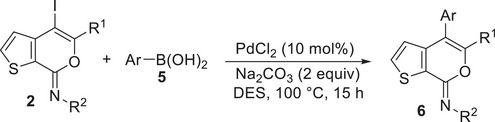					
Entry	2	5	DES	6	Yield of 6 [%]^[^ ^b^ ^]^
1	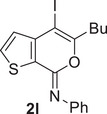	Ph−B(OH)_2_ **5a**	ChCl/Gly		NR
2	**2l**	**5a**	ChCl/EG		NR
3	**2l**	**5a**	ChCl/urea		NR
4	**2l**	**5a**	Bet/Gly	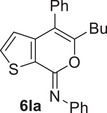	68
5	**2l**	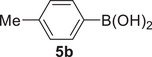	Bet/Gly	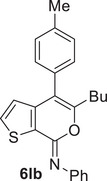	64
6	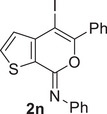	**5a**	Bet/Gly	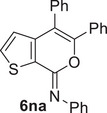	71 (69, 68, 68, 66)
7	**2n**	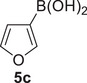	Bet/Gly	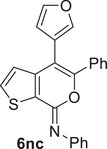	60

Abbreviations: Bet = betaine; ChCl = choline chloride; EG = ethylene glycol; Gly = glycerol; NR = no reaction.

^[a]^ All reactions were carried out at 100°C for 15 h in DES (1/2 molar ratio) as the solvent, with 1.5 equiv of **5** and 2 equiv of Na_2_CO_3_, in the presence of 10 mol% of PdCl_2_ and with a starting substrate concentration of 0.20 mmol of **2** per mL of solvent.

^[b]^ Isolated yield, based on starting **2**. Data in parentheses refer to the yields obtained after solvent recycles (see the Experimental Section for details).

### 2.4 Computational Study on the Effect of Bet/Gly in the Suzuki–Miyaura Cross‐Coupling of **2** with **5** to Give **6**

**Figure 4:**
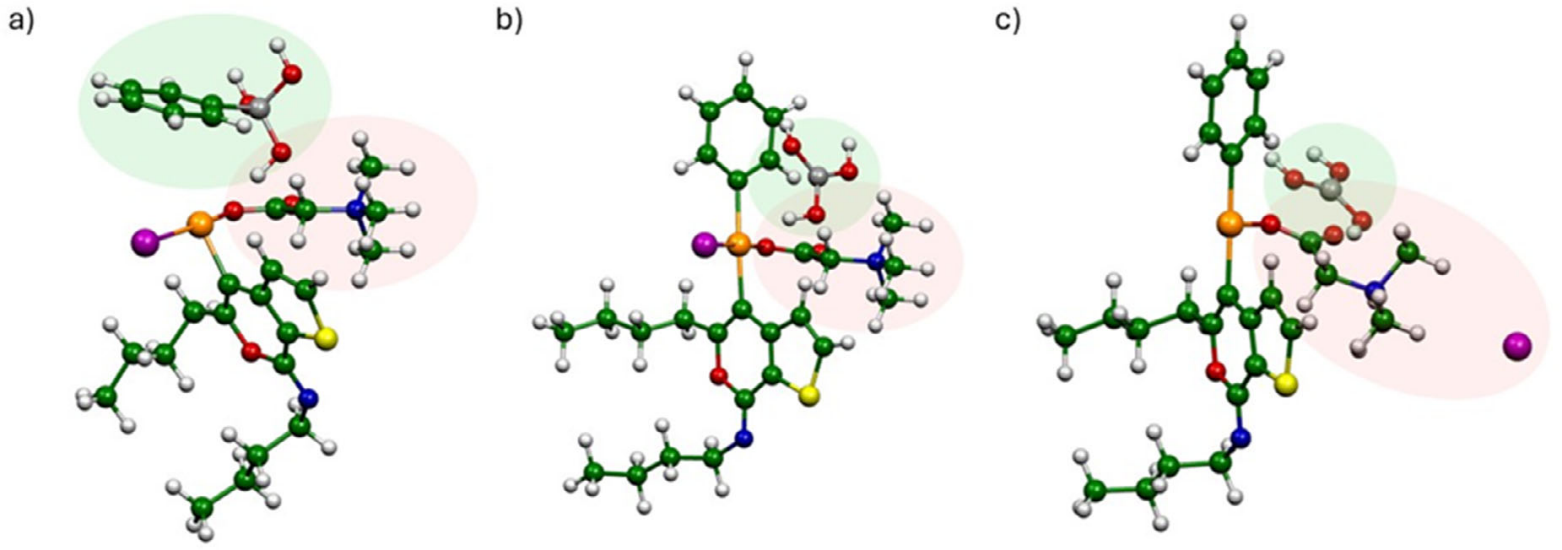
(a) Ar′PdI + ArB(OH)_3_^−^, (b) Ar′PdAr + H_3_BO_3_, (c) Ar′PdAr + H_3_BO_3_ + I^−^ from the Suzuki–Miyaura cross‐coupling of **2** with **5** to give **6** (color legend: C = green; O = red; N = blue; I = magenta; S = yellow; H = white; Pd = orange; B = grey).

The computational analysis was pointed to understand the role of betaine in promoting the limiting step of the Suzuki–Miyaura cross‐coupling. In particular, the crucial step of the reaction is associated with the exchange of Ar′PdI [deriving from the oxidative addition of **2** to Pd(0)] with ArB(OH)_3_^−^. We moved to simulate this step to determine the geometry rearrangement provided by the presence of Bet starting from the Ar′PdI + ArB(OH)_3_^−^complex (see Figure 4a). In terms of energetics, we evaluated the total energy in the solvent of the whole reagent and product complexes and then we further decomposed the process by simulating the initial exiting of the H_3_BO_3_ and the Ar group (─C_6_H_5_) attached to Pd, Figure 4b, and then the subsequent exiting of the I^−^ (see Figure 4c). We simulated this process both in the presence and in the absence of Bet. As reported in Table 6, the reaction carried out in Bet's presence has an overall spontaneous Δ*G*_solv_ of −12.3 kcal/mol, while in the absence of Bet, the reaction is not spontaneous (Δ*G*_solv_ = +7.5 kcal/mol). Notably, as we can see from Figure 4, the role of Bet in this case is clearly to complex the Pd atoms and thanks to its zwitterionic nature also to help the exiting of the I^−^ in stabilizing the leaving group with the ─N(CH_3_)^+^ moiety. The importance of I^−^ stabilization is clearly remarked by the value of the first reaction that provides only the bonding of the ─C_6_H_5_ group to Pd that is spontaneous with and without Bet. This further confirms that the Bet stabilizes the product through the stabilization of the I^−^ leaving group. To verify even further the role of the Bet in favoring the exiting of the I^−^ (Ar′PdAr + H_3_BO_3_ + I^−^), we have also simulated the process by replacing Bet with the choline cation (see Figure S5), obtaining a positive Δ*G*_solv_ value of +10.9 kcal/mol. This result confirms the important role of betaine, which through the synergic effect between iodide elimination (by interaction with the −NMe_3_^+^ moiety) and palladium coordination (by the carboxylate moiety) leads instead to a spontaneous exchange of Ar′PdI with ArB(OH)_3_^−^ (Δ*G*_solv_, −12.3 kcal/mol).

**Table 6:** Energetics of the investigated steps in kcal/mol.

	Δ*G*_solv_ (kcal/mol)	
Environment	Bet + CPCM	CPCM
Ar′Pd‐I + ArB(OH)_3_^−^	0.0	0.0
Ar′PdAr + H_3_BO_3_	−23.9	−9.2
Ar′PdAr + H_3_BO_3_ + I^−^	−12.3	+7.5

## 3 Conclusions

In conclusion, we have developed a green route to a previously unknown class of iodinated‐fused heterocycles, namely, 4‐iodo‐7*H*‐thieno[2,3‐*c*]pyran‐7‐imines, by iodocyclization of readily available 3‐alkynylthiophene‐2‐carboxamides and with I_2_ as the iodinating agent.^[^ ^13^ ^]^ We have found that the process takes place to only a limited extent when carried out in a classical volatile organic solvent (such as CH_2_Cl_2_ or acetonitrile) in the presence of K_2_CO_3_ as a base. On the other hand, when performing the iodocyclization in a suitable DES (choline chloride/urea, 1/2 mol/mol, in particular) under neutral conditions, (*Z*)‐4‐iodo‐7*H*‐thieno[2,3‐*c*]pyran‐7‐imines were selectively obtained in fair to high yields (48%–95%), whose structure has been confirmed by XRD analysis of a representative product. The promoting effect of the DES has been studied and elucidated by computational methods. The possibility to recycle the DES solvent has also been demonstrated. The newly synthesized (*Z*)‐4‐iodo‐7*H*‐thieno[2,3‐*c*]pyran‐7‐imines have been successfully used as substrates for Sonogashira and Suzuki–Miyaura cross‐couplings, which could be conveniently conducted in another DES solvent, choline betaine/glycerol (1/2 molar ratio), with the possibility to recycle the solvent–catalyst system several times without appreciable loss of activity.

## 4 Experimental Section

### General Experimental Methods

The solvent and chemicals were reagent‐grade and were used without further purification. All reactions were analyzed by TLC on silica gel 60 F254 and by GLC using capillary columns with polymethylsilicone + 5% phenylsilicone as the stationary phase. Column chromatography was performed on silica gel 60 (70–230 mesh) or neutral alumina (90–170 mesh). Evaporation refers to the removal of solvent under reduced pressure. Melting points are uncorrected. ^1^H NMR and ^13^C NMR spectra were recorded at 25°C on a 500 MHz Spectrometer in CDCl_3_ with Me_4_Si as internal standard. Chemical shifts (*δ*) and coupling constants (*J*) are given in ppm and in Hz, respectively. IR spectra were taken with an FT‐IR spectrometer. Mass spectra were obtained using a GC‐MS apparatus at 70 eV ionization voltage (normal resolution) and by electrospray ionization mass spectrometry (ESI‐MS) (high resolution) with a UHD accurate‐mass Q‐TOF spectrometer equipped with a Dual AJS ESI source working in positive mode, and were recorded in the 150–1000 *m/z* range. The LC‐MS experimental conditions were as follows: N_2_ was employed as desolvation gas at 300°C and a flow rate of 9 L min^−1^. The nebulizer was set to 45 psig. The Sheat gas temperature was set at 350°C and a flow of 12 L min^−1^. A potential of 3.5 kV was used on the capillary for positive ion mode. The fragmentor was set to 175 V.

Deposition Numbers 2363245 (2n.cif) and 2363248 (6na.cif) contain the supplementary crystallographic data for this paper. These data are provided free of charge by the joint Cambridge Crystallographic Data Centre and Fachinformationszentrum Karlsruhe Access Structures service.

### Preparation of DESs and Substrates

Deep eutectic colvents ChCl/Gly (1/2 molar ratio),^[^ ^14^ ^]^ ChCl/Urea (1/2 mol/mol),^[^ ^14^ ^]^ ChCl/EG (1/2 molar ratio),^[^ ^14^ ^]^ and Bet/Gly (1/2 molar ratio)^[^ ^15^ ^]^ were prepared as we already reported. Starting materials **1** were prepared as described in the Supporting Information.

### Iodocyclization Procedure Leading to (*Z*)‐4‐Iodo‐7*H*‐Thieno[2,3‐*c*]Pyran‐7‐Imines **2** in DES

To a solution of **1** (0.30 mmol) (**1a**, 79.5 mg; **1b**, 79.8 mg; **1c**, 85.5 mg; **1d**, 90.0 mg; **1e**, 96.1 mg; **1f**, 87.5 mg; **1g**, 87.4 mg; **1h**, 90.0 mg; **1i**, 95.0 mg; **1j**, 79.3 mg; **1k**, 85.4 mg; **1l**, 85.6 mg; **1m**, 85.1 mg; **1n**, 91.6 mg; **1o**, 96.2 mg; **1p**, 101.0 mg; **1q,** 92.7 mg; **1r**, 102.2 mg; **1s**, 108.2 mg) in ChCl/Urea (1/2, mol/mol; 2.0 mL) was added I_2_ (152 mg, 0.60 mmol) (Table 2). The mixture was allowed to stir at 75°C (oil bath) for 18 h in a sealed tube and then cooled to room temperature. Et_2_O or 2‐methyltetrahydrofuran (3 mL) was added, the mixture heated to 60 °C, cooled to room temperature, and phases were separated. This extraction procedure was repeated for additional five times. Satd. aqueous Na_2_S_2_O_3_ (15 mL) was added to the collected ethereal phases, and the mixture was allowed to stir for 10 min. Phases were separated, the aqueous phase was extracted with Et_2_O or 2‐methyltetrahydrofuran (3 × 15 mL), while the DES phase was used for the next recycling experiments (see below). The collected organic layers were dried over Na_2_SO_4_. After filtration and evaporation of the solvent, products **2a‐s** were purified by column chromatography on alumina using as eluent pure hexane to 98:2 hexane‒AcOEt (isolated yields are given in Table 2). *E*‐factors are given in the Supporting Information.

### Recycling Procedure

To the DES residue obtained as described above was added a solution of **1** (0.30 mmol) and I_2_ (0.60 mmol) in Et_2_O (1.5 mL). The Et_2_O was removed under vacuum and then the same procedure described above was followed.

*(Z)‐N,5‐dibutyl‐4‐iodo‐7H‐thieno[2,3‐c]pyran‐7‐imine* (**2a**). Yield: 93.0 mg, starting from 79.5 mg of *N*‐butyl‐3‐(hex‐1‐yn‐1‐yl)thiophene‐2‐carboxamide **1a** (79%; Table 2). Yellow solid, mp = 40°C–41°C. IR (KBr): *ν* = 1659 (s), 1574 (m), 1443 (m), 1381 (m), 1258 (w), 1103 (w), 1018 (w), 748 (m) cm^−1^; ^1^H NMR (500 MHz, CDCl_3_): *δ* = 7.38 (d, *J* = 5.1, 1H), 7.05 (d, *J* = 5.1, 1H), 3.44 (t, *J* = 7.1, 2H), 2.76 (t, *J* = 7.3, 2H), 1.72‐1.57 (m, 4H), 1.48‐1.36 (m, 4H), 1.01‐0.92 (m, 6H); ^13^C NMR (125 MHz, CDCl_3_): *δ* = 156.5, 146.5, 141.4, 128.5, 128.3, 124.4, 65.2, 45.4, 34.7, 32.3, 28.8, 21.6, 20.3, 13.5, 13.4; GC‐MS: *m/z* = 389 (M^+^, 11), 360 (10), 346 (87), 332 (49), 262 (100), 207 (17), 178 (16), 149 (14), 136 (30), 122 (16); HRMS‐ESI (*m/z*): [(M+H)^+^] calcd for (C_15_H_21_INOS)^+^: 390.0383; found, 390.0417.

*(Z)‐5‐(tert‐Butyl)‐N‐butyl‐4‐iodo‐7H‐thieno[2,3‐c]pyran‐7‐imine* (**2b**). Yield: 70.0 mg, starting from 79.8 mg of *N*‐butyl‐3‐(3,3‐dimethylbut‐1‐yn‐1‐yl)thiophene‐2‐carboxamide **1b** (59%; Table 2). Yellow oil. IR (film): *ν* = 1668 (s), 1560 (w), 1479 (m), 1438 (m), 1365 (m), 1219 (w), 1094 (m), 1045 (w), 771 (m) cm^−1^; ^1^H NMR (500 MHz, CDCl_3_): *δ* = 7.37 (d, *J* = 5.0, 1H), 7.22 (d, *J* = 5.0, 1H), 3.46 (t, *J* = 7.2, 2H), 1.68‐1.55 (m, 2H), 1.55 (s, 9H), 1.47‐1.37 (m, 2H), 0.95 (t, *J* = 7.3, 3H); ^13^C NMR (125 MHz, CDCl_3_): *δ* = 160.6, 146.7, 143.7, 130.0, 128.3, 124.0, 62.8, 45.1, 39.2, 32.9, 29.1, 20.8, 14.0; GC‐MS: *m/z* = 389 (M^+^, 11), 360 (7), 346 (49), 332 (42), 276 (62), 262 (100), 248 (18), 206 (55), 178 (16), 150 (54), 122 (27); HRMS‐ESI (*m/z*): [(M+H)^+^] calcd for (C_15_H_21_INOS)^+^: 390.0383; found, 390.0378.

*(Z)‐N‐Butyl‐4‐iodo‐5‐phenyl‐7H‐thieno[2,3‐c]pyran‐7‐imine* (**2c**). Yield: 72.0 mg, starting from 85.5 mg *N*‐butyl‐3‐(phenylethynyl)thiophene‐2‐carboxamide **1c** (58%; Table 2). Yellow oil. IR (film): *ν* = 1669 (s), 1586 (w), 1490 (w), 1444 (m), 1355 (w), 1219 (w), 1075 (m), 1050 (m), 899 (w), 772 (m) cm^−1^; ^1^H NMR (500 MHz, CDCl_3_): *δ* = 7.73‐7.65 (m, 2H), 7.51‐7.42 (m, 3H), 7.43 (d, *J* = 5.1, 1H), 7.19 (d, *J* = 5.1, 1H), 3.44 (t, *J* = 7.3, 2H), 1.62 (quint, *J* = 7.3, 2H), 1.39 (sextuplet, *J* = 7.3, 2H), 0.92 (t, *J* = 7.3, 3H); ^13^C NMR (125 MHz, CDCl_3_): *δ* = 153.8, 146.5, 142.3, 134.8, 129.84, 129.76, 129.6, 129.1, 128.1, 125.8, 65.7, 45.9, 32.9, 20.7, 14.0; GC‐MS: *m/z* = 409 (M^+^, 13), 392 (26), 366 (56), 353 (12), 332 (7), 282 (100), 239 (13), 211 (17), 198 (55), 171 (14), 139 (15), 105 (26); HRMS‐ESI (*m/z*): [(M+H)^+^] calcd for (C_17_H_17_INOS)^+^: 410.0070; found, 410.0069.

*(Z)‐N‐Butyl‐4‐iodo‐5‐(p‐tolyl)‐7H‐thieno[2,3‐c]pyran‐7‐imine* (**2d**). Yield: 69.5 mg, starting from 90.0 g of *N*‐butyl‐3‐(p‐tolylethynyl)thiophene‐2‐carboxamide **1d** (54%; Table 2). Yellow solid, mp = 82°C–83°C. IR (KBr): *ν* = 1667 (s), 1504 (w), 1443 (m), 1373 (w), 1250 (w), 1080 (m), 1049 (m), 1018 (m), 895 (w), 818 (w), 748 (m) cm^−1^; ^1^H NMR (500 MHz, CDCl_3_): *δ* = 7.61‐7.56 (m, 2H), 7.41 (d, *J* = 5.1, 1H), 7.29‐7.24 (m, 2H), 7.18 (dist d, *J* = 5.1, 1H), 3.43 (t, *J* = 7.3, 2H), 2.42 (s, 3H), 1.62 (quint, *J* = 7.3, 2H), 1.39 (sextuplet, *J* = 7.3, 2H), 0.92 (t, *J* = 7.3, 3H); ^13^C NMR (125 MHz, CDCl_3_): *δ* = 153.9, 146.7, 142.4, 140.0, 131.9, 129.7, 129.5, 129.0, 128.8, 125.6, 65.4, 46.0, 32.9, 21.5, 20.7, 14.0; GC‐MS: *m/z* = 423 (M^+^, 13), 406 (27), 380 (47), 367 (12), 296 (100), 253 (16), 225 (18), 212 (48), 196 (11), 119 (35); HRMS‐ESI (*m/z*): [(M+H)^+^] calcd for (C_18_H_19_INOS)^+^: 424.0227; found, 424.0211.

*(Z)‐N‐Butyl‐5‐(4‐chlorophenyl)‐4‐iodo‐7H‐thieno[2,3‐c]pyran‐7‐imine* (**2e**). Yield: 86.4 mg, starting from 96.1 mg of *N*‐butyl‐3‐((4‐chlorophenyl)ethynyl)thiophene‐2‐carboxamide **1e** (64%; Table 2). Yellow solid, mp = 95°C–96°C. IR (KBr): *ν* = 1667 (s), 1605 (w), 1489 (m), 1443 (w), 1358 (w), 1258 (m), 1088 (m), 1049 (m), 1018 (m), 895 (w), 826 (m), 802 (m), 748 (m) cm^−1^; ^1^H NMR (500 MHz, CDCl_3_): *δ* = 7.59‐7.53 (m, 2H), 7.38‐7.32 (m, 3H), 7.10 (d, *J* = 5.2, 1H), 3.34 (t, *J* = 7.3, 2H), 1.54 (quint, *J* = 7.3, 2H), 1.31 (sextuplet, *J* = 7.3, 2H), 0.84 (t, *J* = 7.3, 3H); ^13^C NMR (125 MHz, CDCl_3_): *δ* = 152.6, 146.2, 142.0, 135.8, 133.1, 130.9, 129.7, 129.2, 128.4, 126.0, 66.0, 46.0, 32.8, 20.7, 14.0; GC‐MS: *m/z* = 445 [(M+2)^+^, 4], 443 (M^+^, 10), 427 (10), 425 (23), 401 (22), 399 (52), 386 (13), 365 (8), 318 (38), 316 (100), 245 (18), 232 (52), 196 (27), 139 (29); HRMS‐ESI (*m/z*): [(M+H)^+^] calcd for (C_17_H_16_ClINOS)^+^: 443.9680; found, 443.9671.

*(Z)‐N‐Butyl‐4‐iodo‐5‐(thiophen‐3‐yl)‐7H‐thieno[2,3‐c]pyran‐7‐imine* (**2f**). Purity was ca. 90%, by ^1^H NMR. Yield: 93.3 mg, starting from 87.5 mg of *N*‐butyl‐3‐(thiophen‐3‐ylethynyl)thiophene‐2‐carboxamide **1f** (67%; Table 2). Yellow solid, mp = 52°C–53°C. IR (KBr): *ν* = 1667 (s), 1581 (m), 1442 (m), 1219 (w), 1114 (m), 1079 (m), 1055 (m), 952 (m), 790 (m) cm^−1^; ^1^H NMR (500 MHz, CDCl_3_): *δ* = 8.02‐7.97 (m, 1H), 7.63 (d, *J* = 4.8, 1H), 7.43 (dist d, *J* = 5.1, 1H), 7.39 (dist dd, *J* = 4.8, 3.0, 1H), 7.20 (d, *J* = 5.1, 1H), 3.49 (t, *J* = 7.3, 2H), 1.65 (quint, *J* = 7.3, 2H), 1.42 (sextuplet, *J* = 7.3, 2H), 0.94 (t, *J* = 7.3, 3H); ^13^C NMR (125 MHz, CDCl_3_): *δ* = 149.4, 146.5, 142.7, 135.0, 129.94, 129.89, 129.3, 128.04, 127.96, 125.2, 65.0, 46.0, 32.8, 20.8, 14.0; GC‐MS: *m/z* = 415 (M^+^, 22), 398 (35), 372 (74), 331 (33), 288 (100), 245 (30), 217 (43), 204 (92), 177 (31), 160 (39), 145 (47), 111 (87); HRMS‐ESI (*m/z*): [(M+H)^+^] calcd for (C_15_H_15_INOS_2_)^+^: 415.9634; found, 415.9636.

*(Z)‐N‐Butyl‐5‐(cyclohex‐1‐en‐1‐yl)‐4‐iodo‐7H‐thieno[2,3‐c]pyran‐7‐imine* (**2g**). Yield: 92.1 mg, starting from 87.4 mg of *N*‐butyl‐3‐(cyclohex‐1‐en‐1‐ylethynyl)thiophene‐2‐carboxamide **1** **g** (73%; Table 2). Yellow oil. IR (film): *ν* = 1652 (s), 1516 (m), 1404 (w), 1261 (m), 1077 (m), 772 (s) cm^−1^; ^1^H NMR (500 MHz, CDCl_3_): *δ* = 7.40 (d, *J* = 5.1, 1H), 7.12 (d, *J* = 5.1, 1H), 6.19 (s, br, 1H), 3.43 (t, *J* = 7.3, 2H), 2.34‐2‐26 (m, 2H), 2.26‐2.19 (m, 2H), 1.80‐1.72 (m, 2H), 1.72‐1.65 (m, 2H), 1.62 (quint, *J* = 7.3, 2H), 1.41 (sextuplet, *J* = 7.3, 2H), 0.94 (t, *J* = 7.3, 3H); ^13^C NMR (125 MHz, CDCl_3_): *δ* = 156.3, 147.1, 142.5, 134.5, 132.7, 129.8, 129.0, 125.0, 64.1, 45.8, 32.8, 26.3, 25.0, 22.3, 21.6, 20.7, 14.0; GC‐MS: *m/z* = 413 (M^+^, 15), 396 (14), 370 (50), 332 (10), 286 (100), 258 (9), 215 (23), 109 (25); HRMS‐ESI (*m/z*): [(M+H)^+^] calcd for (C_17_H_21_INOS)^+^: 414.0383; found, 414.0374.

*(Z)‐N‐Benzyl‐5‐butyl‐4‐iodo‐7H‐thieno[2,3‐c]pyran‐7‐imine* (**2h**). Yield: 62.0 mg, starting from 90.0 mg of *N*‐benzyl‐3‐(hex‐1‐yn‐1‐yl)thiophene‐2‐carboxamide **1h** (48%; Table 2). Yellow oil. IR (film): *ν* = 1656 (s), 1592 (m), 1439 (m), 1385 (w), 1351 (w), 1096 (m), 1013 (m), 754 (s) cm^−1^; ^1^H NMR (500 MHz, CDCl_3_): *δ* = 7.45‐7.38 (m, 3H), 7.35‐7.29 (m, 2H), 7.22 (dist t, *J* = 7.1, 1H), 7.07 (dist d, *J* = 5.1, 1H), 4.67 (s, 2H), 7.76 (t, *J* = 7.4, 2H), 1.70‐1.57 (m, 2H), 1.46‐1.35 (m, 2H), 0.94 (t, *J* = 7.3, 3H); ^13^C NMR (125 MHz, CDCl_3_): *δ* = 157.1, 148.1, 142.3, 140.7, 135.5, 129.5, 128.8, 128.3, 127.6, 126.4, 66.1, 49.8, 35.3, 29.3, 22.1, 13.9; GC‐MS: *m/z* = 423 (M^+^, 8), 422 (27), 380 (70), 289 (21), 253 (19), 207 813), 134 (38), 91 (100); HRMS‐ESI (*m/z*): [(M+H)^+^] calcd for (C_18_H_19_INOS)^+^: 424.0227; found, 424.0235.

*(Z)‐N‐Benzyl‐4‐iodo‐5‐phenyl‐7H‐thieno[2,3‐c]pyran‐7‐imine* (**2i**). Yield: 65.5 mg, starting from 95.0 mg of *N*‐benzyl‐3‐(phenyethynyl)thiophene‐2‐carboxamide **1i** (49%; Table 2). Yellow oil. IR (film): *ν* = 1663 (s), 1583 (w), 1492 (m), 1351 (w), 1082 (m), 1029 (w), 771 (m), 748 (m), 696 (m) cm^−1^; ^1^H NMR (500 MHz, CDCl_3_): *δ* = 7.66‐7.59 (m, 2H), 7.50‐7.42 (m, 4H), 7.41‐7.36 (m, 2H), 7.33‐7.27 (m, 2H), 7.26‐7.18 (m, 2H), 4.66 (s, 2H); ^13^C NMR (125 MHz, CDCl_3_): *δ* = 153.8, 147.7, 142.7, 140.6, 134.6, 129.9, 129.8, 129.7, 128.3, 128.1, 127.7, 126.5, 125.6, 124.7, 66.1, 49.9; GC‐MS: *m/z* = 443 (M^+^, 13), 186 (5), 139 (7), 105 (31), 91 (100); HRMS‐ESI (*m/z*): [(M+H)^+^] calcd for (C_20_H_15_INOS)^+^: 443.9914; found, 443.9923.

*(Z)‐N‐(tert‐Butyl)‐5‐butyl‐4‐iodo‐7H‐thieno[2,3‐c]pyran‐7‐imine* (**2j**). Yield: 88.2 mg, starting from 79.3 mg of *N*‐(*tert*‐butyl)‐3‐(hex‐1‐yn‐1‐yl)thiophene‐2‐carboxamide **1j** (75%; Table 2). Yellow oil. IR (film): *ν* = 1667 (s), 1560 (m), 1458 (s), 1438 (m), 1365 (w), 1204 (w), 1094 (s), 1045 (m), 933 (w), 771 (s), 748 (m) cm^−1^; ^1^H NMR (500 MHz, CDCl_3_): *δ* = 7.34 (dist d, *J* = 7.2, 1H), 7.02 (dist d, *J* = 5.2, 1H), 2.76 (t, *J* = 7.6, 2H), 1.73‐1.64 (m, 2H), 1.50‐1.39 (m, 2H), 1.37 (s, 9H), 0.97 (t, *J* = 7.3, 3H); ^13^C NMR (125 MHz, CDCl_3_): *δ* = 157.2, 144.0, 141.5, 128.9, 128.8, 126.3, 65.9, 53.5, 35.5, 29.9, 29.6, 22.1, 13.9; GC‐MS: *m/z* = 389 (M^+^, 19), 333 (82), 291 (11), 206 (42), 189 (20), 164 (51), 136 (48), 122 (25), 57 (100); HRMS‐ESI (*m/z*): [(M+H)^+^] calcd for (C_15_H_21_INOS)^+^: 390.0383; found, 390.0385.

*(Z)‐N‐(tert‐Butyl)‐4‐iodo‐5‐phenyl‐7H‐thieno[2,3‐c]pyran‐7‐imine* (**2k**). Yield: 108.1 mg, starting from 85.4 mg of *N*‐(*tert*‐butyl)‐3‐(phenylethynyl)thiophene‐2‐carboxamide **1k** (88%; Table 2). Yellow solid, mp = 95°C–96°C. IR (KBr): *ν* = 1671 (s), 1587 (m), 1444 (m), 1360 (m), 1211 (m), 1078 (m), 990 (m), 887 (w), 745 (m) cm^−1^; ^1^H NMR (500 MHz, CDCl_3_): *δ* = 7.68‐7.61 (m, 2H), 7.50‐7.41 (m, 3H), 7.39 (d, *J* = 5.1, 1H), 7.16 (d, *J* = 5.1, 1H), 1.33 (s, 9H); ^13^C NMR (125 MHz, CDCl_3_): *δ* = 154.4, 143.2, 141.8, 135.1, 129.67, 129.65, 129.4, 128.9, 128.1, 127.3, 66.0, 53.6, 29.9; GC‐MS: *m/z* = 409 (M^+^, 12), 353 (100), 226 (12), 198 (57), 171 (15), 139 (17), 57 (55); HRMS‐ESI (*m/z*): [(M+H)^+^] calcd for (C_17_H_17_INOS)^+^: 410.0070; found, 410.0068.

*(Z)‐5‐Butyl‐4‐iodo‐N‐phenyl‐7H‐thieno[2,3‐c]pyran‐7‐imine* (**2l**). Yield: 108.0 mg, starting from 85.6 mg of 3‐(hex‐1‐yn‐1‐yl)‐*N*‐phenylthiophene‐2‐carboxamide **1l** (87%; Table 2). Yellow oil. IR (film): *ν* = 1651 (s), 1589 (m), 1481 (w), 1443 (m), 1258 (m), 1096 (s), 1018 (s), 802 (m) cm^−1^; ^1^H NMR (500 MHz, CDCl_3_): *δ* = 7.44 (d, *J* = 5.1, 1H), 7.27‐7.21 (m, 2H), 7.15‐7.10 (m, 2H), 7.04 (d, *J* = 5.1, 1H), 7.02‐6.97 (m, 1H), 2.63 (t, *J* = 7.4, 2H), 1.49 (quint, *J* = 7.4, 2H), 1.27 (sextuplet, *J* = 7.4, 2H), 0.82 (t, *J* = 7.4, 3H); ^13^C NMR (125 MHz, CDCl_3_): *δ* = 157.2, 146.6, 145.6, 143.6, 131.0, 129.0, 128.6, 124.8, 123.7, 123.2, 66.7, 35.0, 29.4, 22.0, 13.8; GC‐MS: *m/z* = 409 (M^+^, 100), 282 (35), 241 (11), 226 (22), 212 (35); HRMS‐ESI (*m/z*): [(M+H)^+^] calcd for (C_17_H_17_INOS)^+^: 410.0070; found, 410.0076.

*(Z)‐5‐(tert‐Butyl‐4‐iodo‐N‐phenyl‐7H‐thieno[2,3‐c]pyran‐7‐imine* (**2m**). Yield: 108.5 mg, starting from 85.1 mg of 3‐(3,3‐dimethylbut‐1‐yn‐1‐yl)‐*N*‐phenylthiophene‐2‐carboxamide **1m** (88%; Table 2). Yellow solid, mp = 90°C–91°C. IR (KBr): ν = 1660 (s), 1593 (m), 1557 (w), 1485 (w), 1438 (w), 1094 (m), 1043 (m), 1010 (w), 772 (m) cm^−1^; ^1^H NMR (500 MHz, CDCl_3_): δ = 7.51 (d, *J* = 5.2, 1H), 7.33‐7.26 (m, 3H), 7.10‐7.02 (m, 3H), 1.34 (s, 9H); ^13^C NMR (125 MHz, CDCl_3_): δ = 161.0, 146.6, 146.2, 145.5, 130.3, 129.5, 128.5, 124.1, 123.4, 122.6, 63.4, 38.9, 29.1; GC‐MS: *m/z* = 409 (M^+^, 100), 352 (41), 226 (70), 196 (10); HRMS‐ESI (*m/z*): [(M+H)^+^] calcd for (C_17_H_17_INOS)^+^: 410.0070; found, 410.0059.

*(Z)‐4‐Iodo‐N,5‐diphenyl‐7H‐thieno[2,3‐c]pyran‐7‐imine* (**2n**). Yield: 101.5 mg, starting from 91.6 mg of *N*‐phenyl‐3‐(phenylethynyl)thiophene‐2‐carboxamide **1n** (78%; Table 2). Yellow solid, mp = 148°C–150 °C. IR (KBr): *ν* = 1654 (s), 1591 (m), 1488 (w), 1444 (w), 1083 (m), 1045 (w), 900 (w), 764 (m), 693 (m) cm^−1^; ^1^H NMR (500 MHz, CDCl_3_): *δ* = 7.80‐7.47 (m, 3H), 7.47‐7.15 (m, 8H), 7.05‐6.95 (m, 1H); ^13^C NMR (125 MHz, CDCl_3_): *δ* = 153.7, 145.9, 145.2, 144.0, 134.0, 131.0, 130.0, 129.7, 129.6, 128.0, 126.0, 123.9, 123.3, 121.8, 66.5; GC‐MS: *m/z* = 429 (M^+^, 100), 401 (8), 302 (25), 273 (36), 241 (9), 196 (15), 171 (16), 139 (22), 77 (48); HRMS‐ESI (*m/z*): [(M+H)^+^] calcd for (C_19_H_13_INOS)^+^: 429.9757; found, 429.9741.

*(Z)‐4‐Iodo‐N‐phenyl‐5‐(p‐tolyl)‐7H‐thieno[2,3‐c]pyran‐7‐imine* (**2o**). Yield: 107.9 mg, starting from 96.2 mg of *N*‐phenyl‐3‐(p‐tolylethynyl)thiophene‐2‐carboxamide **1o** (80%; Table 2). Yellow solid, mp = 109°C–111°C. IR (KBr): *ν* = 1668 (s), 1582 (s), 1506 (m), 1485 (m), 1440 (m), 1216 (m), 1082 (s), 1044 (s), 901 (m), 770 (s) cm^−1^; ^1^H NMR (500 MHz, CDCl_3_): *δ* = 7.58‐7.50 (m, 3H), 7.30‐7.22 (m, 5H), 7.19 (d, *J* = 7.7, 2H), 7.03 (t, *J* = 6.3, 1H), 2.38 (s, 3H); ^13^C NMR (125 MHz, CDCl_3_): *δ* = 153.9, 146.1, 145.3, 144.1, 140.2, 131.2, 130.9, 130.0, 129.6, 128.7, 128.6, 123.8, 123.3, 66.0, 21.5; GC‐MS: *m/z* = 443 (M^+^, 100), 316 (23), 288 (42), 273 (25), 153 (21), 77 (24); HRMS‐ESI (*m/z*): [(M+H)^+^] calcd for (C_20_H_15_INOS)^+^: 443.9914; found, 443.9908.

*(Z)‐5‐(4‐chlorophenyl)‐4‐Iodo‐N‐phenyl‐7H‐thieno[2,3‐c]pyran‐7‐imine* (**2p**). Yield: 115.4 mg, starting from 101.0 mg of 3‐((4‐chlorophenyl)ethynyl)‐*N*‐phenylthiophene‐2‐carboxamide **1p** (83%; Table 2). Yellow solid, mp = 146°C–147°C. IR (KBr): *ν* = 1653 (s), 1592 (s), 1507 (m), 1488 (w), 1091 (m), 1044 (m), 1014 (m), 900 (w), 834 (w), 751 (m) cm^−1^; ^1^H NMR (500 MHz, CDCl_3_): *δ* = 7.58‐7.52 (m, 3H), 7.36‐7.32 (m, 2H), 7.30‐7.24 (m, 2H), 7.24‐7.18 (m, 3H), 7.04 (t, *J* = 7.3, 1H); ^13^C NMR (125 MHz, CDCl_3_): *δ* = 152.5, 145.7, 145.2, 143.8, 136.0, 132.4, 131.2, 131.0, 130.0, 128.6, 128.4, 124.0, 123.2, 66.8; GC‐MS: *m/z* = 465 [(M+2)^+^, 40], 464 (26), 463 (M^+^, 100), 434 (15), 336 (16), 308 (30), 301 (30), 273 (29), 272 (20), 205 (11), 196 (12), 173 (13), 136 (14), 111 (7), 77 (18); HRMS‐ESI (*m/z*): [(M+H)^+^] calcd for (C_19_H_12_ClINOS)^+^: 463.9367; found, 463.9356.

*(Z)‐5‐(Cyclohex‐1‐en‐1‐yl)‐4‐Iodo‐N‐phenyl‐7H‐thieno[2,3‐c]pyran‐7‐imine* (**2q**). Yield: 124.0 mg, starting from 92.7 mg of 3‐(cyclohex‐1‐en‐1‐ylethynyl)‐*N*‐phenylthiophene‐2‐carboxamide **1q** (95%; Table 2). Yellow oil. IR (film): *ν* = 1652 (s), 1592 (m), 1533 (w), 1442 (m), 1219 (w), 1042 (w), 771 (s) cm^−1^; ^1^H NMR (500 MHz, CDCl_3_): *δ* = 7.51 (d, *J* = 5.2, 1H), 7.34‐7.27 (m, 2H), 7.24‐7.20 (m, 2H), 7.20‐7.16 (m, 1H), 7.06 (t, *J* = 7.3, 1H), 6.20‐6.16 (m, 1H), 2.20‐2.12 (m, 4H), 1.72‐1.64 (m, 2H), 1.64‐1.57 (m, 2H); ^13^C NMR (125 MHz, CDCl_3_): *δ* = 156.2, 146.3, 145.5, 144.1, 135.2, 132.2, 130.8, 128.5, 123.7, 123.3, 64.7, 26.5, 25.0, 22.2, 21.5; GC‐MS: *m/z* = 433 (M^+^, 100), 404 (13), 306 (9), 278 (14), 250 (11), 236 (9), 185 (9), 115 (8), 77 (34); HRMS‐ESI (*m/z*): [(M+H)^+^] calcd for (C_19_H_17_INOS)^+^: 434.0070; found, 434.0088.

*(Z)‐5‐Butyl‐N‐(4‐(tert‐butyl)phenyl)‐4‐iodo‐N‐phenyl‐7H‐thieno[2,3‐c]pyran‐7‐imine* (**2r**). Yield: 107.5 mg, starting from 102.2 mg of *N*‐(4‐*tert*‐butyl)phenyl)‐3‐(hex‐1‐yn‐1‐yl)thiophene‐2‐carboxamide **1r** (77%; Table 2). Yellow oil. IR (film): *ν* = 1669 (s), 1587 (w), 1507 (w), 1489 (w), 1360 (w), 1211 (m), 1075 (m), 1049 (m), 990 (m), 887 (w), 746 (m) cm^−1^; ^1^H NMR (500 MHz, CDCl_3_): *δ* = 7.50 (d, *J* = 5.1, 1H), 7.39‐7.29 (m, 2H), 7.23‐7.16 (m, 2H), 7.11 (dist d, *J* = 5.1, 1H), 2.74 (t, *J* = 7.3, 2H), 1.61 (quint, *J* = 7.3, 2H), 1.42‐1.30 (m, 11H), 0.92 (t, *J* = 7.3, 3H); ^13^C NMR (125 MHz, CDCl_3_): δ = 157.2, 146.7, 146.3, 143.3, 142.6, 130.7, 128.9, 125.4, 125.2, 123.0, 66.7, 35.0, 31.5, 31.4, 29.4, 22.0, 13.8; GC‐MS: *m/z* = 465 (M^+^, 68), 451 (24), 450 (100), 91 (11); HRMS‐ESI (*m/z*): [(M+H)^+^] calcd for (C_21_H_25_INOS)^+^: 466.0696; found, 466.0682.

*(Z)‐N‐(4‐(tert‐butyl)phenyl)‐4‐iodo‐5‐phenyl‐7H‐thieno[2,3‐c]pyran‐7‐imine* (**2s**). Yield: 115.8 mg, starting from 108.2 mg of *N*‐(4‐*tert*‐butyl)phenyl)‐3‐(phenylethynyl)thiophene‐2‐carboxamide **1s** (79%; Table 2). Yellow solid, mp = 149°C–150°C. IR (KBr): *ν* = 1653 (s), 1601 (w), 1508 (m), 1081 (m), 1045 (w), 841 (w), 751 (m) cm^−1^; ^1^H NMR (500 MHz, CDCl_3_): *δ* = 7.71‐7.61 (m, 2H), 7.53 (d, *J* = 5.2, 1H), 7.45‐7.35 (m, 3H), 7.31‐7.20 (m, 5H), 1.29 (s, 9H); ^13^C NMR (125 MHz, CDCl_3_): *δ* = 153.8, 146.9, 145.4, 143.6, 142.3, 134.2, 130.7, 129.9, 129.8, 128.0, 126.4, 126.3, 125.4, 123.3, 66.5, 34.3, 31.4; GC‐MS: *m/z* = 485 (M^+^, 55), 470 (100), 328 (15), 211 (12), 171 (12), 139 (40), 77 (57); HRMS‐ESI (*m/z*): [(M+H)^+^] calcd for (C_23_H_21_INOS)^+^: 486.0383; found, 486.0399.

### Representative Iodocyclization Procedure on a Larger Scale

To a solution of **1a** (315.5 mg, 1.20 mmol) in ChCl/urea (1/2 molar ratio; 8.0 mL) was added I_2_ (610.0 mg, 2.4 mmol). The mixture was allowed to stir at 75°C (oil bath) for 18 h in a sealed tube and then cooled to room temperature. Et_2_O (3 mL) was added, the mixture heated to 60 °C, cooled to room temperature, and phases were separated. This extraction procedure was repeated for additional nine times. Satd. aqueous Na_2_S_2_O_3_ (25 mL) was added to the collected ethereal phases, and the mixture was allowed to stir for 10 min. Phases were separated, and the aqueous phase was extracted with Et_2_O (3 × 20 mL). The collected organic layers were dried over Na_2_SO_4_. After filtration and evaporation of the solvent, product **2a** was purified by column chromatography on alumina using as eluent pure hexane to 98:2 hexane‒AcOEt (yield: 383 mg, 82%).

### Sonogashira Coupling Leading to 4‐Alkynyl‐7*H*‐Thieno[2,3‐*c*]Pyran‐7‐Imines 4 in DES

To a solution of **2** (0.25 mmol; **2a**, 98.0 mg; **2l**, 103.0 mg; **2** **m**, 102.8 mg; **2n**, 107.5 mg) in ChCl/Gly (1/2 molar ratio; 1.4 mL) were added PdCl_2_(PPh_3_)_2_ (17.5 mg, 0.025 mmol), CuI (9.5 mg, 0.05 mmol), the terminal alkyne (0.5 mmol; ethynylbenzene, 51.5 mg; 3‐ethynylthiophene, 54.3 mg; 1‐hexyne, 41.2 mg; 1‐ethynylcyclohex‐1‐ene, 53.2 mg; 1‐ethynyl‐4‐methylbenzene, 58.0 mg), and anhydrous Et_3_N (76 mg, 0.75 mmol) (Table 4). The mixture was allowed to stir under nitrogen at 60°C (oil bath) in a sealed tube for 15 h and then cooled to room temperature. Et_2_O (5 mL) was added, the mixture was heated to 60°C, cooled to room temperature, and phases were separated. This extraction procedure was repeated for additional five times. The DES phase was used again for the recycling experiments (see below). After evaporation of the solvent from the collected ethereal phases, products **4** were purified by column chromatography on alumina using as eluent pure hexane to 98:2 hexane‒AcOEt (isolated yields are shown in Table 4).

### Recycling Procedure

To the DES residue obtained as described above, still containing the catalysts PdCl_2_(PPh_3_)_2_ and CuI, was added a solution of **2** (0.25 mmol) in Et_2_O (1.5 mL). The Et_2_O was removed under vacuum and the terminal alkyne (0.5 mmol) followed by anhydrous Et_3_N (76.0 mg, 0.75 mmol) were added. Then the same procedure described above was followed.

*(Z)‐N,5‐Dibutyl‐4‐(phenylethynyl)‐7H‐thieno[2,3‐c]pyran‐7‐imine* (**4aa**). Yield: 65.3 mg, starting from 98.0 mg of *(Z)‐N*,5‐dibutyl‐4‐iodo‐7*H‐*thieno[2,3‐*c*]pyran‐7‐imine **2a** (71%; Table 4). Yellow oil. IR (film): *ν* = 2214 (vw), 1671 (s), 1542 (w), 1444 (m), 1259 (m), 1134 (w), 1025 (w), 757 (m), 692 (m) cm^−1^; ^1^H NMR (500 MHz, CDCl_3_): *δ* = 7.53‐7.47 (m, 2H), 7.44 (d, *J* = 5.0, 1H), 7.39‐7.31 (m, 3H), 7.21 (dist d, *J* = 5.0, 1H), 3.49 (t, *J* = 7.3, 2H), 2.28 (t, *J* = 7.4, 2H), 1.74 (quit, *J* = 7.4, 2H), 1.65 (quint, *J* = 7.3, 2 H), 1.51‐1.39 (m, 4H), 1.02‐0.92 (m, 6H); ^13^C NMR (125 MHz, CDCl_3_): *δ* = 162.5, 146.6, 139.5, 131.3, 130.3, 128.4, 128.3, 124.2, 124.1, 123.3, 96.8, 94.8, 82.8, 45.9, 32.8, 31.7, 29.3, 22.1, 20.8, 14.0, 13.8; GC‐MS: *m/z* = 363 (M^+^, 31), 334 (46), 320 (100), 306 (61), 278 (16), 264 (55), 222 (22); HRMS‐ESI (*m/z*): [(M+H)^+^] calcd for (C_23_H_26_NOS)^+^: 364.1730; found, 364.1728.

*(Z)‐5‐Butyl‐N‐phenyl‐4‐(phenylethynyl)‐7H‐thieno[2,3‐c]pyran‐7‐imine* (**4la**). Yield: 80.4 mg, starting from 103.0 mg of *(Z)‐*5‐Butyl‐4‐iodo‐*N*‐phenyl‐7*H*‐thieno[2,3‐*c*]pyran‐7‐imine **2l** (83%; Table 4). Yellow oil. IR (film): *ν* = 2208 (vw), 1660 (s), 1592 (m), 1487 (w), 1143 (w), 1103 (w), 1005 (m), 755 (m), 718 (m), 691 (m) cm^−1^; ^1^H NMR (500 MHz, CDCl_3_): *δ* = 7.63 (d, *J* = 5.2, 1H), 7.58‐7.51 (m, 2H), 7.45‐7.35 (m, 5H), 7.32 (d, *J* = 5.2, 1H), 7.11 (td, *J* = 7.3, 0.8, 1H), 2.78 (t, *J* = 7.3, 2H), 1.66 (quint, *J* = 7.3, 2 H), 1.41 (sextuplet, *J* = 7.3, 2H), 0.95 (t, *J* = 7.3, 3H); ^13^C NMR (125 MHz, CDCl_3_): *δ* = 162.5, 146.2, 145.6, 141.2, 134.2, 132.3, 131.3, 129.5, 128.6, 128.5, 124.3, 123.7, 123.2, 122.0, 97.9, 95.3, 82.2, 31.5, 29.4, 22.0, 13.8; GC‐MS: *m/z* = 383 (M^+^, 95), 340 (80), 299 (100), 254 (8), 220 (12), 151 (19), 105 (40), 77 (48); HRMS‐ESI (*m/z*): [(M+H)^+^] calcd for (C_25_H_22_NOS)^+^: 384.1417; found, 348.1412.

*(Z)‐5‐Butyl‐N‐phenyl‐4‐(thiophen‐3‐ylethynyl)‐7H‐thieno[2,3‐c]pyran‐7‐imine* (**4lb**). Yield: 44.5 mg, starting from 103.3 mg of *(Z)‐*5‐Butyl‐4‐iodo‐*N*‐phenyl‐7*H*‐thieno[2,3‐*c*]pyran‐7‐imine **2l** (45%; Table 4). Yellow oil. IR (film): *ν* = 2192 (vw), 1656 (s), 1592 (m), 1487 (w), 1143 (w), 1244 (w), 1124 (w), 757 (m) cm^−1^; ^1^H NMR (500 MHz, CDCl_3_): *δ* = 7.58 (d, *J* = 5.1, 1H), 7.53‐7.48 (m, 1H), 7.38‐7.31 (m, 3H), 7.29‐7.22 (m, 3H), 7.22‐7.16 (m, 1H), 7.13‐7.06 (m, 1H), 2.74 (t, *J* = 7.3, 2H), 1.63 (quint, *J* = 7.3, 2H), 1.38 (sextuplet, *J* = 7.3, 2H), 0.92 (t, *J* = 7.3, 3H); ^13^C NMR (125 MHz, CDCl_3_): *δ* = 162.5, 146.3, 145.6, 141.3, 134.3, 132.3, 129.7, 128.6, 128.5, 125.6, 124.3, 123.8, 123.3, 122.0, 97.8, 90.4, 81.7, 31.5, 29.3, 22.0, 13.8; GC‐MS: *m/z* = 389 (M^+^, 71), 346 (76), 332 (61), 305 (100), 284 (12), 260 (22), 226 811), 201 (16), 183 (14), 157 (19), 11 (27); HRMS‐ESI (*m/z*): [(M+H)^+^] calcd for (C_23_H_20_NOS_2_)^+^: 390.0981; found, 390.0974.

*(Z)‐5‐Butyl‐4‐(hex‐1‐yn‐1‐yl)‐N‐phenyl‐7H‐thieno[2,3‐c]pyran‐7‐imine* (**4lc**). Yield: 46.0 mg, starting from 103.0 mg of *(Z)‐*5‐Butyl‐4‐iodo‐*N*‐phenyl‐7*H*‐thieno[2,3‐*c*]pyran‐7‐imine **2l** (50%; Table 4). Yellow oil. IR (film): *ν* = 2240 (vw), 1660 (s), 1599 (m), 1538 (w), 1444 (w), 1143 (w), 1244 (w), 1124 (w), 757 (m) cm^−1^; ^1^H NMR (500 MHz, CDCl_3_): *δ* = 7.55 (d, *J* = 5.1, 1H), 7.36‐7.30 (m, 3H), 7.25‐7.20 (m, 2H), 7.18 (dist d, *J* = 5.1, 1H), 7.10‐7.04 (m, 1H), 2.66 (t, *J* = 7.3, 2H), 2.46 (t, *J* = 7.3, 2H), 1.67‐1.56 (m, 4H), 1.54‐1.45 (m, 2H), 1.35 (sextuplet, *J* = 7.3, 2H), 0.96 (t, *J* = 7.3, 3H), 0.91 (t, *J* = 7.3, 3H); ^13^C NMR (125 MHz, CDCl_3_): *δ* = 161.8, 146.6, 145.8, 142.0, 132.1, 128.5, 124.4, 123.6, 123.3, 122.1, 98.2, 96.3, 73.2, 31.2, 30.9, 29.5, 29.4, 22.0, 19.4, 19.3, 13.8; GC‐MS: *m/z* = 363 (M^+^, 100), 334 (34), 321 (89), 278 (51), 150 (24), 236 (46), 222 (15), 114 (8), 77 (50); HRMS‐ESI (*m/z*): [(M+H)^+^] calcd for (C_23_H_26_NOS)^+^: 364.1730; found, 364.1729.

*(Z)‐5‐(tert‐Butyl)‐N‐phenyl‐4‐(phenylethynyl)‐7H‐thieno[2,3‐c]pyran‐7‐imine* (**4ma**). Yield: 72.5 mg, starting from 102.8 mg of *(Z)‐*5‐(*tert‐*Butyl‐4‐iodo‐*N‐*phenyl‐7*H‐*thieno[2,3*‐c*]pyran‐7‐imine **2** **m** (75%; Table 4). Yellow oil. IR (film): *ν* = 2203 (vw), 1663 (s), 1592 (m), 1529 (m), 1486 (w), 1443 (w), 1254 (w), 1156 (m), 1117 (m), 1039 (m), 1005 (w), 755 (s) cm^−1^; ^1^H NMR (500 MHz, CDCl_3_): *δ* = 7.59 (d, *J* = 5.0, 1H), 7.54‐7.47 (m, 2H), 7.44‐7.29 (m, 7H), 7.15 (dist d, *J* = 7.6, 1H), 7.08 (t, *J* = 7.4, 1H), 1.37 (s, 9H); ^13^C NMR (125 MHz, CDCl_3_): *δ* = 167.8, 146.2, 146.1, 142.8, 132.1, 130.9, 129.4, 129.1, 128.5, 124.7, 124.5, 123.5, 123.3, 122.8, 97.9, 96.1, 83.3, 38.5, 28.9; GC‐MS: *m/z* = 383 (M^+^, 49), 368 (100), 326 (57), 298 (58), 220 (9), 195 (10), 151 (13), 105 (14), 77 (27); HRMS‐ESI (*m/z*): [(M+H)^+^] calcd for (C_25_H_22_NOS)^+^: 384.1417; found, 384.1412.

*(Z)‐5‐(tert‐Butyl)‐4‐(cyclohex‐1‐en‐1‐ylethynyl)‐N‐phenyl‐7H‐thieno[2,3‐c]pyran‐7‐imine* (**4md**). Yield: 47.0 mg, starting from 102.8 mg of *(Z)‐*5‐(*tert‐*Butyl‐4‐iodo‐*N‐*phenyl‐7*H‐*thieno[2,3*‐c*]pyran‐7‐imine **2** **m** (48%; Table 4). Yellow oil. IR (film): *ν* = 2183 (w), 1667 (s), 1593 (m), 1443 (w), 1216 (w), 1150 (w), 1039 (w), 757 (s) cm^−1^; ^1^H NMR (500 MHz, CDCl_3_): *δ* = 7.58‐7.50 (m, 1H), 7.40‐7.21 (m, 4H), 7.16‐7.00 (m, 3H), 6.18 (s, br, 1H), 2.26‐2.20 (m, 1H), 2.20‐2.12 (m, 3H), 1.74‐1.66 (m, 2H), 1.66‐1.59 (m, 2H), 1.31 (s, 9H); ^13^C NMR (125 MHz, CDCl_3_): *δ* = 166.9, 146.6, 146.1, 143.2, 134.6, 131.9, 128.5, 124.8, 124.3, 123.4, 122.9, 120.9, 99.9, 96.5, 80.5, 38.3, 30.9, 28.8, 25.7, 22.2, 21.5; GC‐MS: *m/z* = 387 (M^+^, 63), 372 (14), 330 (100), 302 (57), 274 (16), 209 (10), 77 (39); HRMS‐ESI (*m/z*): [(M+H)^+^] calcd for (C_25_H_26_NOS)^+^: 388.1730; found, 388.1731.

*(Z)‐N,5‐(Diphenyl)‐4‐(phenylethynyl)‐7H‐thieno[2,3‐c]pyran‐7‐imine* (**4na**). Yield: 83.2 mg, starting from 107.5 mg of (*Z*)‐4‐iodo‐*N*,5‐diphenyl‐7*H*‐thieno[2,3‐*c*]pyran‐7‐imine **2n** (82%; Table 4). Yellow solid, mp = 153°C–154°C. IR (KBr): *ν* = 2208 (vw), 1659 (s), 1591 (m), 1486 (w), 1345 (w), 1234 (w), 1132 (w), 1011 (m), 754 (m), 689 (m) cm^−1^; ^1^H NMR (500 MHz, CDCl_3_): *δ* = 8.07‐7.99 (m, 2H), 7.59 (d, *J* = 5.1, 1H), 7.48‐7.43 (m, 2H), 7.41‐7.26 (m, 11H), 7.13‐7.07 (m, 1H); ^13^C NMR (125 MHz, CDCl_3_): *δ* = 156.3, 145.6, 145.5, 142.1, 132.4, 131.3, 130.1, 128.7, 128.6, 128.5, 128.2, 127.9, 125.9, 125.0, 123.9, 123.1, 122.9, 97.2, 96.3, 83.7; GC‐MS: *m/z* = 403 (M^+^, 25), 326 (5), 298 (20), 151 (3), 105 (100), 77 (40); HRMS‐ESI (*m/z*): [(M+H)^+^] calcd for (C_27_H_18_NOS)^+^: 404.1104; found, 404.1102.

*(Z)‐N,5‐Diphenyl‐4‐(p‐tolylethynyl)‐7H‐thieno[2,3‐c]pyran‐7‐imine* (**4ne**). Yield: 76.0 mg, starting from 108.0 mg of (*Z*)‐4‐iodo‐*N*,5‐diphenyl‐7*H*‐thieno[2,3‐*c*]pyran‐7‐imine **2n** (72%; Table 4). Yellow solid, mp = 138°C–140°C. IR (KBr): *ν* = 2206 (vw), 1659 (s), 1591 (m), 1525 (m), 1486 (w), 1446 (w), 1257 (w), 1131 (w), 1030 (m), 768 (m) cm^−1^; ^1^H NMR (500 MHz, CDCl_3_): *δ* = 8.10‐7.98 (m, 2H), 7.63‐7.56 (m, 1H), 7.44‐7.30 (m, 8H), 7.30‐7.26 (m, 2H), 7.18‐7.12 (m, 2H), 7.12‐7.07 (m, 1H), 2.36 (s, 3H); ^13^C NMR (125 MHz, CDCl_3_): *δ* = 156.0, 145.7, 145.6, 142.2, 138.9, 132.3, 131.2, 130.0, 129.2, 128.7, 128.2, 127.8, 125.9, 125.1, 123.8, 123.1, 119.8, 97.4, 96.8, 83.0, 21.5; GC‐MS: *m/z* = 417 (M^+^, 100), 340 (22), 312 (93), 297 (29), 264 (5), 233 (5), 208 (7), 163 (7), 105 (88), 77 (75); HRMS‐ESI (*m/z*): [(M+H)^+^] calcd for (C_28_H_20_NOS)^+^: 418.1260; found, 418.1260.

### Representative Sonogashira Coupling Procedure on a Larger Scale

To a solution of (*Z*)‐*N*,5‐dibutyl‐4‐iodo‐7*H*‐thieno[2,3‐*c*]pyran‐7‐imine **2a** (1.0 mmol; 389 mg) in ChCl/Gly (1/2 molar ratio; 6 mL) were added PdCl_2_(PPh_3_)_2_ (70.7 mg, 0.1 mmol), CuI (38.2 mg, 0.2 mmol), 1‐hexyne (164.4 mg, 2.0 mmol), and anhydrous Et_3_N (304 mg, 3.0 mmol). The mixture was allowed to stir at 60°C (oil bath) under nitrogen in a sealed tube for 15 h and then cooled to room temperature. Et_2_O (5 mL) was added, the mixture was heated to 60°C, cooled to room temperature, and phases were separated. This extraction procedure was repeated for additional five times. After evaporation of the solvent from the collected ethereal phases, product **4aa** was purified by column chromatography on alumina using as eluent pure hexane to 95:5 hexane‒AcOEt (yield: 293 mg, 81%).

### Suzuki–Miyaura Coupling Leading to 4‐aryl‐7*H*‐Thieno[2,3‐*c*]Pyran‐7‐Imines 6 in DES

To a solution of **2** (0.25 mmol; **2l**, 102.5 mg; **2n**, 107.0 mg) in Bet/Gly (1/2, molar ratio; 1.3 mL) were added PdCl_2_ (4.5 mg, 0.025 mmol), Na_2_CO_3_ (53.0 mg, 0.5 mmol), and the boronic acid (0.375 mmol; phenylboronic acid, 45.7 mg; *p*‐tolylboronic acid, 51.0 mg; furan‐3‐ylboronic acid, 41.9 mg) (Table 5). The reaction mixture was allowed to stir at 100°C (oil bath) under nitrogen in a sealed tube for 15 h and then cooled to room temperature. Et_2_O (2 mL) was added, the mixture was heated to 60 °C, cooled to room temperature, and phases were separated. This extraction procedure was repeated for additional five times. The DES phase was used again for the recycling experiments (see below). After evaporation of the solvent from the collected ethereal phases, products **6** were purified by column chromatography on alumina using hexane to 98:2 hexane–AcOEt as the eluent (isolated yields are shown in Table 5).

### Recycling Procedure

To the DES residue obtained as described above, still containing PdCl_2_, was added a solution of **2** (0.25 mmol) in Et_2_O (1.5 mL). The Et_2_O was removed under vacuum, then Na_2_CO_3_ (53.0 mg, 0.5 mmol) and the boronic acid (0.375 mmol) were added. Then the same procedure described above was followed.

*(Z)‐5‐Butyl‐N,4‐phenyl‐7H‐thieno[2,3‐c]pyran‐7‐imine* (**6la**). Yield: 61.5 mg, starting from 102.5 mg of (*Z*)‐5‐butyl‐4‐iodo‐*N*‐phenyl‐7*H*‐thieno[2,3‐*c*]pyran‐7‐imine **2l** (68%; Table 5, Entry 4). Yellow oil. IR (film): *ν* = 1656 (s), 1590 (m), 1486 (w), 1442 (w), 1225 (w), 1100 (w), 1019 (m), 765 (m), 720 (m), 701 (m) cm^−1^; ^1^H NMR (500 MHz, CDCl_3_): *δ* = 7.50‐7.24 (m, 10H), 7.11‐7.04 (m, 1H), 2.32 (t, *J* = 7.4, 2H), 1.51 (quint, *J* = 7.4, 2H), 1.21 (sextuplet, *J* = 7.4, 2H), 0.78 (t, *J* = 7.4, 3H); ^13^C NMR (125 MHz, CDCl_3_): *δ* = 154.8, 147.8, 146.2, 143.4, 135.3, 131.8, 129.9, 129.4, 128.7, 128.5, 127.8, 125.7, 124.4, 123.4, 114.3, 29.8, 22.0, 13.7; GC‐MS: *m/z* = 359 (M^+^, 100), 302 (98), 275 (48), 241 (8), 196 (10), 171 (24), 127 (15), 77 (35); HRMS‐ESI (*m/z*): [(M+H)^+^] calcd for (C_23_H_22_NOS)^+^: 360.1417; found, 360.1413.

*(Z)‐5‐Butyl‐N‐phenyl‐4‐(p‐tolyl)‐7H‐thieno[2,3‐c]pyran‐7‐imine* (**6lb**). Yield: 60.2 mg, starting from 102.8 mg of (*Z*)‐5‐butyl‐4‐iodo‐*N*‐phenyl‐7*H*‐thieno[2,3‐*c*]pyran‐7‐imine **2l** (64%; Table 5, Entry 5). Yellow solid, mp 85°C–87°C. IR (KBr): *ν* = 1659 (s), 1620 (m), 1514 (w), 1486 (w), 1441 (w), 1224 (w), 1101 (m), 1017 (m), 857 (m), 761 (m) cm^−1^; ^1^H NMR (500 MHz, CDCl_3_): *δ* = 7.45 (dist d, *J* = 5.1, 1H), 7.36‐7.30 (m, 2H), 7.30‐7.20 (m, 4H), 7.20‐7.12 (m, 2H), 7.07 (t, *J* = 7.3, 1H), 6.68 (d, *J* = 5.1, 1H), 2.41 (s, 3H), 2.33 (t, *J* = 7.4, 2H), 1.50 (quint, *J* = 7.4, 2H), 1.22 (sextuplet, *J* = 7.4, 2H), 0.79 (t, *J* = 7.4, 3H); ^13^C NMR (125 MHz, CDCl_3_): *δ* = 154.8, 147.9, 146.3, 143.7, 137.4, 132.2, 131.7, 129.8, 129.4, 128.5, 125.6, 124.6, 124.5, 123.3, 114.2, 29.92, 29.86, 22.0, 21.3, 13.8; GC‐MS: *m/z* = 373 (M^+^, 65), 316 (100), 289 (38), 288 (31), 273 (18), 210 (5), 184 (12), 141 86), 115 (6), 77 (18); HRMS‐ESI (*m/z*): [(M+H)^+^] calcd for (C_24_H_24_NOS)^+^: 374.1573; found, 374.1575.

*(Z)‐N,4,5‐Triphenyl‐7H‐thieno[2,3‐c]pyran‐7‐imine* (**6na**). Yield: 67.5 mg, starting from 107.0 mg of (*Z*)‐4‐iodo‐*N*,5‐diphenyl‐7*H‐*thieno[2,3‐*c*]pyran‐7‐imine **2n** (71%; Table 5, Entry 6). Yellow solid, mp = 179°C–182°C. IR (KBr): *ν* = 1652 (s), 1610 (m), 1486 (w), 1444 (w), 1205 (m), 1088 (w), 801 (m), 728 (m) cm^−1^; ^1^H NMR (500 MHz, CDCl_3_): *δ* = 7.51‐7.47 (m, 1H), 7.39‐7.30 (m, 7H), 7.28‐7.23 (m, 2H), 7.20‐7.14 (m, 3H), 7.14‐7.04 (m, 3H), 6.83‐6.79 (m, 1H); ^13^C NMR (125 MHz, CDCl_3_): *δ* = 150.7, 147.0, 146.0, 143.7, 135.5, 132.7, 132.0, 130.3, 129.0, 128.8, 128.7, 128.6, 127.9, 127.8, 125.0, 124.9, 123.6, 123.3, 114.7; GC‐MS: *m/z* = 379 (M^+^, 100), 351 (3), 302 (51), 273 (27), 171 (16), 127 (13), 105 (68), 77 (96); HRMS‐ESI (*m/z*): [(M+H)^+^] calcd for (C_25_H_18_NOS)^+^: 380.1104; found, 380.1123.

*(Z)‐4‐(Furan‐3‐yl)‐N,5‐diphenyl‐7H‐thieno[2,3‐c]pyran‐7‐imine* (**6nc**). Yield: 55.3 mg, starting from 106.5 mg of (*Z*)‐4‐iodo‐*N*,5‐diphenyl‐7*H‐*thieno[2,3‐*c*]pyran‐7‐imine **2n** (60%; Table 5, Entry 7). Yellow solid, mp = 139°C–141°C. IR (KBr): *ν* = 1652 (s), 1591 (m), 1487 (w), 1445 (w), 1206 (w), 1051 (m), 1013 (m), 873 (w), 760 (m) cm^−1^; ^1^H NMR (500 MHz, CDCl_3_): *δ* = 7.58 (d, *J* = 5.2, 1H), 7.50 (t, *J* = 1.5, 1H), 7.39‐7.29 (m, 8H), 7.29‐7.19 (m, 2H), 7.09 (tt, *J* = 6.5, 2.1, 1H), 7.04 (d, *J* = 5.2, 1H), 6.32 (d, *J* = 1.5, 1H); ^13^C NMR (125 MHz, CDCl_3_): *δ* = 151.5, 146.8, 145.9, 143.5, 143.4, 141.32, 141.28, 132.7, 132.2, 129.1, 128.7, 128.6, 128.0, 124.8, 123.7, 123.3, 119.3, 112.2, 105.7; GC‐MS: *m/z* = 379 (M^+^, 100), 351 (3), 302 (51), 273 (27), 171 (16), 127 (13), 105 (68), 77 (96); HRMS‐ESI (*m/z*): [(M+H)^+^] calcd for (C_23_H_16_NO_2_S)^+^: 370.0896; found, 370.0895.

### Representative Suzuki–Miyaura Coupling Procedure on a Larger Scale

To a solution of (*Z*)‐5‐Butyl‐4‐iodo‐*N*‐phenyl‐7*H*‐thieno[2,3‐*c*]pyran‐7‐imine **2l** (409.0 mg, 1.00 mmol) in Bet/Gly (1/2, molar ratio; 5 mL) were added PdCl_2_ (18.0 mg, 0.1 mmol), Na_2_CO_3_ (212.0 mg, 2.0 mmol), and *p*‐tolylboronic acid (204.0 mg, 1.5 mmol). The reaction mixture was allowed to stir at 100°C (oil bath) under nitrogen in a sealed tube for 15 h and then cooled to room temperature. Et_2_O (5 mL) (was added, the mixture heated to 60°C, cooled to room temperature, and phases were separated. This extraction procedure was repeated for additional five times. After evaporation of the solvent from the collected ethereal phases, product **6lb** was purified by column chromatography on alumina using as eluent pure hexane to 98:2 hexane–AcOEt (yield: 252 mg, 68%).

## Conflict of Interests

The authors declare no conflicts of interest.

## Supporting information



Supporting Information

## Data Availability

The data that support the findings of this study are available from the corresponding author upon reasonable request.
