# Supplementary material for: Choline Chloride/Urea – Promoted Iodocyclization of 3‐Alkynylthiophene‐2‐carboxamides: A Green Synthesis of 4‐Iodo‐7H‐thieno[2,3‐c]pyran‐7‐imines and Their Coupling Reactions in Deep Eutectic Solvents
Source: Chemistry. 2025 Apr 16;31(27):e202500081. doi: 10.1002/chem.202500081 (PMC12080309; doi:10.1002/chem.202500081)
Supplement: Supplementary file 1 — Supporting Information [file CHEM-31-e202500081-s001.docx]

**Supporting Information for**

**Choline Chloride/Urea – Promoted Iodocyclization of 3-Alkynylthiophene-2-carboxamides: A Green Synthesis of 4-Iodo-7*H*-thieno[2,3-*c*]pyran-7-imines and their Coupling Reactions in Deep Eutectic Solvents**

Raffaella Mancuso,*^[a]^ Patrizio Russo,^[a,1]^ Ida Ziccarelli,^[a]^ Melania Lettieri,^[a]^ Angela Altomare,^[b]^ Matteo Tiecco,^[c]^ Edoardo Mosconi,^[d]^ Tommaso Moretti,^[e]^ and Bartolo Gabriele*^[a]^

^[a]^ Prof. Dr. R. Mancuso, Dr. P. Russo, Dr. I. Ziccarelli, Dr. M. Lettieri, Prof. Dr. B. Gabriele, Laboratory of Industrial and Synthetic Organic Chemistry (LISOC), Department of Chemistry and Chemical Technologies, University of Calabria, 87036 Arcavacata di Rende (CS), Italy

Email: [raffaella.mancuso@unical.it](mailto:raffaella.mancuso@unical.it) (R.M.), [bartolo.gabriele@unical.it](mailto:bartolo.gabriele@unical.it) (B.G.)

^[b]^ Dr. A. Altomare, Institute of Crystallography, National Research Council, Via Amendola, 122/O, 70126 Bari, Italy

^[c]^ Dr. M. Tiecco, School of Pharmacy, ChIP Research Center, University of Camerino, Via Madonna Delle Carceri, 62032 Camerino (MC), Italy

^[d]^ Prof. E. Mosconi, Computational Laboratory for Hybrid and Organic Photovoltaics Istituto CNR di Scienze e Tecnologie Chimiche (SCITEC-CNR) c/o Department of Chemistry, Biology and Biotechnologies, University of Perugia, Italy

^[e]^ Dr. T. Moretti, Department of Chemistry, Biology and Biotechnology, University of Perugia, Via Elce di Sotto 8, 06123, Perugia, Italy

^[1]^ Co-first author

Table of Contents

Pages S2–S5 Preparation and Characterization of 3-Alkynylthiophene-2-carboxamides 1

Pages S6–S13 X-Ray Crystallographic Data Collection and Structure Refinement for Products 2n and 6na

Page S14 Computational Details

Page S15 *E*-factor calculations

Page S16 References

Pages S17−S33 Copies of HRMS Spectra

Pages S34−S133 Copies of ^1^H NMR and ^13^C NMR Spectra

**Preparation and Characterization of 3-Alkynylthiophene-2-carboxamides 1**

3-Alkynylthiophene-2-carboxamides 1a-1s were prepared by Sonogashira coupling between *N*-substituted 3-bromo-2-carboxamides^[1]^ and terminal alkynes as described below.

A solution of *N*-substituted 3-bromo-2-carboxamide (4.0 mmol; 3-bromo-*N*-butylthiophene-2-carboxamide, 1.05 g; *N*-benzyl-3-bromothiophene-2-carboxamide, 1.18 g; 3-bromo-*N*-(tert-butyl)thiophene-2-carboxamide, 1.05 g; 3-bromo-*N*-phenylthiophene-2-carboxamide, 1.13 g; 3-bromo-*N*-(4-(*tert*-butyl)phenyl)thiophene-2-carboxamide, 1.35 g), PdCl_2_(PPh_3_)_2_ (280.0 mg, 0.4 mmol), CuI (46.0 mg, 0.24 mmol), and the terminal alkyne (8.0 mmol; hex-1-yne, 655 mg; 3,3-dimethylbut-1-yne, 656 mg; ethynylbenzene, 816 mg; 1-ethynyl-4-methylbenzene, 928 mg; 1-chloro-4-ethynylbenzene, 1.09 g; 1-ethynylcyclohex-1-ene, 850 mg; 3-ethynylthiophene, 865 mg) in anhydrous diisopropylamine (40 mL) was allowed to stir under nitrogen at 70 °C for 15 h. Water (70 mL) was then added, and the mixture extracted with diethyl ether (3 × 70 mL). The combine organic layers were washed with a saturated solution of NH_4_Cl (130 mL) and water until neutral pH. After drying over Na_2_SO_4_, the solvent was evaporated, and the residue purified by column chromatography on silica gel using as eluent pure hexane to 9:1 (for **1a**, **1c**, **1e**-**1f**, **1h**-**1k**, **1r**, **1s**) or pure hexane to 8:2 hexane‒AcOEt (for **1b**, **1d**, **1g**, **1l-1q**).

*N-Butyl-3-(hex-1-yn-1-yl)thiophene-2-carboxamide* (**1a**)*.* Yield: 985 mg, starting from 1.05 g of 3-bromo-*N*-butylthiophene-2-carboxamide (93%). Yellow oil. IR (film): ν = 3389 (m), 2223 (w), 1648 (s), 1535 (s), 1465 (w), 1379 (w), 1284 (m), 1231 (w), 767 (m) cm^−1^; ^1^H NMR (CDCl_3_, 500 MHz): δ = 7.62 (s, br, 1H), 7.36 (dist d, *J* = 5.0, 1H), 7.04 (dist d, *J* = 5.0, 1H), 3.50-3.42 (m, 2H), 2.50 (t, *J* = 7.1, 2H), 1.67-1.56 (m, 2H), 1.54-1.39 (m, 4H), 0.970 (t, *J* = 7.3, 3H), 0.966 (t, *J* = 7.4, 3H); ^13^C NMR (CDCl_3_, 125 MHz): δ = 161.2, 141.0, 131.7, 128.7, 120.4, 97.8, 75.7, 39.4, 31.7, 30.6, 22.1, 20.2, 19.3, 13.8, 13.6; GC-MS (EI, 70 eV) *m/z* = 263 (M^+^, 53), 234 (24), 221 (100), 206 (17), 191 (44), 178 (47), 165 (34), 135 (15), 121 (22); HRMS-ESI (*m/z*): [(M+H)^+^] calcd for (C_15_H_22_NOS)^+^: 264.1417; found, 264.1444.

*N-Butyl-3-(3,3-dimethylbut-1-yn-1-yl)thiophene-2-carboxamide* (**1b**)*.* Yield: 1.03 g, starting from 1.05 g of 3-bromo-*N*-butylthiophene-2-carboxamide (98%). Yellow solid, mp = 56-58 °C. IR (KBr): ν = 3387 (m), 2214 (w), 1639 (s), 1533 (s), 1455 (m), 1289 (m), 1141 (w), 873 (w), 778 (m) cm^−1^; ^1^H NMR (CDCl_3_, 500 MHz): δ = 7.61 (s, br, 1H), 7.36 (dist d, *J* = 5.0, 1H), 7.04 (d, *J* = 5.0, 1H), 3.50-3.43 (m, 2H), 1.62 (quint, *J* = 7.3, 2H), 1.44 (sextuplet, *J* = 7.3, 2H), 1.37 (s, 9H), 0.96 (t, *J* = 7.3, 3H); ^13^C NMR (CDCl_3_, 125 MHz): δ = 160.4, 140.0, 130.8, 127.8, 119.3, 104.4, 73.3, 38.6, 30.9, 29.84, 29.81, 19.3, 12.8; GC-MS (EI, 70 eV) *m/z* = 262 (M^+^, 41), 248 (100), 206 (30), 191 (30), 163 (9), 148 (15); HRMS-ESI (*m/z*): [(M+H)^+^] calcd for (C_15_H_22_NOS)^+^: 264.1417; found, 264.1424.

*N-Butyl-3-(phenylethynyl)thiophene-2-carboxamide* (**1c**)*.* Yield: 1.1 g, starting from 1.04 g of 3-bromo-*N*-butylthiophene-2-carboxamide (98%). Light brown solid, mp = 92-94°C. IR (KBr): ν = 3396 (m), 2198 (w), 1637 (s), 1535 (s), 1411 (w), 1381 (w), 1263 (w), 769 (m), 691 (m) cm^−1^; ^1^H NMR (CDCl_3_, 500 MHz): δ = 7.58-7.47 (m, 3H), 7.45-7.35 (m, 4H), 7.17 (d, *J* = 5.1, 1H), 3.53-3.45 (m, 2H), 1.59 (quint, *J* = 7.4, 2H), 1.40 (sextuplet, *J* = 7.4, 2H), 0.87 (t, *J* = 7.4, 3H); ^13^C NMR (CDCl_3_, 125 MHz): δ = 161.0, 141.9, 131.7, 131.5, 129.4, 129.0, 128.7, 121.7, 119.4, 95.7, 83.6, 39.6, 31.6, 20.2, 13.7; GC-MS (EI, 70 eV) *m/z* = 283 (M^+^, 24), 268 (13), 240 (31), 227 (70), 211 (100), 184 (13), 139 (79); HRMS-ESI (*m/z*): [(M+H)^+^] calcd for (C_17_H_18_NOS)^+^: 284.1104; found, 284.1117. The spectroscopic data agreed with those reported.^[2]^

*N-Butyl-3-(p-tolylethynyl)thiophene-2-carboxamide* (**1d**)*.* Yield: 1.13 g, starting from 1.05 g of 3-bromo-*N*-butylthiophene-2-carboxamide (95%). Yellow solid, mp = 72-74°C. IR (KBr): ν = 3395 (m, br), 2203 (w), 1643 (s), 1534 (s), 1414 (w), 1265 (w), 816 (m), 767 (m) cm^−1^; ^1^H NMR (CDCl_3_, 500 MHz): δ = 7.57 (s, br, 1H), 7.44-7.38 (m, 3H), 7.23-7.18 (m, 2H), 7.16 (dist d, *J* = 5.0, 1H), 2.39 (s, 2H), 1.63-1.54 (m, 2H), 1.45-1.34 (m, 2H), 0.88 (t, *J* = 7.4, 3H); ^13^C NMR (CDCl_3_, 125 MHz): δ = 161.1, 141.6, 139.7, 131.4, 129.5, 129.0, 119.7, 118.5, 96.1, 83.0, 39.6, 31.6, 21.6, 20.2, 13.7; GC-MS (EI, 70 eV) *m/z* = 297 (M^+^, 41), 254 (32), 241 (75), 225 (100), 206 (14), 153 (66); HRMS-ESI (*m/z*): [(M+H)^+^] calcd for (C_18_H_20_NOS)^+^: 298.1260; found, 298.1282.

*N-Butyl-3-((4-chlorophenyl)ethynyl)thiophene-2-carboxamide* (**1e**)*.* Yield: 1.24 g, starting from 1.04 g of 3-bromo-*N*-butylthiophene-2-carboxamide (98%). Yellow solid, mp = 95-97°C. IR (KBr): ν = 33307 (m, br), 2203 (vw), 1639 (s), 1536 (m), 1487 (w), 1415 (w), 1267 (w), 1093 (w), 1014 (w), 825 (w), 772 (s) cm^−1^; ^1^H NMR (CDCl_3_, 500 MHz): δ = 7.48-7.40 (m, 4H), 7.40-7.35 (m, 2H), 7.17 (dist d, *J* = 5.1, 1H), 3.52-3.45 (m, 2H), 1.58 (quint, *J* = 7.3, 2H), 1.39 (sextuplet, *J* = 7.3, 2H), 0.88 (t, *J* = 7.3, 3H); ^13^C NMR (CDCl_3_, 125 MHz): δ = 160.9, 142.2, 135.6, 132.7, 131.4, 129.1, 120.2, 119.1, 94.5, 84.5, 39.6, 31.6, 20.2, 13.7; GC-MS (EI, 70 eV) *m/z* = 319 [(M+2)^+^, 10], 317 (M^+^, 26), 276 (13), 275 (24), 274 (26), 263 (28), 261 (75), 247 (50), 245 (100), 206 (16), 173 (58); HRMS-ESI (*m/z*): [(M+H)^+^] calcd for (C_17_H_17_ClNOS)^+^: 318.0714; found, 318.0726.

*N-Butyl-3-(thiophen-3-ylethynyl)thiophene-2-carboxamide* (**1f**)*.* Yield: 980 mg, starting from 1.05 g of 3-bromo-*N*-butylthiophene-2-carboxamide (85%). Yellow solid, mp = 87-88°C. IR (KBr): ν = 3395 (m, br), 2205 (w), 1645 (s), 1530 (m), 1404 (w), 1290 (w), 1138 (w), 997 (w), 766 (m) cm^−1^; ^1^H NMR (CDCl_3_, 500 MHz): δ = 7.58 (s, br, 1H), 7.50 (s, br, 1H), 7.44-7.39 (m, 1H), 7.39-7.34 (m, 1H), 7.19 (dist d, *J* = 4.8, 1H), 7.16 (dist d, *J* = 7.4, 1H), 3.52-3.44 (m, 2H), 1.63-1.54 (m, 2H), 1.46-1.35 (m, 2H), 0.89 (t, *J* = 7.0, 3H); ^13^C NMR (CDCl_3_, 125 MHz): δ = 161.0, 141.8, 131.4, 129.9, 129.4, 129.0, 126.2, 120.7, 119.5, 91.0, 83.2, 39.6, 31.5, 20.2, 13.7; GC-MS (EI, 70 eV) *m/z* = 289 (M^+^, 29), 260 (12), 146 (25), 233 (67), 217 (100), 190 (17), 145 (99), 108 (18); HRMS-ESI (*m/z*): [(M+H)^+^] calcd for (C_15_H_16_NOS_2_)^+^: 290.0668; found, 290.0663.

*N-Butyl-3-(cyclohex-1-en-1-ylethynyl)thiophene-2-carboxamide* (**1g**)*.* Yield: 1.11 g, starting from 1.05 g of 3-bromo-*N*-butylthiophene-2-carboxamide (96%). Yellow solid, mp = 58-60°C. IR (KBr): ν = 3391 (m, br), 2195 (w), 1643 (s), 1537 (m), 1436 (w), 1384 (w), 1291 (w), 1244 (w), 1138 (w), 990 (w), 768 (m) cm^−1^; ^1^H NMR (CDCl_3_, 500 MHz): δ = 7.54 (s, br, 1H), 7.37 (dist d, *J* = 5.0, 1H), 7.06 (dist d, *J* = 5.0, 1H), 6.30-6.26 (m, 1H), 3.50-3.43 (m, 2H), 2.30-2.14 (m, 4H), 1.77-1.55 (m, 6H), 1.49-1.36 (m, 2H), 0.95 (t, *J* = 7.3, 3H); ^13^C NMR (CDCl_3_, 125 MHz): δ = 161.2, 141.0, 137.4, 131.4, 128.9, 120.1, 119.8, 97.9, 81.2, 39.5, 31.6, 29.0, 25.9, 22.2, 21.3, 20.2, 13.8; GC-MS (EI, 70 eV) *m/z* = 287 (M^+^, 100), 270 (18), 258 (8), 244 (11), 230 (14), 215 (43), 202 (13), 187 (29), 172 (12), 115 (15); HRMS-ESI (*m/z*): [(M+H)^+^] calcd for (C_17_H_22_NOS)^+^: 288.1417; found, 288.1426.

*N-Benzyl-3-(hex-1-yn-1-yl)thiophene-2-carboxamide* (**1h**)*.* Yield: 680 mg, starting from 1.18 g of *N*-benzyl-3-bromothiophene-2-carboxamide (57%). Yellow solid, mp = 69-71°C. IR (KBr): ν = 3379 (m), 2223 (w), 1647 (s), 1540 (m), 1456 (w), 1281 (w), 766 (m) 727 (m), 699 (m) cm^−1^; ^1^H NMR (CDCl_3_, 500 MHz): δ = 7.92 (s, br, 1H), 7.41-7.26 (m, 6H), 7.03 (dist d, *J* = 5.0, 1H), 4.64 (dist d, *J* = 5.2, 2H), 2.21 (t, *J* = 6.7, 2H), 1.39-1.21 (m, 4H), 0.84 (t, *J* = 7.3, 3H); ^13^C NMR (CDCl_3_, 125 MHz): δ = 161.1, 140.4, 137.9, 131.6, 129.1, 128.8, 128.0, 127.7, 120.8, 98.3, 75.5, 44.1, 30.2, 22.0, 19.1, 13.5; GC-MS (EI, 70 eV) *m/z* = 297 (M^+^, 62), 255 (98), 240 (48), 237 (48), 220 (51), 206 (65), 178 (43), 164 (82), 150 (42), 136 (51), 121 (76), 91 (100), 77 (56); HRMS-ESI (*m/z*): [(M+H)^+^] calcd for (C_18_H_20_NOS)^+^: 298.1260; found, 298.1267.

*N-Benzyl-3-(phenyethynyl)thiophene-2-carboxamide* (**1i**)*.* Yield: 805 mg, starting from 1.19 g of *N*-benzyl-3-bromothiophene-2-carboxamide (63%). Yellow solid, mp = 89-91°C. IR (KBr): ν = 3391 (m), 2205 (w), 1647 (s), 1530 (m), 1454 (w), 1383 (w), 1220 (w), 772 (s) cm^−1^; ^1^H NMR (CDCl_3_, 500 MHz): δ = 7.83 (s, br, 1H), 7.44 (d, *J* = 5.1, 1H), 7.41-7.36 (m, 2H), 7.33-7.28 (m, 4H), 7.25-7.20 (m, 2H), 7.17 (d, *J* = 5.1, 1H), 7.06-7.02 (m, 2H), 4.67 (dist d, *J* = 5.1, 2H); ^13^C NMR (CDCl_3_, 125 MHz): δ = 160.9, 141.2, 137.7, 131.5, 131.4, 129.4, 129.2, 129.0, 128.5, 128.4, 127.8, 121.4, 120.0, 96.2, 83.5, 44.4; GC-MS (EI, 70 eV) *m/z* = 317 (M^+^, 29), 300 (58), 271 (12), 240 (18), 212 (23), 184 (37), 152 (12), 139 (100), 113 (13), 91 (46); HRMS-ESI (*m/z*): [(M+H)^+^] calcd for (C_20_H_16_NOS)^+^: 318.0947; found, 318.0950.

*N-(tert-Butyl)-3-(hex-1-yn-1-yl)thiophene-2-carboxamide* (**1j**)*.* Yield: 998 mg, starting from 1.04 g of 3-bromo-*N*-(*tert*-butyl)thiophene-2-carboxamide (96%). Yellow solid, mp = 96-97°C. IR (KBr) ν = 3367 (m), 2219 (w), 1648 (m), 1547 (w), 1453 (w), 1377 (m), 1220 (s), 773 (s) cm^−1^; ^1^H NMR (CDCl_3_, 500 MHz): δ = 7.58 (s, br, 1H), 7.33 (dist d, *J* = 5.0, 1H), 7.03 (d, *J* = 5.0, 1H), 2.48 (t, *J* = 7.3, 2H), 1.63 (quint, *J* = 7.3, 2H), 1.53-1.42 (m, 11H), 0.96 (t, *J* = 7.3, 3H); ^13^C NMR (CDCl_3_, 125 MHz): δ = 160.5, 142.7, 131.7, 128.3, 120.0, 97.9, 75.5, 51.6, 30.5, 28.9, 22.1, 19.3, 13.6; GC-MS (EI, 70 eV) *m/z* = 263 (M^+^, 59), 221 (83), 207 (84), 191 (97), 178 (88), 165 (100), 149 (52), 135 (55), 121 (77); HRMS-ESI (*m/z*): [(M+H)^+^] cald for (C_15_H_22_NOS)^+^: 264.1417; found: 264.1419.

*N-(tert-Butyl)-3-(phenylethynyl)thiophene-2-carboxamide* (**1k**)*.* Yield: 1.12 g, starting from 1.05 g of 3-bromo-*N*-(*tert*-butyl)thiophene-2-carboxamide (98%). Yellow solid, mp = 94-95°C. IR (KBr): ν = 3386 (s), 2206 (w), 1656 (s), 1534 (m), 1392 (w), 1364 (w), 1302 (w), 1219 (m), 1070 (w), 986 (w), 834 (m), 757 (m) cm^−1^; ^1^H NMR (CDCl_3_, 500 MHz): δ = 7.56-7.50 (m, 2H), 7.48 (s, br, 1H), 7.42-7.35 (m, 2H), 7.18-7.13 (m, 1H), 1.45 (s, 9H); ^13^C NMR (CDCl_3_, 125 MHz): δ = 160.2, 143.2, 131.58, 131.55, 131.50, 129.4, 128.7, 121.7, 119.1, 95.9, 83.4, 51.8, 28.9; GC-MS (EI, 70 eV) *m/z* = 283 (M^+^, 10), 227 (100), 211 (92), 149 (14), 139 (89), 113 (13); HRMS-ESI (*m/z*): [(M +H)^+^] cald for (C_17_H_18_NOS)^+^: 284.1104; found: 284.1100.

*3-(Hex-1-yn-1-yl)-N-phenylthiophene-2-carboxamide* (**1l**)*.* Yield: 1.11 g, starting from 1.13 g of 3-bromo-*N*-phenylthiophene-2-carboxamide (98%). Yellow oil. IR (film): ν = 3351 (m), 2221 (w), 1661 (s), 1600 (m), 1499 (m), 1444 (m), 1379 (m), 1316 (m), 1247 (w), 1062 (w), 760 (s) cm^−1^; ^1^H NMR (CDCl_3_, 500 MHz): δ = 9.51 (s, br, 1H), 7.64-7.55 (m, 2H), 7.42 (dist d, *J* = 5.0, 1H), 7.38-7.30 (m, 2H), 7.16-7.10 (m, 1H), 7.09 (dist d, *J* = 5.0, 1H), 2.58 (t, *J* = 7.2, 2H), 1.71-1.61 (m, 2H), 1.55-1.42 (m, 2H), 0.95 (t, *J* = 7.3, 3H); ^13^C NMR (CDCl_3_, 125 MHz): δ = 159.2, 140.9, 137.8, 131.9, 129.7, 129.1, 124.4, 120.9, 120.0, 99.3, 75.5, 30.6, 22.1, 19.4, 13.6; GC-MS (EI, 70 eV) *m/z* = 283 (M^+^, 45), 266 (16), 241 (80), 191 (100), 149 (22), 135 (13), 121 (19); HRMS-ESI (*m/z*): [(M+H)^+^] calcd for (C_17_H_18_NOS)^+^: 284.1104; found, 284.1120.

*3-(3,3-Dimethylbut-1-yn-1-yl)-N-phenylthiophene-2-carboxamide* (**1m**)*.* Yield: 1.08 g, starting from 1.13 g of 3-bromo-*N*-phenylthiophene-2-carboxamide (95%). Yellow oil. IR (film): ν = 3356 (m), 2212 (w), 1659 (s), 1598 (m), 1444 (m), 1376 (w), 1315 (w), 1252 (m), 1062 (w), 881 (w), 760 (m) cm^−1^; ^1^H NMR (CDCl_3_, 500 MHz): δ = 9.36 (s, br, 1H), 7.68-7.60 (m, 2H), 7.43 (dist d, *J* = 5.2, 1H), 7.42-7.34 (m, 2H), 7.18-7.12 (m, 2H), 7.10 (dist d, *J* = 5.2, 1H), 1.41 (s, 9H); ^13^C NMR (CDCl_3_, 125 MHz): δ = 159.3, 140.5, 137.6, 132.2, 129.7, 129.1, 124.6, 120.9, 120.4, 107.0, 74.0, 30.9, 28.6; GC-MS (EI, 70 eV) *m/z* = 283 (M^+^, 55), 268 (79), 191 (100), 148 (28); HRMS-ESI (*m/z*): [(M+H)^+^] calcd for (C_17_H_18_NOS)^+^: 284.1104; found, 284.1113.

*N-Phenyl-3-(phenylethynyl)thiophene-2-carboxamide* (**1n**)*.* Yield: 1.12 mg, starting from 1.13 g of 3-bromo-*N*-phenylthiophene-2-carboxamide (92%). Yellow solid, mp = 128-130°C. IR (KBr): ν = 3362 (m), 2204 (w), 1659 (s), 1598 (m), 1541 (m), 1499 (w), 1443 (m), 1379 (w), 1316 (w), 1251 (w), 771 (s) cm^−1^; ^1^H NMR (CDCl_3_, 500 MHz): δ = 9.43 (s, br, 1H), 7.67-7.55 (m, 4H), 7.49 (dist d, *J* = 5.1, 1H), 7.47-7.37 (m, 3H), 7.35-7.28 (m, 2H), 7.22 (dist d, *J* = 5.1, 1H), 7.12 (dist t, *J* = 7.4, 1H); ^13^C NMR (CDCl_3_, 125 MHz): δ = 159.0, 141.8, 137.7, 131.7, 131.6, 130.0, 129.7, 129.2, 128.9, 124.5, 121.3, 119.8, 113.0, 97.2, 83.3; GC-MS (EI, 70 eV) *m/z* = 303 (M^+^, 46), 274 (4), 211 (100), 183 (2), 139 (46); HRMS-ESI (*m/z*): [(M+H)^+^] calcd for (C_19_H_14_NOS)^+^: 304.0791; found, 340.0800.

*N-phenyl-3-(p-tolylethynyl)thiophene-2-carboxamide* (**1o**)*.* Yield: 1.20 g, starting from 1.12 g of 3-bromo-*N*-phenylthiophene-2-carboxamide (95%). Yellow solid, mp = 124-125 °C. IR (KBr): ν = 3361 (m), 2202 (w), 1659 (s), 1599 (m), 1539 (s), 1499 (w), 1445 (m), 1381 (w), 1317 (w), 1252 (m), 816 (m), 759 (s) cm^−1^; ^1^H NMR (CDCl_3_, 500 MHz): δ = 9.43 (s, br, 1H), 7.63-7.58 (m, 2H), 7.52-7.46 (m, 3H), 7.35-7.29 (m, 2H), 7.24-7.20 (m, 3H), 7.15-7.09 (m, 1H); ^13^C NMR (CDCl_3_, 125 MHz): δ = 159.1, 141.6, 140.1, 137.8, 131.7, 131.5, 130.0, 129.6, 129.2, 124.5, 120.3, 119.9, 118.3, 97.5, 82.8, 21.7; GC-MS (EI, 70 eV) *m/z* = 317 (M^+^, 43), 288 (3), 225 (100), 197 (3), 153 (40); HRMS-ESI (*m/z*): [(M+H)^+^] calcd for (C_20_H_16_NOS)^+^: 318.0947; found, 318.0946.

*3-((4-Chlorophenyl)ethynyl)-N-phenylthiophene-2-carboxamide* (**1p**)*.* Yield: 1.2 g, starting from 1.13 g of 3-bromo-*N*-phenylthiophene-2-carboxamide (89%). Yellow solid, mp = 133-135°C. IR (KBr): ν = 3368 (m), 2209 (w), 1659 (s), 1599 (m), 1539 (s), 1486 (w), 1443 (m), 1380 (w), 1317 (m), 1252 (m), 1220 (w), 1095 (w), 825 (m), 752 (s) cm^−1^; ^1^H NMR (CDCl_3_, 500 MHz): δ = 9.33 (s, br, 1H), 7.61-7.55 (m, 2H), 7.52-7.45 (m, 3H), 7.41-7.35 (m, 2H), 7.35-7.28 (m, 2H), 7.20 (dist d, *J* = 5.0, 1H), 7.12 (t, *J* = 7.3, 1H); ^13^C NMR (CDCl_3_, 125 MHz): δ = 158.8, 142.1, 137.6, 135.9, 132.8, 131.6, 130.1, 129.24, 129.22, 124.6, 119.8, 119.6, 95.8, 84.2; GC-MS (EI, 70 eV) *m/z* = 339 [(M+2)^+^, 17], 337 (M^+^, 41), 273 (2), 247 (38), 245 (100), 217 (2), 175 (11), 173 (34), 138 (6); HRMS-ESI (*m/z*): [(M+H)^+^] calcd for (C_19_H_13_ClNOS)^+^: 338.0401; found, 338.0406.

*3-(Cyclohex-1-en-1-ylethynyl)-N-phenylthiophene-2-carboxamide* (**1q**)*.* Yield: 1.1 g, starting from 1.13 g of 3-bromo-*N*-phenylthiophene-2-carboxamide (89%). Colorless solid, mp = 139-140°C. IR (KBr): ν = 3355 (m), 2193 (w), 1659 (s), 1599 (m), 1541 (s), 1445 (m), 1382 (w), 1316 (m), 1247 (w), 759 (m) cm^−1^; ^1^H NMR (CDCl_3_, 500 MHz): δ = 9.45 (s, br, 1H), 7.67-7.60 (m, 2H), 7.45 (d, *J* = 5.1, 1H), 7.40-7.32 (m, 2H), 7.17-7.10 (m, 2H), 6.39 (s, 1H), 2.35-2.14 (m, 4H), 1.78-1.57 (m, 4H); ^13^C NMR (CDCl_3_, 125 MHz): δ = 159.2, 140.9, 138.1, 137.8, 131.7, 129.8, 129.1, 124.4, 120.7, 120.0, 119.7, 99.3, 80.9, 29.0, 25.9, 22.1, 21.3; GC-MS (EI, 70 eV) *m/z* = 307 (M^+^, 73), 278 (5), 215 (100), 187 (16), 171 (13), 115 (22); HRMS-ESI (*m/z*): [(M+H)^+^] calcd for (C_19_H_18_NOS)^+^: 308.1104; found, 308.1112.

*N-(4-tert-Butyl)phenyl)-3-(hex-1-yn-1-yl)thiophene-2-carboxamide* (**1r**)*.* Yield: 1.21 g, starting from 1.35 g of 3-bromo-*N*-(4-*tert*-butyl)phenylthiophene-2-carboxamide (89%). Yellow oil. IR (film): ν = 3353 (m), 2222 (w), 1662 (s), 1597 (m), 1524 (s), 1466 (w), 1412 (m), 1378 (w), 1319 (m), 1270 (m), 1063 (w), 835 (w), 760 (m) cm^−1^; ^1^H NMR (CDCl_3_, 500 MHz): δ = 9.48 (s, br, 1H), 7.57-7.52 (m, 2H), 7.44-7.41 (m, 1H), 7.41-7.36 (m, 2H), 7.10 (dist d, *J* = 5.4, 1H), 2.58 (t, *J* = 7.2, 2H), 1.68 (quint, *J* = 7.2, 2H), 1.51 (hexuplet, *J* = 7.2, 2H), 1.48 (s, 9H), 0.95 (t, *J* = 7.2, 3H); ^13^C NMR (CDCl_3_, 125 MHz): δ = 158.2, 146.5, 140.2, 134.2, 131.0, 128.6, 125.0, 119.8, 118.9, 98.2, 74.3, 33.5, 30.4, 29.7, 21.3, 18.5, 12.7; GC-MS (EI, 70 eV) *m/z* = 339 (M^+^, 48), 297 (65), 191 (100), 149 (23), 135 (26), 121 (25); HRMS-ESI (*m/z*): [(M+H)^+^] calcd for (C_21_H_26_NOS)^+^: 340.1730; found, 340.1737.

*N-(4-tert-Butyl)phenyl)-3-(phenylethynyl)thiophene-2-carboxamide* (**1s**)*.* Yield: 1.34 g, starting from 1.35 g of 3-bromo-*N*-(4-*tert*-butyl)phenylthiophene-2-carboxamide (93%). Yellow oil. IR (film): ν = 3364 (m), 2204 (w), 1659 (s), 1597 (m), 1531 (s), 1413 (m), 1319 (w), 1251 (m), 757 (m) cm^−1^; ^1^H NMR (CDCl_3_, 500 MHz): δ = 9.44 (s, br, 1H), 7.63-7.57 (m, 2H), 7.56-7.52 (m, 2H), 7.48 (dist d, *J* = 4.9, 1H), 7.46-7.39 (m, 3H), 7.37-7.32 (m, 2H), 7.22 (dist d, *J* = 4.9, 1H), 1.30 (s, 9H); ^13^C NMR (CDCl_3_, 125 MHz): δ = 158.9, 147.5, 142.2, 135.1, 131.7, 131.6, 128.8, 129.7, 128.8, 126.0, 121.4, 119.8, 119.6, 97.0, 83.3, 34.4, 31.3; GC-MS (EI, 70 eV) *m/z* = 359 (M^+^, 77), 344 (93), 302 (5), 211 (100), 183 (7), 158 (9), 139 (99), 113 (9); HRMS-ESI (*m/z*): [(M+H)^+^] calcd for (C_23_H_22_NOS)^+^: 360.1417; found, 360.1426.

**X-Ray Crystallographic Data Collection and Structure Refinement for Products 2n and 6na^[3]^**

**
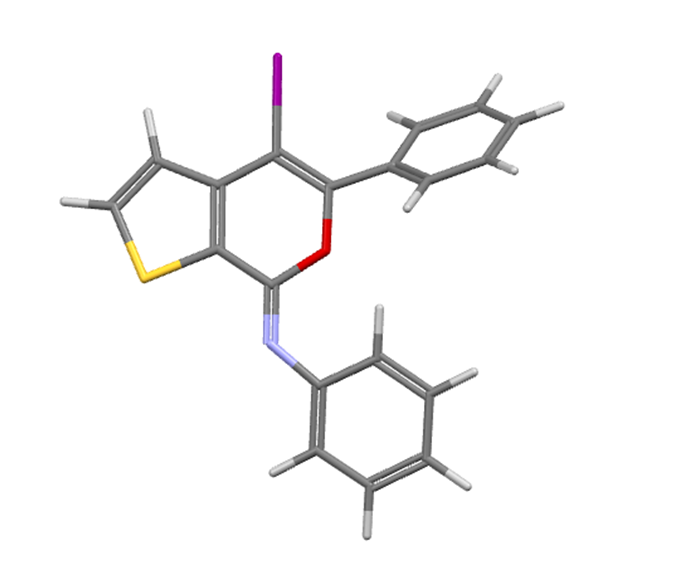
**

**Figure S1.** The asymmetric unit of the **2n** compound. Color legend: carbon (light grey), hydrogen (white), oxygen (red), nitrogen (light blue), sulphur (yellow), iodine (purple).

**Table S1.** X-Ray Data of **2n**

| **Crystal data** | |
| --- | --- |
| CCDC number | 2363245 |
| Chemical formula | C_19_H_12_INOS |
| Formula weight (g/mol) | 429.28 |
| Crystal system | Orthorhombic |
| Space group | *Pbca* |
| Temperature (K) | 293 |
| Cell parameters (Å) | *a* = 14.4897 (4)  *b* = 20.2316 (6)  *c* = 11.2545 (3) |
| Volume (Å^3^) | 3299.23 (17) |
| Z | 8 |
| Z’ | 1 |
| Radiation type | Cu *Kα* radiation,  λ = 1.540560 Å |
| **Data collection** | |
| Diffractometer | Rigaku RINT2500 |
| Specimen mounting | special glass capillary |
| Data collection mode | transmission |
| 2θ (°) | 2θ_min_ = 6.00, 2θ_max_ = 120.00 |
| **Structure solution** | |
| Methods | Direct space method, direct methods |
| Parameters | 6+3 DOF |
| Cost function | *R_wp_* = 5.521 |
| **Refinement** | |
| *R_p_* | 2.315 |
| *R_wp_* | 3.233 |
| *R_exp_* | 3.583 |
| *R_Bragg_* | 6.228 |
| χ^2^ | 0.814 |
| No. of data points | 5701 |
| No. of reflections | 2455 |
| Profile function | Pearson VII |
| *Refinement parameters* | |
| Lattice | 3 |
| Positional | 69 |
| ADP | 5 |
| Profile | 10 |
| Background | 18 |
| Peak-shift | 3 |
| Restraints | 62 |
| H-atom treatment | H-atom parameters constrained |
| **Programs** | |
| Indexing | N-TREOR09^[4]^ |
| Space group determination | EXPO^[5]^ |
| Structure solution and refinement | EXPO |
| Model building | ChemSketch,^[6]^ MOPAC2016^[7]^ |
| Structure validation | Quantum ESPRESSO^[8]^ |
| Structure visualization | Mercury^[9]^ |

**
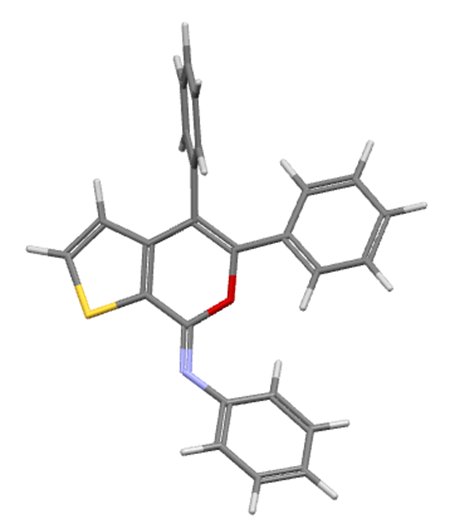
**

**Figure S2.** The asymmetric unit of the **6na** compound. Color legend: carbon (light grey), hydrogen (white), oxygen (red), nitrogen (light blue), sulphur (yellow).

**Table S2.** X-Ray Data of **6na**

| **Crystal data** | |
| --- | --- |
| CCDC number | 2363248 |
| Chemical formula | C_25_H_17_NOS |
| Formula weight (g/mol) | 379.48 |
| Crystal system | Orthorombic |
| Space group | *Pbcn* |
| Temperature (K) | 293 |
| Cell parameters (Å) | *a* = 15.2284 (5)  *b* = 10.4287 (3)  *c* = 24.1611 (7) |
| Volume (Å^3^) | 3837.08 (18) |
| Z | 8 |
| Z’ | 1 |
| Radiation type | Cu *Kα* radiation,  λ = 1.540560 Å |
| **Data collection** | |
| Diffractometer | Rigaku RINT2500 |
| Specimen mounting | special glass capillary |
| Data collection mode | transmission |
| 2θ (°) | 2θ_min_ = 6.00, 2θ_max_ = 120.00 |
| **Structure solution** | |
| Methods | Direct space method, direct methods |
| Parameters | 6+4 DOF |
| Cost function | *R_wp_* = 6.530 |
| **Refinement** | |
| *R_p_* | 2.557 |
| *R_wp_* | 3.642 |
| *R_exp_* | 2.836 |
| *R_Bragg_* | 6.526 |
| χ^2^ | 1.649 |
| No. of data points | 5701 |
| No. of reflections | 2859 |
| Profile function | Pearson VII |
| *Refinement parameters* | |
| Lattice | 3 |
| Positional | 84 |
| ADP | 4 |
| Profile | 10 |
| Background | 18 |
| Peak-shift | 3 |
| Restraints | 76 |
| H-atom treatment | H-atom parameters constrained |
| **Programs** | |
| Indexing | N-TREOR09^[4]^ |
| Space group determination | EXPO^[5]^ |
| Structure solution and refinement | EXPO |
| Model building | ChemSketch,^[6]^ MOPAC2016^[7]^ |
| Structure validation | Quantum ESPRESSO^[8]^ |
| Structure visualization | Mercury^[9]^ |

The *ab initio* solution and structure refinement were automatically performed by EXPO software,^[5]^ a package capable of carrying out the following steps: a) determination of unit-cell parameters and identification of the space group; b) structure solution by direct methods and/or a real-space approach; c) structure model refinement by the Rietveld method.^[10]^ The first low-angle well-defined peaks in the experimental diffraction pattern were selected and actively used for indexing with N-TREOR09^[4]^ and DICVOL04^[11]^ programs embedded in EXPO. The space group determination was identified based on the evaluation of the systematic absences.

The structures were solved with a real-space method based on the simulated annealing algorithm implemented in EXPO. The starting model was built using the sketching facilities of ACD/ChemSketch^[6]^ and the geometry optimization was performed by the program MOPAC2016.^[7]^ The simulated annealing algorithm was run 100 times under Linux workstation in default mode and parallel calculation over 20 CPUs. The best solution with the lowest cost function value was selected. The criterion to accept the solution was also based on the soundness of the crystal packing. The solutions obtained by the direct-space method were further confirmed by direct methods.

Density-functional theory (DFT) geometry optimization with Quantum ESPRESSO was only performed on hydrogen atoms to improve their positions.^[6]^ The resulting structures were refined by the Rietveld method. Restraints were applied to bond distances and angles to stabilize the refinement. All H atoms bonded to C atoms were treated as riding atoms under the constraint on atomic displacement parameters U_iso_(H) = 1.2U_iso_(C). Peak shape was modelled using the Pearson VII function. The atomic displacement parameters were refined isotropically with the constraint that they have the same value for each chemical species.

To validate the refined crystal structures, they were subjected to periodic, solid-state calculations performed by Quantum ESPRESSO, an *ab initio* quantum-mechanical program employing plane waves and density-functional theory to simulate the properties of solids. The following execution parameters were used: PBE potentials from the SSSP Efficiency PBE (version 1.1) library,^[12]^ an optional cut-off controlling the accuracy of the calculations set to 60 Ry, k-point spacing was 0.15 Å^‒1^, van der Waals interactions were corrected by means of a Grimme’s D3 dispersion correction.^[13]^ Atomic-coordinate-only optimization of the structures were performed using the experimental cell parameters and atomic positions obtained from the Rietveld refinement. The root-mean-square (RMS) displacement of non-H atoms between the DFT-optimized and experimental crystal structures were 0.195 Å, 0.098 Å respectively, providing strong evidence for the correctness of the experimental structures.^[14]^


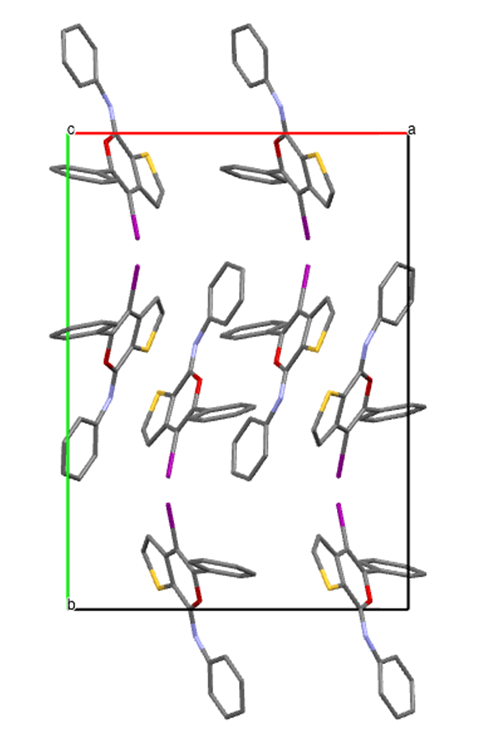


**Figure S3.** View of the packing of **2n** molecules along the c axis.

**
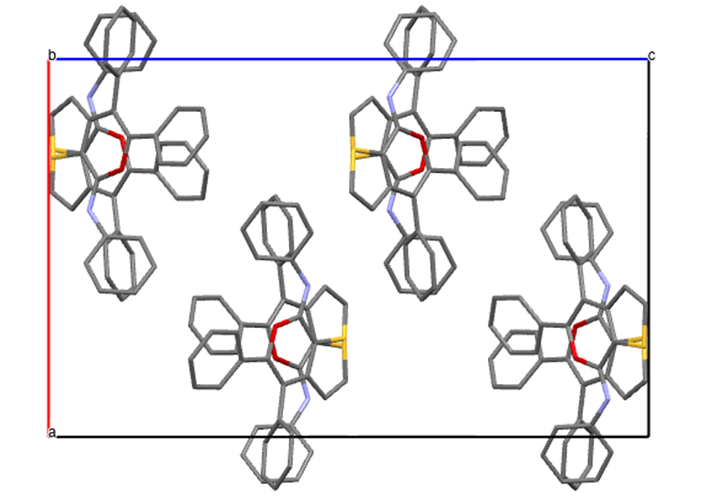
**

**Figure S4.** View of the packing of **6na** molecules along the b axis.

**Computational Details**

The B3LYP exchange-correlation functional,^[15]^ as implemented in the GAUSSIAN03 program package,^[16]^ has been used for all our DFT calculations. All geometry optimizations have been performed with a 6-31g* basis set for C, H, N, S, Cl, B and O, and SDD basis set for I and Pd along with a SDD pseudopotential.^[17]^ Solvation effects and Gibbs free energies in solution are evaluated by the conductor-like polarizable continuum model (C-PCM)^[18]^ using acetonitrile as solvent and used for geometry optimization excepted the data reported in vacuum. To initially determine the possible configuration of the transition states, a linear transit approach was adopted to simulate the energetic profile from the reagents to the product by constraining the reaction coordinate involved in the processes. From this initial reaction pathway, we select the structure with the highest energy along the constrained reaction profile and we converged the TS geometry optimization. For all systems (reagents, intermediates and products) we simulated the interacting complexes.


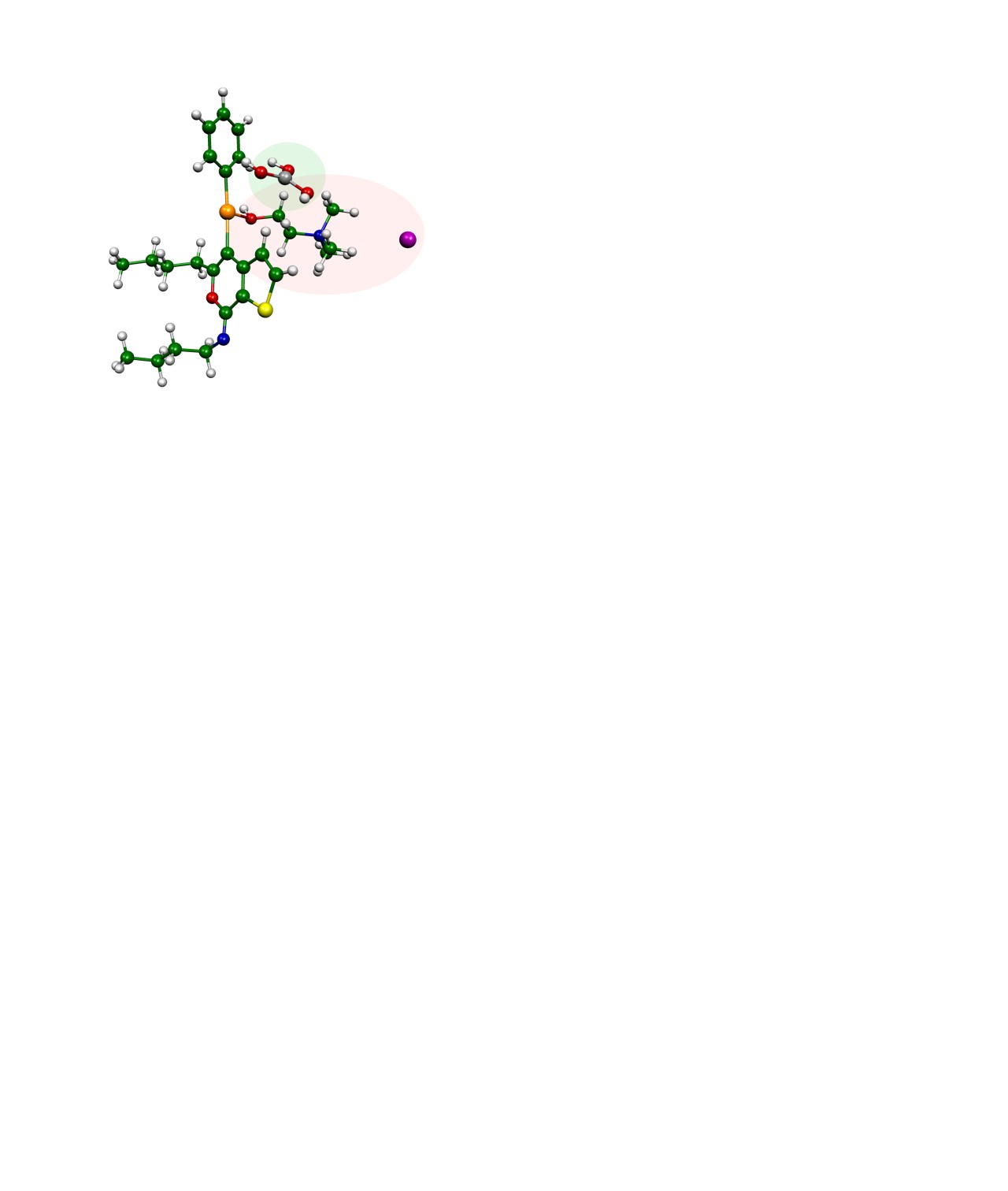


**Figure S5.** Optimized Ar'PdAr + H_3_BO_3_ + I^‒^ reaction with choline (in the red circle).

***E*-factor calculations**

The developed approach exploited the catalytic properties of the DESs, also considering the environmental advantages deriving from their green features, such as their non-volatility, recyclability and natural source of the components. The sustainability of the protocol was not optimized in terms of amounts of waste produced on the amounts of products produced (*E* factor). The calculated *E* factors were, in fact, between the values of 1500 and 3200 for all the proposed procedures where column chromatography purifications were made, mainly because of the amounts of solvents and of alumina needed for these purification protocols. In order to ameliorate these factors, three reactions of Table 2 (leading to products **2e**, **2n**, and **2s**) were repeated by avoiding any column chromatography, but recrystallizing the products after the extractions (see Table S3). In this way, the *E*-factors decrease significantly, reaching values between 600 and 1000, which can be considered further improvable but more sustainable than the column-purified ones.^[19]^

The procedure therefore can be also ameliorated in terms of sustainability, and this will be part of future works.

**Table S3.** *E* Factor calculations of the procedures by considering the column chromatography purifications (up) or the recrystallization of the products (down). All the reactants, products and solvents for the extractions and purifications were considered in grams via their densities or by weighting.

| **Product** | ***E* FACTOR** | **Products, g** | **TOTAL WASTE, g** | **Starting material, g** | **DES, g** | **I_2_, g** | **Et_2_O, g** | **H_2_O + Na_2_S_2_O_3_, g** | **Et_2_O extr., g** | **Na_2_SO_4_, g** | **Alumina column, g** | **Column solvent, g** |
| --- | --- | --- | --- | --- | --- | --- | --- | --- | --- | --- | --- | --- |
| **2e** | **2348** | 0.0864 | 202.8781 | 0.0961 | 2.4 | 0.152 | 12.78 | 21 | 31.95 | 4.5 | 10 | 120 |
| **2n** | **1999** | 0.1015 | 202.8736 | 0.0916 | 2.4 | 0.152 | 12.78 | 21 | 31.95 | 4.5 | 10 | 120 |
| **2s** | **1752** | 0.1158 | 202.8902 | 0.1082 | 2.4 | 0.152 | 12.78 | 21 | 31.95 | 4.5 | 10 | 120 |
| **Product** | ***E* FACTOR** | **Products, g** | **TOTAL WASTE, g** | **Starting material, g** | **DES, g** | **I_2_, g** | **Et_2_O, g** | **H_2_O + Na_2_S_2_O_3_, g** | **Et_2_O extr., g** | **Na_2_SO_4_, g** | **Et_2_O recr., g** | **Hexane recr., g** |
| **2e recr.** | **1034** | 0.0725 | 74.9523 | 0.0893 | 2.4 | 0.152 | 12.78 | 21 | 31.95 | 4.5 | 1.42 | 0.661 |
| **2n recr.** | **822** | 0.0912 | 74.9508 | 0.0878 | 2.4 | 0.152 | 12.78 | 21 | 31.95 | 4.5 | 1.42 | 0.661 |
| **2s recr.** | **647** | 0.1158 | 74.9712 | 0.1082 | 2.4 | 0.152 | 12.78 | 21 | 31.95 | 4.5 | 1.42 | 0.661 |

**References**

[1] R. Ferraccioli, D. Carenzi, O. Rombolà, M. Catellani, *Org. Lett.* **2004**, *6*, 4759-4762.

[2] G. Infante, S. Eisler, *Can. J. Chem.* **2017**, *95*, 415-423.

[3] X-ray Crystallographic Information Files 2n.cif, 6na.cif contain the supplementary crystallographic data for this paper, and are supplied as independent Supporting Information files for this article. These files can also be obtained free of charge from the Cambridge Crystallographic Data Centre via www.ccdc.cam.ac.uk/data_request/cif (CCDC 2363245, 2363248, respectively).

[4] A. Altomare, G. Campi, C. Cuocci, L. Eriksson, C. Giacovazzo, A. Moliterni, R. Rizzia, P.-E. Werner, *J. Appl. Cryst.* **2009**, *42*, 768-775.

[5] A. Altomare, C. Cuocci, C. Giacovazzo, A. Moliterni, R. Rizzi, N. Corriero, A. Falcicchio, *J. Appl. Cryst.* **2013**, *46*, 1231-1235.

[6] ACD/ChemSketch, Advanced Chemistry Development, Inc.: Toronto, ON, Canada, 2003.

[7] MOPAC2016, Version 18.305L, in: J. J. P. Stewart, Stewart Computational Chemistry, Colorado Springs, CO, USA. <http://OpenMOPAC.net/>

[8] P. Giannozzi, S. Baroni, N. Bonini, M. Calandra, R. Car, C. Cavazzoni, D. Ceresoli, G.L. Chiarotti, M. Cococcioni, I. Dabo, *J. Phys. Condens. Matter* **2009**, *21*, 395502.

[9] C.F. Macrae, I. Sovago, S.J. Cottrell. P.T.A. Galek, P. McCabe, E. Pidcock, M. Platings, G.P. Shields, J.S. Stevens, M. Towler, P.A. Wood, *J. Appl. Cryst.* **2020**, *53*, 226-235.

[10] H.M. Rietveld, *J. Appl. Cryst.* ***1969***, *2*, 65-71.

[11] D. Louer, A. Boultif, *Z. Kristallogr. Suppl.* **2006**, *2006*, 225-230.

[12] G. Prandini, A. Marrazzo, I.E. Castelli, N. Mounet, N. Marzari, *Npj Comput. Mater.* ***2018***, *4*, 1-13.

[13] S. Grimme, J. Antony, S. Ehrlich, H. Krieg, *J. Chem. Phys.* **2010**, *132*, 154104.

[14] J. van de Streek, M.A. Neumann, *Acta Cryst B* **2014**, *70*, 1020-1032.

[15] A.D.J. Becke, *Chem. Phys.* **1993**, *98*, 5648.

[16] M.J. Frisch, G.W. Trucks, H.B. Schlegel, G.E. Scuseria, M.A. Robb, J.R. Cheeseman, J.A.J. Montgomery, T. Vreven, K.N. Kudin, J.C. Burant, J.M. Millam, S.S.T.J. Iyengar, V. Barone, B. Mennucci, M. Cossi, G. Scalmani, N. Rega, G.A. Petersson, H. Nakatsuji, M. Hada, M. Ehara, K. Toyota, R. Fukuda, J. Hasegawa, M. Ishida, T. Nakajima, Y. Honda, O. Kitao, H. Nakai, M. Klene, X. Li, J.E. Knox, H.P. Hratchian, J.B. Cross, V. Bakken, C. Adamo, J. Jaramillo, R. Gomperts, R.E. Stratmann, O. Yazyev, A.J. Austin, R. Cammi, C.S. Pomelli, J.W. Ochterski, P.Y. Ayala, K. Morokuma, G.A. Voth, P. Salvador, J.J. Dannenberg, V.G. Zakrzewski, S. Dapprich, A.D. Daniels, M.C. Strain, O. Farkas, D.K. Malick, A.D. Rabuck, K. Raghavachari, J.B. Foresman, J.V. Ortiz, Q. Cui, A.G. Baboul, S. Clifford, J. Cioslowski, B.B. Stefanov, G. Liu, A. Liashenko, P. Piskorz, I. Komaromi, R.L. Martin, D.J. Fox, T. Keith, M.A. Al-Laham, C.Y. Peng, A. Nanayakkara, M. Challacombe, P.M.W. Gill, B. Johnson, W. Chen, M.W. Wong, C. Gonzalez, J.A. Pople, Gaussian, Inc., Wallingford CT, 2009.

[17] P. Fuentealba, H. Preuss, H. Stoll, L.V. Szentpaly, *Chem. Phys. Lett.* **1982**, *89*, 418-422.

[18] M. Cossi, N. Rega, G. Scalmani, V. Barone, *J. Comput. Chem.* **2003**, *24*, 669-681.

[19] R. A. Sheldon, *Green Chem.* **2017**, *19*, 18-43.

Copies of HRMS Spectra

***N*-Butyl-3-(hex-1-yn-1-yl)thiophene-2-carboxamide (1a)**

HRMS-ESI [(M+H)^+^]: *m/z* calcd. for (C_15_H_22_NOS)^+^: 264.1417; found, 264.1444

*N*-Butyl-3-(3,3-dimethylbut-1-yn-1-yl)thiophene-2-carboxamide (1b)

HRMS-ESI [(M+H)^+^]: *m/z* calcd. for (C_15_H_22_NOS)^+^: 264.1417; found, 264.1424

*N*-Butyl-3-(phenylethynyl)thiophene-2-carboxamide (1c)

HRMS-ESI [(M+H)^+^]: *m/z* calcd. for (C_17_H_18_NOS)^+^: 284.1104; found, 284.1117

***N*-Butyl-3-(*p*-tolylethynyl)thiophene-2-carboxamide (1d)**

HRMS-ESI [(M+H)^+^]: *m/z* calcd. for (C_18_H_20_NOS)^+^: 298.1260; found, 298.1282

*N*-Butyl-3-((4-chlorophenyl)ethynyl)thiophene-2-carboxamide (1e)

HRMS-ESI [(M+H)^+^]: *m/z* calcd. for (C_17_H_17_NOS)^+^: 318.0714; found, 318.0726

***N*-Butyl-3-(thiophen-3-ylethynyl)thiophene-2-carboxamide (1f)**

HRMS-ESI [(M+H)^+^]: *m/z* calcd. for (C_15_H_16_NOS)^+^: 290.0668; found, 290.0663


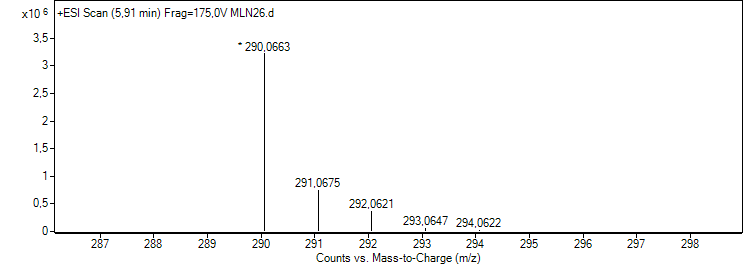


***N*-Butyl-3-(cyclohex-1-en-1-ylethynyl)thiophene-2-carboxamide (1g)**

HRMS-ESI [(M+H)^+^]: *m/z* calcd. for (C_17_H_22_NOS)^+^: 288.1417; found, 288.1426

***N*-Benzyl-3-(hex-1-yn-1-yl)thiophene-2-carboxamide (1h)**

HRMS-ESI [(M+H)^+^]: *m/z* calcd. for (C_18_H_20_NOS)^+^: 298.1260; found, 298.1267


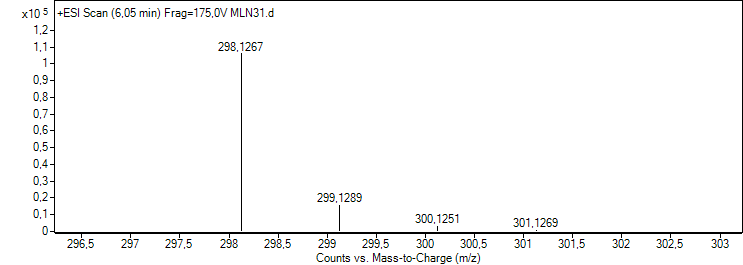


***N*-Benzyl-3-(phenyethynyl)thiophene-2-carboxamide (1i)**

HRMS-ESI [(M+H)^+^]: *m/z* calcd. for (C_20_H_16_NOS)^+^: 318.0947; found, 318.0950


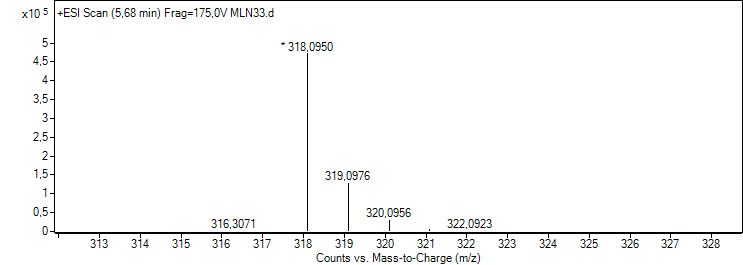


***N*-(*tert*-Butyl)-3-(hex-1-yn-1-yl)thiophene-2-carboxamide (1j)**

HRMS-ESI [(M+H)^+^]: *m/z* calcd. for (C_15_H_22_NOS)^+^: 264.1417; found, 264.1419


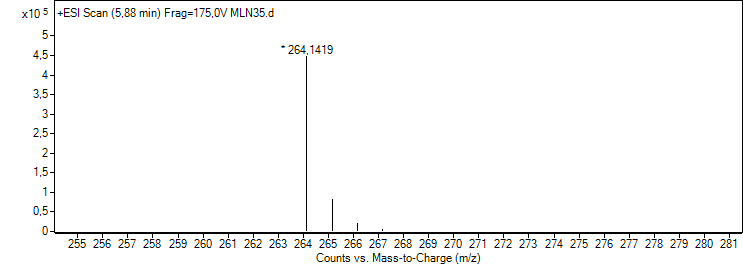


***N*-(*tert*-Butyl)-3-(phenylethynyl)thiophene-2-carboxamide (1k)**

HRMS-ESI [(M+H)^+^]: *m/z* calcd. for (C_17_H_18_NOS)^+^: 284.1104; found, 284.1100


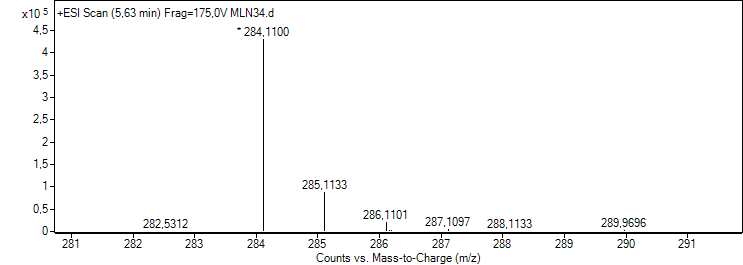


**3-(Hex-1-yn-1-yl)-*N*-phenylthiophene-2-carboxamide (1l)**

HRMS-ESI [(M+H)^+^]: *m/z* calcd. for (C_17_H_18_NOS)^+^: 284.1104; found, 284.1120

**3-(3,3-Dimethylbut-1-yn-1-yl)-*N*-phenylthiophene-2-carboxamide (1m)**

HRMS-ESI [(M+H)^+^]: *m/z* calcd. for (C_17_H_18_NOS)^+^: 284.1104; found, 284.1113

***N*-phenyl-3-(phenylethynyl)thiophene-2-carboxamide (1n)**

HRMS-ESI [(M+H)^+^]: *m/z* calcd. for (C_19_H_14_NOS)^+^: 304.0791; found, 304.0800

***N*-phenyl-3-(*p*-tolylethynyl)thiophene-2-carboxamide (1o)**

HRMS-ESI [(M+H)^+^]: *m/z* calcd. for (C_20_H_16_NOS)^+^: 318.0947; found, 318.0946

**3-((4-Chlorophenyl)ethynyl)-*N*-phenylthiophene-2-carboxamide (1p)**

HRMS-ESI [(M+H)^+^]: *m/z* calcd. for (C_19_H_13_ClNOS)^+^: 338.0401; found, 338.0406

**3-(Cyclohex-1-en-1-ylethynyl)-*N*-phenylthiophene-2-carboxamide (1q)**

HRMS-ESI [(M+H)^+^]: *m/z* calcd. for (C_19_H_18_NOS)^+^: 308.1104; found, 308.1112

***N*-(4-*tert*-Butyl)phenyl)-3-(hex-1-yn-1-yl)thiophene-2-carboxamide (1r)**

HRMS-ESI [(M+H)^+^]: *m/z* calcd. for (C_21_H_26_NOS)^+^: 340.1730; found, 340.1737


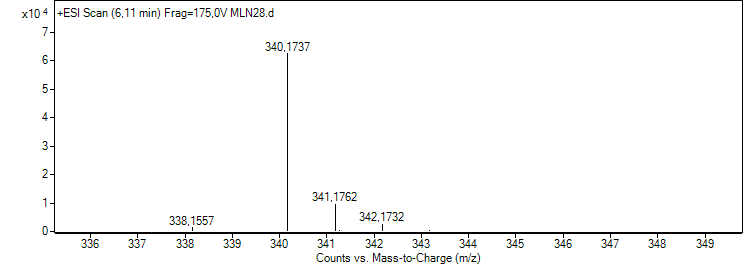


***N*-(4-*tert-*Butyl)phenyl)-3-(phenylethynyl)thiophene-2-carboxamide (1s)**

HRMS-ESI [(M+H)^+^]: *m/z* calcd. for (C_23_H_22_NOS)^+^: 360.1417; found, 360.1426


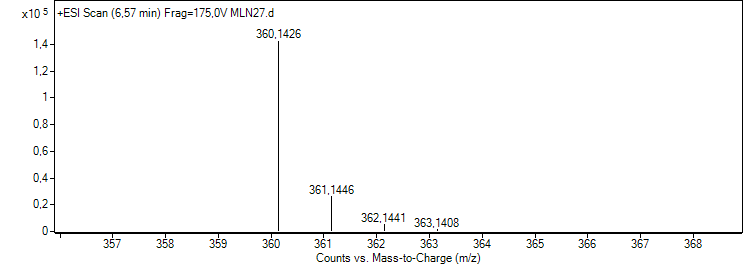


***(Z*)-*N*,5-dibutyl-4-iodo-7*H*-thieno[2,3-*c*]pyran-7-imine (2a)**

HRMS-ESI [(M+H)^+^]: *m/z* calcd. for (C_15_H_21_INOS)^+^: 390.0383; found, 390.0417

**(*Z*)-5-(*tert*-Butyl)-*N*-butyl-4-iodo-7*H*-thieno[2,3-*c*]pyran-7-imine (2b)**

HRMS-ESI [(M+H)^+^]: *m/z* calcd. for (C_15_H_21_INOS)^+^: 390.0383; found, 390.0378


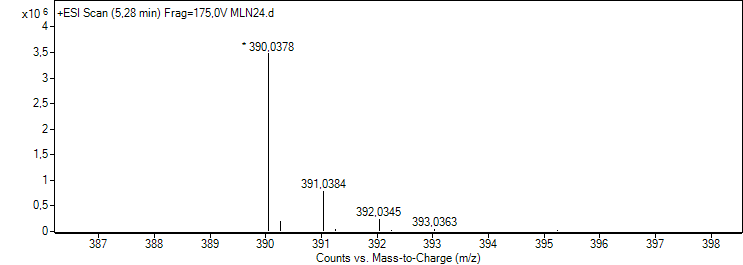


**(*Z*)-*N*-Butyl-4-iodo-5-phenyl-7*H*-thieno[2,3-*c*]pyran-7-imine (2c)**

HRMS-ESI [(M+H)^+^]: *m/z* calcd. for (C_17_H_17_INOS)^+^: 410.0070; found, 410.0069


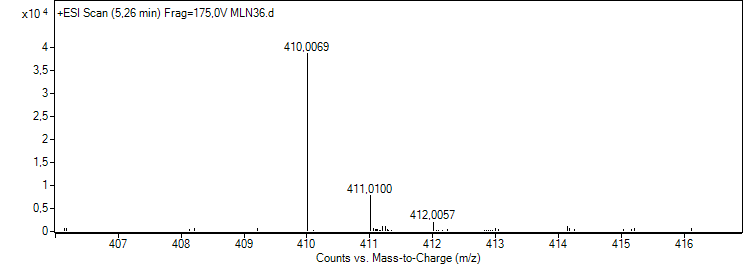


**(*Z*)-*N*-Butyl-4-iodo-5-(*p*-tolyl)-7H-thieno[2,3-c]pyran-7-imine (2d)**

HRMS-ESI [(M+H)^+^]: *m/z* calcd. for (C_18_H_19_INOS)^+^: 424.0227; found, 424.0211


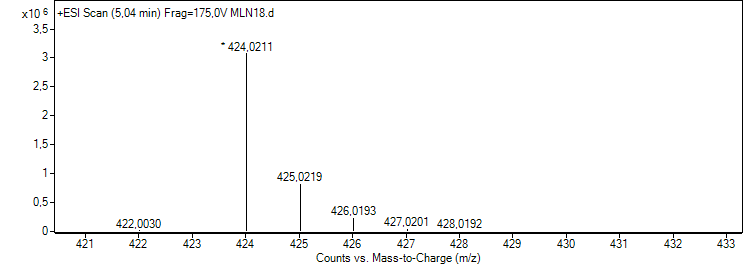


**(*Z*)-*N*-Butyl-5-(4-chloro)-4-iodo-5-(*p*-tolyl)-7*H*-thieno[2,3-*c*]pyran-7-imine (2e)**

HRMS-ESI [(M+H)^+^]: *m/z* calcd. for (C_17_H_16_ClINOS)^+^: 443.9680; found, 443.9671


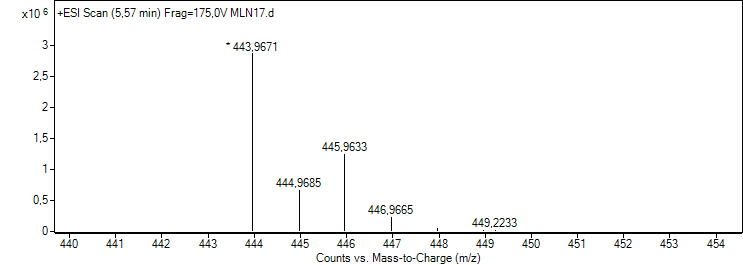


**(*Z*)-*N*-Butyl-4-iodo-5-(thiophen-3-yl)-*7H*-thieno[2,3-*c*]pyran-7-imine (2f)**

HRMS-ESI [(M+H)^+^]: *m/z* calcd. for (C_15_H_15_INOS_2_)^+^: 415.9634; found, 415.9636

**
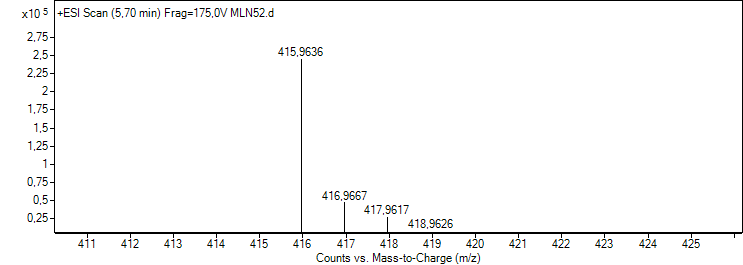
**

**(*Z*)-*N*-Butyl-5-(cyclohex-1-en-1-yl)-4-iodo-7*H*-thieno[2,3-*c*]pyran-7-imine (2g)**

HRMS-ESI [(M+H)^+^]: *m/z* calcd. for (C_17_H_21_INOS)^+^: 414.0383; found, 414.0374

**
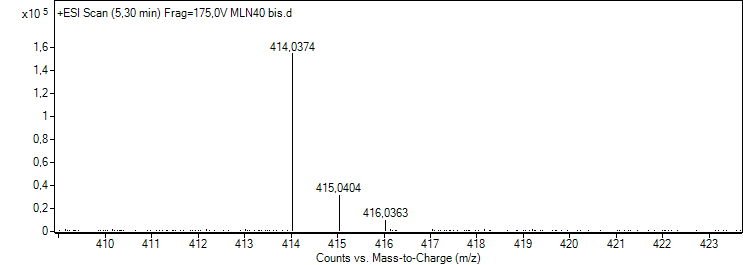
**

**(*Z*)-*N*-Benzyl-5-butyl-4-iodo-7*H*-thieno[2,3-*c*]pyran-7-imine (2h)**

HRMS-ESI [(M+H)^+^]: *m/z* calcd. for (C_18_H_19_INOS)^+^: 424.0227; found, 424.0235

**
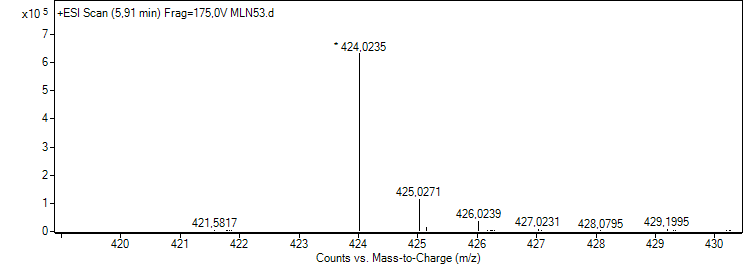
**

**(*Z*)-*N*-Benzyl-4-iodo-5-phenyl-7*H*-thieno[2,3-*c*]pyran-7-imine (2i)**

HRMS-ESI [(M+H)^+^]: *m/z* calcd. for (C_20_H_15_INOS)^+^: 443.9914~~27~~; found, 443.9923

**
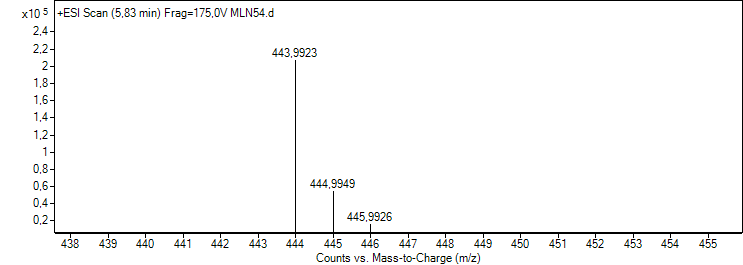
**

**(*Z*)-*N*-(*tert*-Butyl)-5-butyl-4-iodo-7*H*-thieno[2,3-*c*]pyran-7-imine (2j)**

HRMS-ESI [(M+H)^+^]: *m/z* calcd. for (C_15_H_21_INOS)^+^: 390.0383; found, 390.0385

**
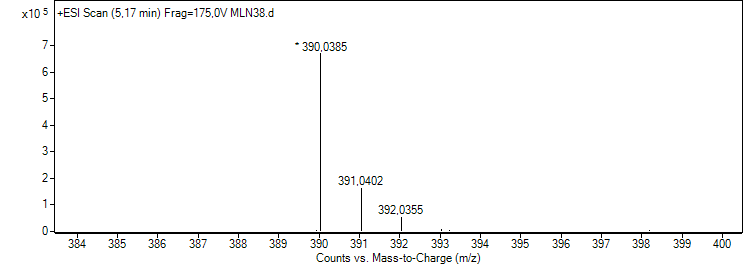
**

**(*Z*)-*N*-(*tert*-Butyl)-4-iodo-5-phenyl-7*H*-thieno[2,3-*c*]pyran-7-imine (2k)**

HRMS-ESI [(M+H)^+^]: *m/z* calcd. for (C_17_H_17_INOS)^+^: 410.0070; found, 410.0068

**
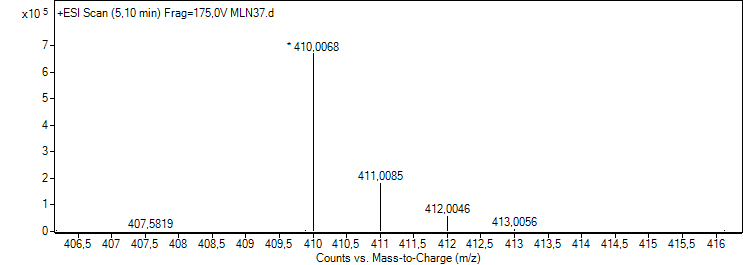
**

**(*Z*)-5-Butyl-4-iodo-*N*-phenyl-7*H*-thieno[2,3-*c*]pyran-7-imine (2l)**

HRMS-ESI [(M+H)^+^]: *m/z* calcd. for (C_17_H_17_INOS)^+^: 410.0070; found, 410.0076


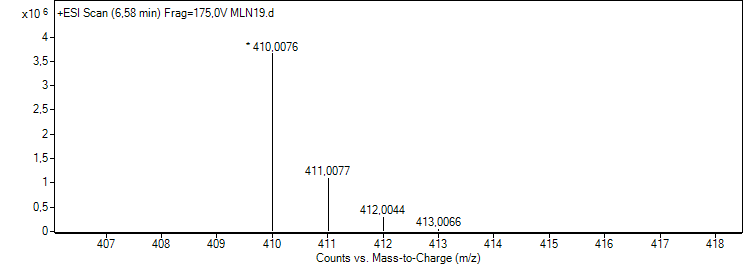


**(*Z*)-5-(*tert*-Butyl-4-iodo-*N-*phenyl-7*H*-thieno[2,3*-c*]pyran-7-imine (2m)**

HRMS-ESI [(M+H)^+^]: *m/z* calcd. for (C_17_H_17_INOS)^+^: 410.0070; found, 410.0059


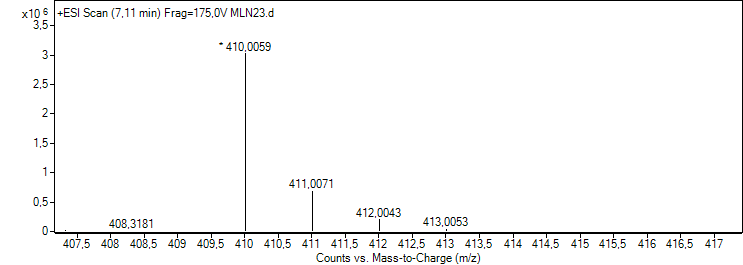


**(*Z*)-4-Iodo-*N*,5-diphenyl-7*H*-thieno[2,3-*c*]pyran-7-imine (2n)**

HRMS-ESI [(M+H)^+^]: *m/z* calcd. for (C_19_H_13_INOS)^+^: 429.9757; found, 429.9741


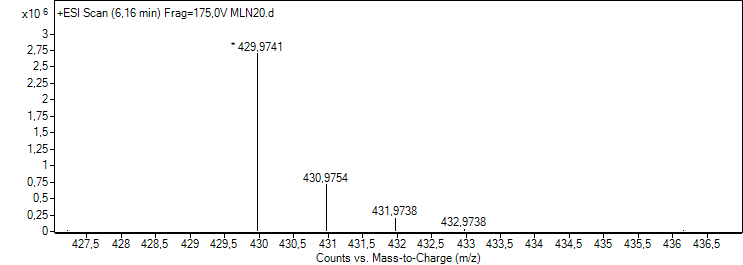


**(*Z*)-4-Iodo-*N*-phenyl-5-(*p*-tolyl)-7*H*-thieno[2,3-*c*]pyran-7-imine (2o)**

HRMS-ESI [(M+H)^+^]: *m/z* calcd. for (C_20_H_15_INOS)^+^: 443.9914; found, 443.9908


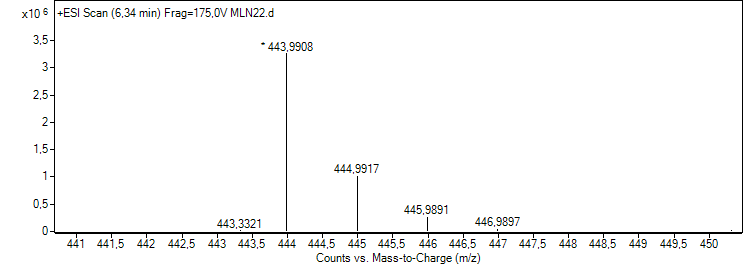


**(*Z*)-5-(4-chlorophenyl)-4-Iodo-*N*-phenyl-7*H*-thieno[2,3*-c*]pyran-7-imine (2p)**

HRMS-ESI [(M+H)^+^]: *m/z* calcd. for (C_19_H_12_ClINOS)^+^: 463.9367; found, 463.9356


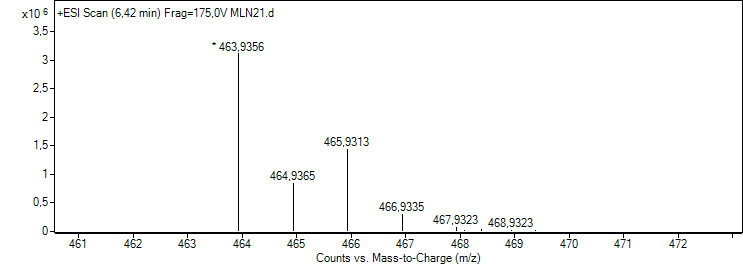


**(*Z*)-5-(Cyclohex-1-en-1-yl)-4-Iodo-*N*-phenyl-7*H*-thieno[2,3-*c*]pyran-7-imine (2q)**

HRMS-ESI [(M+H)^+^]: *m/z* calcd. for (C_19_H_17_INOS)^+^: 434.0070; found, 434.0088


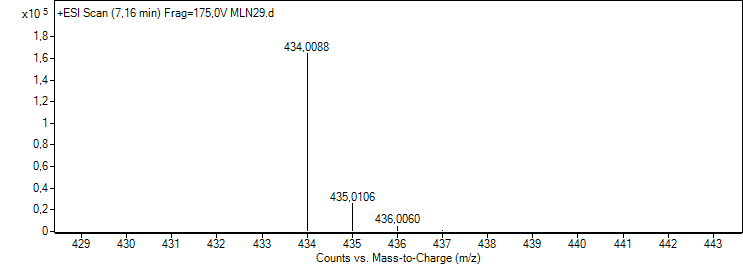


**(*Z*)-5-Butyl-*N*-(4-(*tert*-butyl)phenyl)-4-iodo-*N*-phenyl-7*H*-thieno[2,3-*c*]pyran-7-imine (2r)**

HRMS-ESI [(M+H)^+^]: *m/z* calcd. for (C_21_H_25_INOS)^+^: 466.0696; found, 466.0682

**
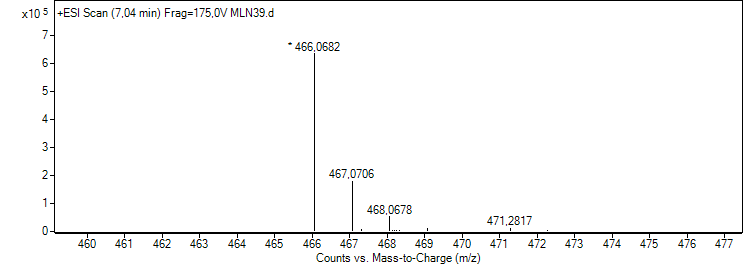
**

**(*Z*)-*N*-(4-(*tert*-butyl)phenyl)-4-iodo-5-phenyl-7*H*-thieno[2,3-*c*]pyran-7-imine (2s)**

HRMS-ESI [(M+H)^+^]: *m/z* calcd. for (C_23_H_21_INOS)^+^: 486.0383; found, 486.0399


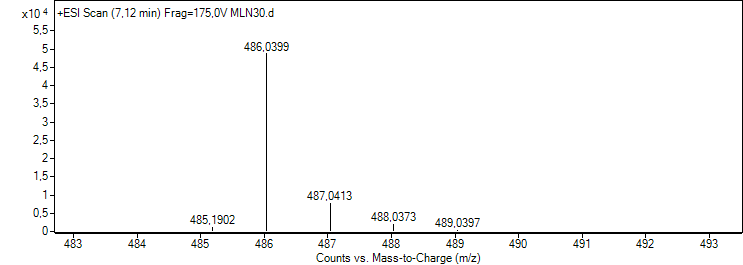


**(*Z*)-*N*,5-Dibutyl-4-(phenylethynyl)-7*H*-thieno[2,3-*c*]pyran-7-imine (4aa)**

HRMS-ESI [(M+H)^+^]: *m/z* calcd. for (C_23_H_26_NOS)^+^: 364.1730; found, 364.1728

**
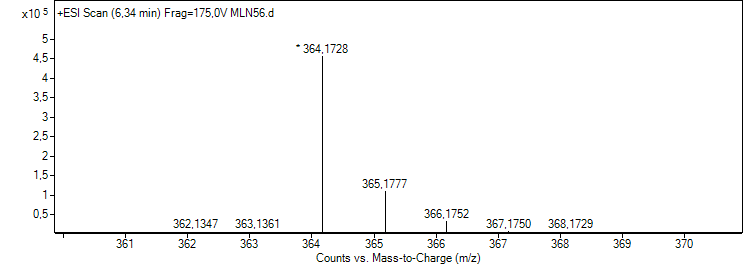
**

**(*Z*)-5-Butyl-*N*-phenyl-4-(phenylethynyl)-7*H*-thieno[2,3-*c*]pyran-7-imine (4la)**

HRMS-ESI [(M+H)^+^]: *m/z* calcd. for (C_25_H_22_NOS)^+^: 384.1417; found, 384.1412


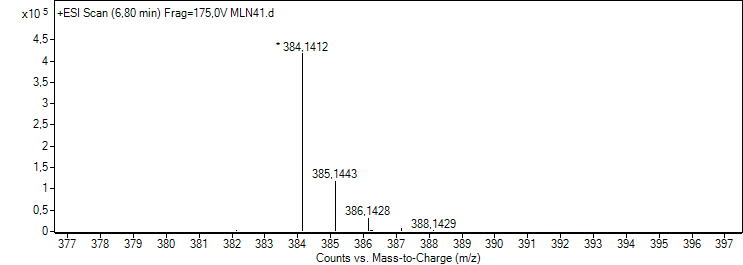


**(*Z*)-5-Butyl-*N*-phenyl-4-(thiophen-3-ylethynyl)-7*H*-thieno[2,3-*c*]pyran-7-imine (4lb)**

HRMS-ESI [(M+H)^+^]: *m/z* calcd. for (C_23_H_20_NOS_2_)^+^: 390.0981; found, 390.0974

**
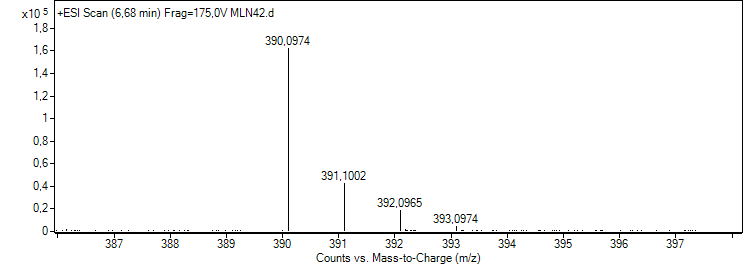
**

**(*Z*)-5-Butyl-4-(hex-1-yn-1-yl)-*N*-phenyl-7*H*-thieno[2,3-*c*]pyran-7-imine (4lc)**

HRMS-ESI [(M+H)^+^]: *m/z* calcd. for (C_23_H_26_NOS)^+^: 364.1730; found, 364.1729

**
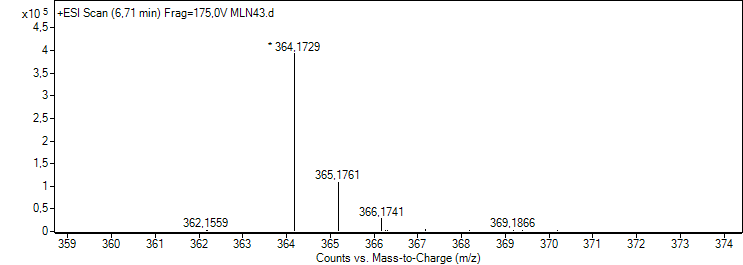
**

**(*Z*)-5-(*tert*-Butyl)-*N*-phenyl-4-(phenylethynyl)-7*H*-thieno[2,3-*c*]pyran-7-imine (4ma)**

HRMS-ESI [(M+H)^+^]: *m/z* calcd. for (C_25_H_22_NOS)^+^: 384.1417; found, 384.1412

**
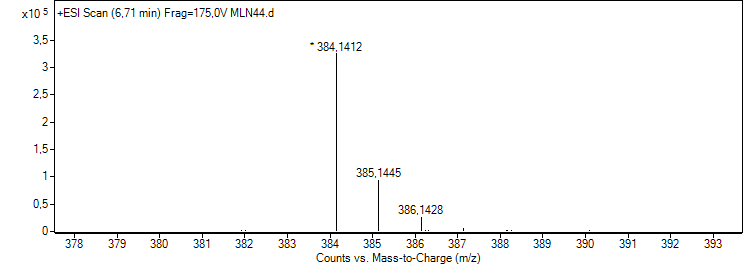
**

**(*Z*)-5-(*tert*-Butyl)-4-(cyclohex-1-en-1-ylethynyl)-*N*-phenyl-7*H*-thieno[2,3-*c*]pyran-7-imine (4md)**

HRMS-ESI [(M+H)^+^]: *m/z* calcd. for (C_25_H_26_NOS)^+^: 388.1730; found, 388.1731


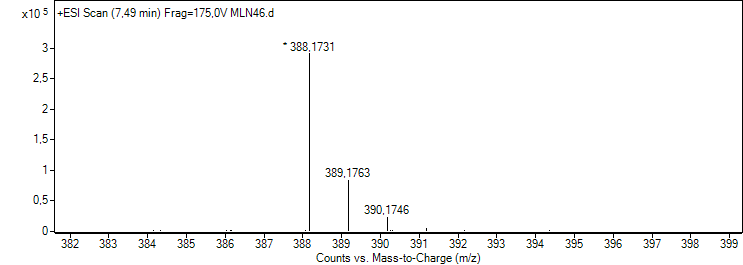


**(*Z*)-*N*,5-(Diphenyl)-4-(phenylethynyl)-7*H*-thieno[2,3-*c*]pyran-7-imine (4na)**

HRMS-ESI [(M+H)^+^]: *m/z* calcd. for (C_27_H_18_NOS)^+^: 404.1104; found, 404.1102


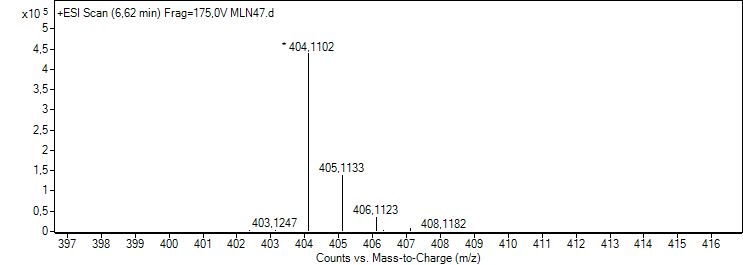


**(*Z*)-*N*,5-Diphenyl-4-(*p*-tolylethynyl)-7*H*-thieno[2,3-*c*]pyran-7-imine (4ne)**

HRMS-ESI [(M+H)^+^]: *m/z* calcd. for (C_28_H_20_NOS)^+^: 418.1260; found, 418.1260


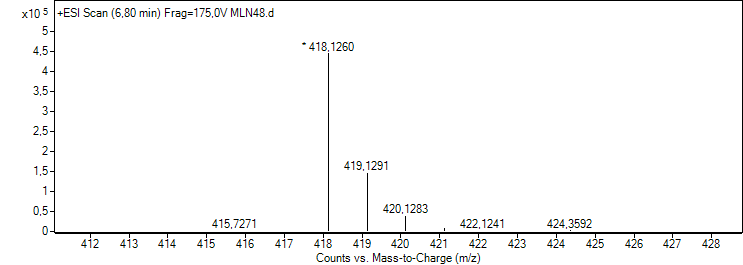


**(*Z*)-5-Butyl-*N*,4-phenyl-7*H*-thieno[2,3-*c*]pyran-7-imine (6la)**

HRMS-ESI [(M+H)^+^]: *m/z* calcd. for (C_23_H_22_NOS)^+^: 360.1417; found, 360.1413


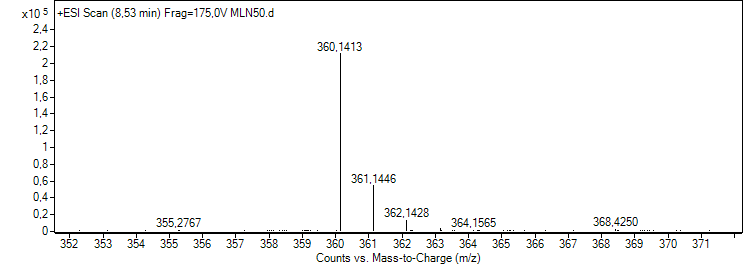


**(*Z*)-5-Butyl-*N*-phenyl-4-(*p*-tolyl)-7*H*-thieno[2,3-*c*]pyran-7-imine (6lb)**

HRMS-ESI [(M+H)^+^]: *m/z* calcd. for (C_24_H_24_NOS)^+^: 374.1573; found, 374.1575

**
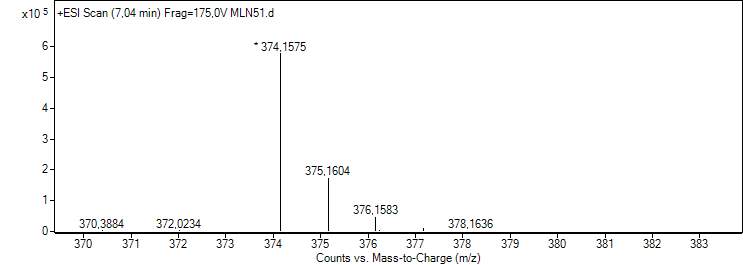
**

**(*Z*)-*N*,4,5-Triphenyl-7*H*-thieno[2,3-*c*]pyran-7-imine (6na)**

HRMS-ESI [(M+H)^+^]: *m/z* calcd. for (C_25_H_18_NOS)^+^: 380.1104; found, 380.1123


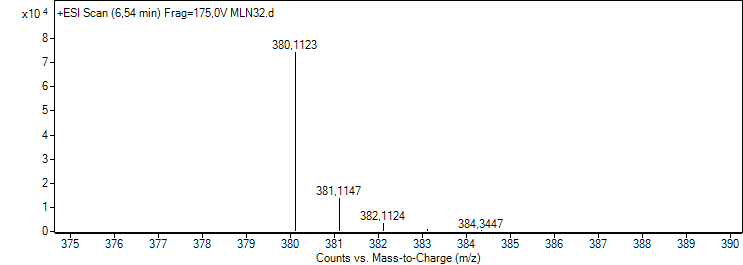


**(*Z*)-4-(furan-3-yl)-*N*,5-diphenyl-7*H*-thieno[2,3-*c*]pyran-7-imine (6nc)**

HRMS-ESI [(M+H)^+^]: *m/z* calcd. for (C_23_H_16_NO_2_S)^+^: 370.0896; found, 370.0895

**
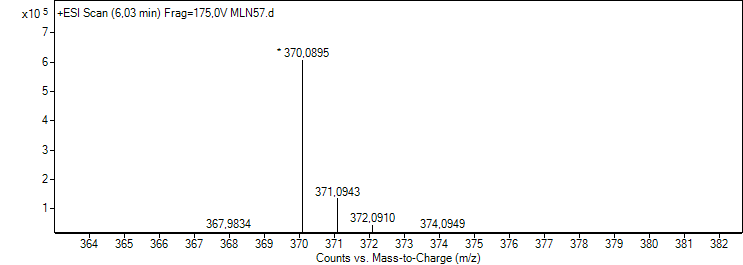
**

**Copies of ^1^H NMR and ^13^CNMR Spectra**

*N*-Butyl-3-(hex-1-yn-1-yl)thiophene-2-carboxamide (**1a**)

^1^H NMR (CDCl_3_, 500 MHz)

*N*-Butyl-3-(hex-1-yn-1-yl)thiophene-2-carboxamide (**1a**)

^13^C NMR (CDCl_3_, 125 MHz)

*N*-Butyl-3-(3,3-dimethylbut-1-yn-1-yl)thiophene-2-carboxamide (**1b**)

^1^H NMR (CDCl_3_, 500 MHz)

*N*-Butyl-3-(3,3-dimethylbut-1-yn-1-yl)thiophene-2-carboxamide (**1b**)

^13^C NMR (CDCl_3_, 125 MHz)

*N*-Butyl-3-(phenylethynyl)thiophene-2-carboxamide (**1c**)

^1^H NMR (CDCl_3_, 500 MHz)

*N*-Butyl-3-(phenylethynyl)thiophene-2-carboxamide (**1c**)

^13^C NMR (CDCl_3_, 125 MHz)

*N-*Butyl-3-(p-tolylethynyl)thiophene-2-carboxamide (**1d**)

^1^H NMR (CDCl_3_, 500 MHz)

*N-*Butyl-3-(p-tolylethynyl)thiophene-2-carboxamide (**1d**)

^13^C NMR (CDCl_3_, 125 MHz)

*N*-Butyl-3-((4-chlorophenyl)ethynyl)thiophene-2-carboxamide (**1e**)

^1^H NMR (CDCl_3_, 500 MHz)

*N*-Butyl-3-((4-chlorophenyl)ethynyl)thiophene-2-carboxamide (**1e**)

^13^C NMR (CDCl_3_, 125 MHz)

*N-*Butyl-3-(thiophen-3-ylethynyl)thiophene-2-carboxamide (**1f**)

^1^H NMR (CDCl_3_, 500 MHz)

*N-*Butyl-3-(thiophen-3-ylethynyl)thiophene-2-carboxamide (**1f**)

^13^C NMR (CDCl_3_, 125 MHz)

*N-*Butyl-3-(cyclohex-1-en-1-ylethynyl)thiophene-2-carboxamide (**1g**)

^1^H NMR (CDCl_3_, 500 MHz)

*N-*Butyl-3-(cyclohex-1-en-1-ylethynyl)thiophene-2-carboxamide (**1g**)

^13^C NMR (CDCl_3_, 125 MHz)

*N-*Benzyl-3-(hex-1-yn-1-yl)thiophene-2-carboxamide (**1h**)

^1^H NMR (CDCl_3_, 500 MHz)

*N-*Benzyl-3-(hex-1-yn-1-yl)thiophene-2-carboxamide (**1h**)

^13^C NMR (CDCl_3_, 125 MHz)

*N-*Benzyl-3-(phenyethynyl)thiophene-2-carboxamide (**1i**)

^1^H NMR (CDCl_3_, 500 MHz)

*N-*Benzyl-3-(phenyethynyl)thiophene-2-carboxamide (**1i**)

^13^C NMR (CDCl_3_, 125 MHz)

*N-*(*tert-*Butyl)-3-(hex-1-yn-1-yl)thiophene-2-carboxamide (**1j**)

^1^H NMR (CDCl_3_, 500 MHz)

*N-*(*tert-*Butyl)-3-(hex-1-yn-1-yl)thiophene-2-carboxamide (**1j**)

^13^C NMR (CDCl_3_, 125 MHz)

*N-*(*tert-*Butyl)-3-(phenylethynyl)thiophene-2-carboxamide (**1k**)

^1^H NMR (CDCl_3_, 500 MHz)

*N-*(*tert-*Butyl)-3-(phenylethynyl)thiophene-2-carboxamide (**1k**)

^13^C NMR (CDCl_3_, 125 MHz)

3-(Hex-1-yn-1-yl)-*N*-phenylthiophene-2-carboxamide (**1l**)

^1^H NMR (CDCl_3_, 500 MHz)

3-(Hex-1-yn-1-yl)-*N*-phenylthiophene-2-carboxamide (**1l**)

^13^C NMR (CDCl_3_, 125 MHz)

3-(3,3-Dimethylbut-1-yn-1-yl)-*N-*phenylthiophene-2-carboxamide (**1m**)

^1^H NMR (CDCl_3_, 500 MHz)

3-(3,3-Dimethylbut-1-yn-1-yl)-*N-*phenylthiophene-2-carboxamide (**1m**)

^13^C NMR (CDCl_3_, 125 MHz)

*N-*phenyl-3-(phenylethynyl)thiophene-2-carboxamide (**1n**)

^1^H NMR (CDCl_3_, 500 MHz)

*N-*phenyl-3-(phenylethynyl)thiophene-2-carboxamide (**1n**)

^13^C NMR (CDCl_3_, 125 MHz)

*N-*phenyl-3-(*p*-tolylethynyl)thiophene-2-carboxamide (**1o**)

^1^H NMR (CDCl_3_, 500 MHz)

*N-*phenyl-3-(*p*-tolylethynyl)thiophene-2-carboxamide (**1o**)

^13^C NMR (CDCl_3_, 125 MHz)

3-((4-Chlorophenyl)ethynyl)-*N-*phenylthiophene-2-carboxamide (**1p**)

^1^H NMR (CDCl_3_, 500 MHz)

3-((4-Chlorophenyl)ethynyl)-*N-*phenylthiophene-2-carboxamide (**1p**)

^13^C NMR (CDCl_3_, 125 MHz)

3-(Cyclohex-1-en-1-ylethynyl)-*N-*phenylthiophene-2-carboxamide (**1q**)

^1^H NMR (CDCl_3_, 500 MHz)

3-(Cyclohex-1-en-1-ylethynyl)-*N-*phenylthiophene-2-carboxamide (**1q**)

^13^C NMR (CDCl_3_, 125 MHz)

*N-*(4*-tert-*Butyl)phenyl)-3-(hex-1-yn-1-yl)thiophene-2-carboxamide (**1r**)

^1^H NMR (CDCl_3_, 500 MHz)

*N-*(4*-tert-*Butyl)phenyl)-3-(hex-1-yn-1-yl)thiophene-2-carboxamide (**1r**)

^13^C NMR (CDCl_3_, 125 MHz)

*N*-(4-*tert-*Butyl)phenyl)-3-(phenylethynyl)thiophene-2-carboxamide (**1s**)

^1^H NMR (CDCl_3_, 500 MHz)

*N*-(4-*tert-*Butyl)phenyl)-3-(phenylethynyl)thiophene-2-carboxamide (**1s**)

^13^C NMR (CDCl_3_, 125 MHz)

(*Z*)-*N,*5-dibutyl-4-iodo-7*H*-thieno[2,3-*c*]pyran-7-imine (**2a**)

^1^H NMR (CDCl_3_, 500 MHz)

(*Z*)-*N,*5-dibutyl-4-iodo-7*H*-thieno[2,3-*c*]pyran-7-imine (**2a**)

^13^C NMR (CDCl_3_, 125 MHz)

(*Z*)-5-(*tert*-Butyl)-*N-*butyl-4-iodo-7*H*-thieno[2,3-*c*]pyran-7-imine (**2b**)

^1^H NMR (CDCl_3_, 500 MHz)

(*Z*)-5-(*tert*-Butyl)-*N-*butyl-4-iodo-7*H*-thieno[2,3-*c*]pyran-7-imine (**2b**)

^13^C NMR (CDCl_3_, 125 MHz)

(*Z*)*-N-*Butyl-4-iodo-5-phenyl-7*H-thieno*[2,3-*c*]pyran-7-imine (**2c**)

^1^H NMR (CDCl_3_, 500 MHz)

(*Z*)*-N-*Butyl-4-iodo-5-phenyl-7*H-thieno*[2,3-*c*]pyran-7-imine (**2c**)

^13^C NMR (CDCl_3_, 125 MHz)

(*Z*)-*N-*Butyl-4-iodo-5-(*p*-tolyl)-7*H*-thieno[2,3*-c*]pyran-7-imine (**2d**)

^1^H NMR (CDCl_3_, 500 MHz)

(*Z*)-*N-*Butyl-4-iodo-5-(*p*-tolyl)-7*H*-thieno[2,3*-c*]pyran-7-imine (**2d**)

^13^C NMR (CDCl_3_, 125 MHz)

(Z)-*N*-butyl-5-(4-chlorophenyl)-4-iodo-7*H*-thieno[2,3-*c*]pyran-7-imine (**2e**)

^1^H NMR (CDCl_3_, 500 MHz)

(Z)-*N*-butyl-5-(4-chlorophenyl)-4-iodo-7*H*-thieno[2,3-*c*]pyran-7-imine (**2e**)

^13^C NMR (CDCl_3_, 125 MHz)

(*Z*)*-N-*Butyl-4-iodo-5-(thiophen-3-yl)-7*H*-thieno[2,3-*c*]pyran-7-imine (**2f**)

^1^H NMR (CDCl_3_, 500 MHz)

(*Z*)*-N-*Butyl-4-iodo-5-(thiophen-3-yl)-7*H*-thieno[2,3-*c*]pyran-7-imine (**2f**)

^13^C NMR (CDCl_3_, 125 MHz)

(*Z)-N-*Butyl-5-(cyclohex-1-en-1-yl)-4-iodo-7*H-*thieno[2,3*-c*]pyran-7-imine (**2g**)

^1^H NMR (CDCl_3_, 500 MHz)

(*Z)-N-*Butyl-5-(cyclohex-1-en-1-yl)-4-iodo-7*H-*thieno[2,3*-c*]pyran-7-imine (**2g**)

^13^C NMR (CDCl_3_, 125 MHz)

(*Z*)*-N*-Benzyl-5-butyl-4-iodo-7*H-*thieno[2,3-*c*]pyran-7-imine (**2h**)

^1^H NMR (CDCl_3_, 500 MHz)

(*Z*)*-N*-Benzyl-5-butyl-4-iodo-7*H-*thieno[2,3-*c*]pyran-7-imine (**2h**)

^13^C NMR (CDCl_3_, 125 MHz)

(*Z*)-*N*-Benzyl-4-iodo-5-phenyl-7*H-*thieno[2,3*-c*]pyran-7-imine (**2i**)

^1^H NMR (CDCl_3_, 500 MHz)

(*Z*)-*N*-Benzyl-4-iodo-5-phenyl-7*H-*thieno[2,3*-c*]pyran-7-imine (**2i**)

^13^C NMR (CDCl_3_, 125 MHz)

(*Z*)-*N-(tert-*Butyl)-5-butyl-4-iodo-7*H-*thieno[2,3*-c*]pyran-7-imine (**2j**)

^1^H NMR (CDCl_3_, 500 MHz)

(*Z*)-*N-(tert-*Butyl)-5-butyl-4-iodo-7*H-*thieno[2,3*-c*]pyran-7-imine (**2j**)

^13^C NMR (CDCl_3_, 125 MHz)

(*Z*)*-N-*(*tert-*Butyl)-4-iodo-5-phenyl-7*H-*thieno[2,3*-c*]pyran-7-imine (**2k**)

^1^H NMR (CDCl_3_, 500 MHz)

(*Z*)*-N-*(*tert-*Butyl)-4-iodo-5-phenyl-7*H-*thieno[2,3*-c*]pyran-7-imine (**2k**)

^13^C NMR (CDCl_3_, 125 MHz)

(*Z*)-5-Butyl-4-iodo-*N-*phenyl-7*H-*thieno[2,3*-c*]pyran-7-imine (**2l**)

^1^H NMR (CDCl_3_, 500 MHz)

(*Z*)-5-Butyl-4-iodo-*N-*phenyl-7*H-*thieno[2,3*-c*]pyran-7-imine (**2l**)

^13^C NMR (CDCl_3_, 125 MHz)

(*Z*)-5-(*tert-*Butyl-4-iodo-*N-*phenyl-7*H-*thieno[2,3*-c*]pyran-7-imine (**2m**)

^1^H NMR (CDCl_3_, 500 MHz)

(*Z*)-5-(*tert-*Butyl-4-iodo-*N-*phenyl-7*H-*thieno[2,3*-c*]pyran-7-imine (**2m**)

^13^C NMR (CDCl_3_, 125 MHz)

(*Z*)-4-Iodo-*N,*5-diphenyl-7*H-*thieno[2,3*-c*]pyran-7-imine (**2n**)

^1^H NMR (CDCl_3_, 500 MHz)

(*Z*)-4-Iodo-*N,*5-diphenyl-7*H-*thieno[2,3*-c*]pyran-7-imine (**2n**)

^13^C NMR (CDCl_3_, 125 MHz)

(*Z*)-4-Iodo-*N*-phenyl-5-(*p-*tolyl)-7*H-*thieno[2,3*-c*]pyran-7-imine (**2o**)

^1^H NMR (CDCl_3_, 500 MHz)

(*Z*)-4-Iodo-*N*-phenyl-5-(*p-*tolyl)-7*H-*thieno[2,3*-c*]pyran-7-imine (**2o**)

^13^C NMR (CDCl_3_, 125 MHz)

(*Z*)-5-(4-chlorophenyl)-4-Iodo-*N-*phenyl-7*H-*thieno[2,3*-c*]pyran-7-imine (**2p**)

^1^H NMR (CDCl_3_, 500 MHz)

(*Z*)-5-(4-chlorophenyl)-4-Iodo-*N-*phenyl-7*H-*thieno[2,3*-c*]pyran-7-imine (**2p**)

^13^C NMR (CDCl_3_, 125 MHz)

(*Z*)-5-(Cyclohex-1-en-1-yl)-4-Iodo*-N*-phenyl-7*H-*thieno[2,3*-c*]pyran-7-imine (**2q**)

^1^H NMR (CDCl_3_, 500 MHz)

(*Z*)-5-(Cyclohex-1-en-1-yl)-4-Iodo*-N*-phenyl-7*H-*thieno[2,3*-c*]pyran-7-imine (**2q**)

^13^C NMR (CDCl_3_, 125 MHz)

(*Z*)-5-Butyl-*N*-(4-(*tert-*butyl)phenyl)-4-iodo-*N-*phenyl-7*H-*thieno[2,3-*c*]pyran-7-imine (**2r**)

^1^H NMR (CDCl_3_, 500 MHz)

(*Z*)-5-Butyl-*N*-(4-(*tert-*butyl)phenyl)-4-iodo-*N-*phenyl-7*H-*thieno[2,3-*c*]pyran-7-imine (**2r**)

^13^C NMR (CDCl_3_, 125 MHz)

(*Z*)-*N*-(4-(*tert-*butyl)phenyl)-4-iodo-5-phenyl-7*H-*thieno[2,3*-c*]pyran-7-imine (**2s**)

^1^H NMR (CDCl_3_, 500 MHz)

(*Z*)-*N*-(4-(*tert-*butyl)phenyl)-4-iodo-5-phenyl-7*H-*thieno[2,3*-c*]pyran-7-imine (**2s**)

^13^C NMR (CDCl_3_, 125 MHz)

(*Z*)-*N*,5-Dibutyl-4-(phenylethynyl)-7*H-*thieno[2,3*-c*]pyran-7-imine (**4aa**)

^1^H NMR (CDCl_3_, 500 MHz)

(*Z*)-*N*,5-Dibutyl-4-(phenylethynyl)-7*H-*thieno[2,3*-c*]pyran-7-imine (**4aa**)

^13^C NMR (CDCl_3_, 125 MHz)

(*Z*)*-*5-Butyl*-N*-phenyl-4-(phenylethynyl)-7*H-*thieno[2,3*-c*]pyran-7-imine (**4la**)

^1^H NMR (CDCl_3_, 500 MHz)

(*Z*)*-*5-Butyl*-N*-phenyl-4-(phenylethynyl)-7*H-*thieno[2,3*-c*]pyran-7-imine (**4la**)

^13^C NMR (CDCl_3_, 125 MHz)

(*Z*)-5-Butyl-*N-*phenyl-4-(thiophen-3-ylethynyl)-7*H-*thieno[2,3*-c*]pyran-7-imine (**4lb**)

^1^H NMR (CDCl_3_, 500 MHz)

(*Z*)-5-Butyl-*N-*phenyl-4-(thiophen-3-ylethynyl)-7*H-*thieno[2,3*-c*]pyran-7-imine (**4lb**)

^13^C NMR (CDCl_3_, 125 MHz)

(*Z*)-5-Butyl-4-(hex-1-yn-1-yl)*-N-*phenyl-7*H-*thieno[2,3-*c*]pyran-7-imine (**4lc**)

^1^H NMR (CDCl_3_, 500 MHz)

(*Z*)-5-Butyl-4-(hex-1-yn-1-yl)*-N-*phenyl-7*H-*thieno[2,3-*c*]pyran-7-imine (**4lc**)

^13^C NMR (CDCl_3_, 125 MHz)

(*Z*)-5-(*tert*-Butyl)-*N-*phenyl-4-(phenylethynyl)-7*H-*thieno[2,3*-c*]pyran-7-imine (**4ma**)

^1^H NMR (CDCl_3_, 500 MHz)

(*Z*)-5-(*tert*-Butyl)-*N-*phenyl-4-(phenylethynyl)-7*H-*thieno[2,3*-c*]pyran-7-imine (**4ma**)

^13^C NMR (CDCl_3_, 125 MHz)

(*Z*)-5-(*tert-*Butyl)-4-(cyclohex-1-en-1-ylethynyl)-*N-*phenyl-7*H-*thieno[2,3*-c*]pyran-7-imine (**4md**)

^1^H NMR (CDCl_3_, 500 MHz)

(*Z*)-5-(*tert-*Butyl)-4-(cyclohex-1-en-1-ylethynyl)-*N-*phenyl-7*H-*thieno[2,3*-c*]pyran-7-imine (**4md**)

^13^C NMR (CDCl_3_, 125 MHz)

(*Z)-N,*5-(Diphenyl)-4-(phenylethynyl)-7*H-*thieno[2,3*-c*]pyran-7-imine (**4na**)

^1^H NMR (CDCl_3_, 500 MHz)

(*Z)-N,*5-(Diphenyl)-4-(phenylethynyl)-7*H-*thieno[2,3*-c*]pyran-7-imine (**4na**)

^13^C NMR (CDCl_3_, 125 MHz)

(*Z*)*-N,*5-Diphenyl-4-(*p*-tolylethynyl)-7*H-*thieno[2,3-*c*]pyran-7-imine (**4ne**)

^1^H NMR (CDCl_3_, 500 MHz)

(*Z*)*-N,*5-Diphenyl-4-(*p*-tolylethynyl)-7*H-*thieno[2,3-*c*]pyran-7-imine (**4ne**)

^13^C NMR (CDCl_3_, 125 MHz)

(*Z*)-5-Butyl-*N,*4-phenyl-7*H-*thieno[2,3*-c*]pyran-7-imine (**6la**)

^1^H NMR (CDCl_3_, 500 MHz)

(*Z*)-5-Butyl-*N,*4-phenyl-7*H-*thieno[2,3*-c*]pyran-7-imine (**6la**)

^13^C NMR (CDCl_3_, 125 MHz)

(*Z*)-5-Butyl-*N-*phenyl-4-(*p*-tolyl)-7*H-*thieno[2,3-*c*]pyran-7-imine (**6lb**)

^1^H NMR (CDCl_3_, 500 MHz)

(*Z*)-5-Butyl-*N-*phenyl-4-(*p*-tolyl)-7*H-*thieno[2,3-*c*]pyran-7-imine (**6lb**)

^13^C NMR (CDCl_3_, 125 MHz)

*(Z*)-*N,*4,5-Triphenyl-7*H-*thieno[2,3*-c*]pyran-7-imine (**6na**)

^1^H NMR (CDCl_3_, 500 MHz)

*(Z*)-*N,*4,5-Triphenyl-7*H-*thieno[2,3*-c*]pyran-7-imine (**6na**)

^13^C NMR (CDCl_3_, 125 MHz)

(*Z*)-4-(furan-3-yl)-*N*,5-diphenyl-7*H*-thieno[2,3-*c*]pyran-7-imine (**6nc**)

^1^H NMR (CDCl_3_, 500 MHz)

(*Z*)-4-(furan-3-yl)-*N*,5-diphenyl-7*H*-thieno[2,3-*c*]pyran-7-imine (**6nc**)

^13^C NMR (CDCl_3_, 125 MHz)
